# The Immune Microenvironment in Liver Cancer: From Analysis to Targeting

**DOI:** 10.1002/advs.202518487

**Published:** 2025-11-07

**Authors:** Jiaming Lan, Hao Li, Jian Xue, Yourong Duan, Jin Sun, Meng Niu

**Affiliations:** ^1^ Department of Interventional Radiology Shengjing Hospital of China Medical University Shenyang Liaoning 110004 China; ^2^ China Medical University Shenyang Liaoning 110122 China; ^3^ State Key Laboratory of Oncogenes and Related Genes Shanghai Cancer Institute Renji Hospital School of Medicine Shanghai Jiao Tong University Shanghai 200032 China; ^4^ Wuya College of Innovation Shenyang Pharmaceutical University Shenyang Liaoning 110016 China; ^5^ Joint International Research Laboratory of Intelligent Drug Delivery Systems Ministry of Education Shenyang Liaoning 110016 China

**Keywords:** immune reprogramming, liver cancer, nanomedicines, targeted delivery, tumor microenvironment

## Abstract

Despite advancements in early detection and treatment, liver cancer (LC) remains highly recurrent due to its complex immunosuppressive tumor microenvironment (TME), leading to poor prognosis in advanced stages. Nanomedicines (NMs) offer novel therapeutic strategies for reversing the immunosuppressive TME in LC. This review systematically analysed the diverse mechanisms contributing to immunosuppressive TME formation and explored the potential of smart responsive NMs in targeted drug delivery, immune remodeling, and multimodal therapy. The immunosuppressive TME in LC arises from abnormal physiological conditions, extracellular matrix (ECM) deposition, dysfunction of antigen‐presenting cells, exhaustion of T cells, infiltration of immunosuppressive cells, metabolic reprogramming, and microbiota influences. Smart NMs can overcome delivery barriers through passive targeting and ligand‐directed active targeting to LC cells via receptors, as well as to immunosuppressive cell populations. NMs can respond to endogenous and exogenous stimuli, enabling precise spatiotemporal drug release. This feature enables integration of chemotherapy, immunotherapy, and physical therapies. Additionally, NMs can reprogram the TME by remodeling physiological conditions, inhibiting ECM deposition, regulating metabolism, inducing immunogenic cell death, and modulating microbiota‐derived metabolites. Although toxicity and clinical translation still require further optimization, smart NMs offer a paradigm shift for LC therapy through an integrated “targeted delivery‐immune reprogramming” strategy.

## Graphical abstract

This review explores how smart nanomedicines overcome the immunosuppressive tumor microenvironment in liver cancer. It details targeted delivery strategies and immune reprogramming mechanisms, including remodeling abnormal physiology, modulating metabolism, and inducing immunogenic cell death. The article highlights the paradigm shift toward multimodal synergistic therapies, offering novel insights for advancing precision immunotherapy.

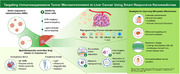

## 1 Introduction

Liver cancer (LC) is the sixth most common cancer globally and the third leading cause of cancer‐related deaths, with a 5‐year survival rate of only 18%.^[^ ^1^, ^2^ ^]^ Currently, clinical treatment strategies for LC involve a combination of local therapies such as liver transplantation, resection, radiofrequency ablation, transarterial chemoembolization (TACE), and radiological embolization, along with chemotherapy and radiotherapy (RT). However, tumor heterogeneity and multidrug resistance result in a recurrence rate of up to 70% after initial treatment. Moreover, conventional chemotherapy drugs induce severe systemic toxicity because of their nonspecific distribution, further limiting their efficacy.^[^ ^3^, ^4^ ^]^ For example, whereas targeted drugs such as sorafenib (Sor) can extend survival in advanced‐stage patients, only ≈30% of patients benefit from it, and this group typically develops resistance within 6 months.^[^ ^5^ ^]^

As LC treatment enters the era of immunotherapy, programmed death receptor 1 (PD‐1)‐targeted immune checkpoint inhibitors (ICIs), such as pembrolizumab and nivolumab, have been approved by the U.S. Food and Drug Administration (FDA) as second‐line treatments for advanced LC. However, these drugs have not shown a significant improvement in overall survival when compared to the findings regarding Sor.^[^ ^6^, ^7^ ^]^ In 2020, the phase III IMBRAVE‐150 study (NCT03434379) showed that the combination of the anti–programmed death receptor ligand 1 (PD‐L1) drug atezolizumab and the anti–vascular endothelial growth factor (VEGF) drug bevacizumab was superior to Sor, with a median progression‐free survival of 6.8 months in the combination group versus 4.3 months in the Sor group. As a result, the combination therapy was approved by the FDA as a first‐line treatment for advanced LC.^[^ ^8^ ^]^ Nonetheless, despite these unprecedented and encouraging results, only 20–30% of patients respond to immunotherapy.^[^ ^9^ ^]^ The immunosuppressive microenvironment of LC is a key factor responsible for treatment failure. The polarization of tumor‐associated macrophages (TAMs) to the M2 phenotype, overexpression of PD‐1/PD‐L1 immune checkpoints, and T‐cell exhaustion contribute to the “cold tumor” characteristics, leading to a low response rate to immunotherapy.^[^ ^10^ ^]^ In addition, the presence of cancer stem cells (CSCs) in LC, through pathways such as Want/β‐catenin, maintains tumor stemness and exacerbates resistance and metastasis risks.^[^ ^11^ ^]^ From a safety perspective, administering high doses of cytokines and ICIs to enhance the efficacy of immunotherapies may lead to significant autoimmune side effects and systemic toxicity. For example, interleukin (IL) therapy can induce cytokine release syndrome and capillary leak syndrome.^[^ ^12^, ^13^ ^]^ Studies have shown that single‐agent therapies are insufficient to simultaneously overcome multiple challenges, such as microenvironmental heterogeneity, CSC resistance, and immune evasion.

To address these issues in LC treatment, a promising approach involves leveraging the functional integration properties of nanomedicines (NMs). This review innovatively proposes a “targeted delivery‐immune reprogramming” synergistic strategy, representing a paradigm shift in LC management. In the field of drug delivery, NMs are therapeutic agents formulated from polymers, lipids, inorganic NMs, or natural biomaterials, with approximate sizes of ≤100 nm. NMs are increasingly used in LC immunotherapy to enhance the efficacy of immunosuppressants, while reducing their toxicity.^[^ ^14^ ^]^ Beyond conventional drug delivery, our focus extends to smart NMs capable of dynamically remodeling the immunosuppressive microenvironment. By targeting drug delivery and integrating immune modulation, multimodal synergistic therapy can achieve enhanced therapeutic effects. NMs, with EPR effects and active targeting modifications, can significantly increase drug accumulation at tumor sites, thereby reducing systemic toxicity.^[^ ^15^ ^]^ For example, lactobionic acid (LA)‐modified NMs target the asialoglycoprotein receptor (ASGPR) receptors, which are highly expressed on LC cells, enabling stepwise delivery of chemotherapeutic drugs and simultaneous magnetic resonance imaging (MRI)‐guided imaging using iron oxide NMs.^[^ ^16^ ^]^ Moreover, pH/redox‐responsive NMs can control drug release according to the characteristics of the tumor microenvironment (TME) and combine near‐infrared (NIR) imaging for integrated diagnosis and treatment.^[^ ^17^ ^]^ A key innovative aspect highlighted in this review is the development of stimulus‐responsive “smart” NMs that can trigger precise drug release in response to endogenous stimuli such as pH, enzymes, and reactive oxygen species (ROS), or exogenous stimuli such as light and magnetic fields, thus overcoming the spatial and temporal limitations of traditional drug delivery methods.^[^ ^18^, ^19^, ^20^ ^]^ For instance, acid‐activated smart NMs can deliver both the photosensitizer PPa and PD‐L1 siRNA, reducing dark toxicity in photodynamic therapy (PDT) and enhancing T‐cell infiltration by blocking the PD‐1/PD‐L1 pathway.^[^ ^21^ ^]^ Such innovative designs represent a transformative approach that integrates chemotherapy, immune modulation, and imaging technologies, providing a new paradigm for precision medicine.

This review provides a comprehensive analysis of the latest advancements in smart, responsive NMs for overcoming LC heterogeneity, resistance, and immunosuppression, with particular emphasis on their innovative mechanisms and design principles. We systematically summarize the synergistic strategy of targeted delivery‐immune reprogramming and explore novel pathways for clinical translation through multimodal synergy, such as the combination of photothermal therapy (PTT), chemotherapy, and immunotherapy, as well as emerging biomimetic technologies such as cell membrane camouflage and exosome engineering. These innovative approaches offer a groundbreaking theoretical framework and technical insights for future research, potentially revolutionizing LC treatment paradigms.

## 2 Analysis of the Immune Microenvironment Features of LC and Its Treatment Challenges

The liver, a crucial organ responsible for metabolism, immune response, and detoxification, is continuously exposed to antigens originating from the gut during its physiological processes. To maintain internal balance and proper function, the liver establishes an immunosuppressive microenvironment composed of hepatocytes, immune cells, and stromal cells.^[^ ^22^ ^]^ Under normal conditions, dendritic cells (DCs), Kupffer cells (KCs), and natural killer (NK) cells in the hepatic sinusoids predominantly exhibit a tolerant phenotype, with minimal response to antigenic stimulation. These cells, in conjunction with resident Tregs and MDSCs in the sinusoids, as well as hepatic stellate cells (HSCs) in the perisinusoidal space, secrete immunosuppressive factors to maintain immune system balance.^[^ ^23^ ^]^ However, the immunosuppressive microenvironment of the liver provides a favorable niche for tumor growth, metastasis, and recurrence.

**Figure 1:**
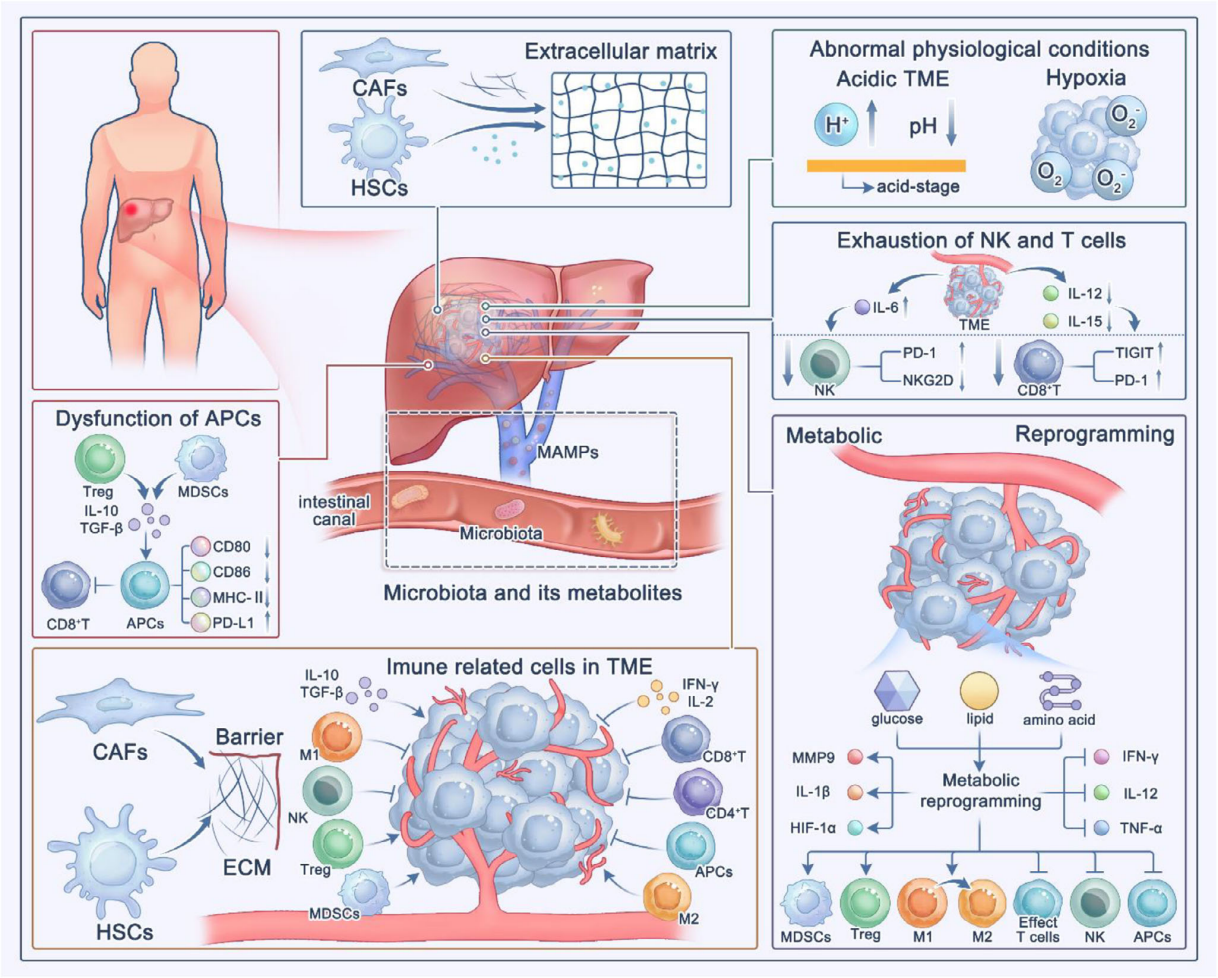
Characteristics of the immunosuppressive TME in LC, including abnormal physiological conditions, dysfunctional DCs, infiltration of immunosuppressive cells, exhaustion of NK and T cells, excessive ECM deposition, metabolic reprogramming, and microbiota‐mediated immune suppression.

The TME is a dynamic and complex ecosystem comprising both cellular and acellular components. The cells involved include infiltrating immune cells as well as other stromal cells and acellular elements produced by these cells, such as cytokines, growth factors, and ECM proteins associated with inflammation.^[^ ^24^ ^]^ The immune microenvironment of LC is characterized by abnormal physiological conditions, dysfunctional DC cells, infiltration of immunosuppressive cells, exhaustion of NK and T cells, excessive ECM deposition, metabolic reprogramming, and abnormal vasculature. Recent studies have also highlighted that dysbiosis of the gut microbiome may mediate immune evasion (**Figure** **1**).^[^ ^25^, ^26^ ^]^

### 2.1 Abnormal Physiological Conditions

In the LC microenvironment, abnormal physiological conditions such as low pH and hypoxia are critical drivers of tumor malignancy, as well as provide potential targets for therapeutic strategy development.^[^ ^27^, ^28^ ^]^ LC cells primarily rely on glycolysis for energy production through the Warburg effect, generating large amounts of lactate even under normoxic conditions, which acidifies the TME. This phenomenon is associated with multiple signaling pathways, such as the abnormal activation of the Wnt/β‐catenin pathway, which upregulates the expression of key glycolytic enzymes such as HK2 and LDHA, further promoting lactate production.^[^ ^29^ ^]^ In addition, cancer‐associated fibroblasts (CAFs) secrete ECM proteins that impede lactate diffusion, exacerbating the local acidic environment. Low pH promotes the malignant phenotype of tumors through multiple mechanisms.^[^ ^30^ ^]^ The acidic environment activates matrix metalloproteinases (MMPs), degrading the ECM and facilitating tumor cell invasion and metastasis, while also suppressing the function of T cells and NK cells, thereby creating an immune‐suppressive microenvironment.^[^ ^31^ ^]^ Studies have also shown that low pH induces drug resistance in tumor cells by modulating ion channels such as the proton pump NHE1, reducing the efficacy of chemotherapy.^[^ ^32^ ^]^ The acidic microenvironment in LC has inspired recent drug and NMs designs. For instance, the use of monocarboxylate transporter inhibitors to block lactate production or transport can reduce the acidity of the LC microenvironment.^[^ ^33^ ^]^ In addition, carbonic anhydrase inhibitors such as acetazolamide can decrease the conversion of CO2 to protons.^[^ ^34^ ^]^ Recent studies have also explored pH‐responsive NMs delivery systems, which achieve targeted therapy by specifically addressing acidic regions. Zhang et al. developed a pH‐responsive drug delivery system (PN@GPBPEG NMs) loaded with the chemotherapeutic agent paclitaxel (PTX) and the indoleamine 2,3‐dioxygenase (IDO) inhibitor NLG919 for chemophotodynamic immunotherapy of LC. Experimental results showed that PN@GPB‐PEG NMs exhibited pH‐responsive characteristics, enhanced tumor targeting ability, and increased LC cell uptake. The PTX carried by this NM induces immunogenic cell death (ICD), activating the immune system to generate an antitumor response. Notably, the NLG919 inhibitor suppresses IDO, alleviating the immunosuppressive environment. This approach effectively achieves targeted delivery and immune modulation for LC treatment.^[^ ^35^ ^]^

Rapid proliferation of LC tissue leads to insufficient blood perfusion, resulting in hypoxic core regions with oxygen partial pressure below 10 mmHg.^[^ ^36^ ^]^ Hypoxia‐inducible factor 1α (HIF‐1α) is a key regulatory molecule whose stability is controlled by oxygen‐dependent prolyl hydroxylases (PHDs). Under hypoxic conditions, PHDs are inactivated, allowing HIF‐1α to accumulate and translocate to the nucleus, where it activates downstream target genes. Increasing evidence has shown that the hypoxic TME impairs tumor sensitivity to immunotherapy by disrupting T cell function and promoting the infiltration of immunosuppressive cells, thereby exacerbating the immune suppression in LC. Notably, hypoxia inhibits T cell differentiation and migration toward the tumor and reduces the levels of immune‐stimulatory cytokines, such as interferon‐γ (IFN‐γ) and IL‐2, thereby promoting T cell apoptosis.^[^ ^37^ ^]^ Studies have shown that notably few T cells are present in hypoxia‐induced necrotic regions of solid tumors, indicating that T cell numbers and immune responses may be negatively regulated by hypoxia. In addition, hypoxia‐activated HIFs and the accumulation of lactate mediated by hypoxia impair T cell proliferation and effector function.^[^ ^38^ ^]^ However, hypoxia and HIF‐1α upregulate VEGF and platelet‐derived growth factor (PDGF) to promote angiogenesis, but the resulting disordered neo‐vascular structures further exacerbate hypoxia.^[^ ^39^, ^40^ ^]^ Hypoxia also promotes the polarization of TAMs from the immune‐competent M1 phenotype to the immunosuppressive M2 phenotype.^[^ ^41^ ^]^ Targeting core regulatory molecules in the hypoxic region with specific NMs may help improve the hypoxic TME. Liu et al. developed a DNA and photosensitizer co‐delivery system based on carbon nitride (CNs) with oxygen‐generating functionality (BPSCN). This system achieved DNA hydrophobicity by mixing the P53 gene with a positively charged mitochondrial‐targeted NIR emitting photosensitizer (MTTPY). MTTPY was encapsulated in non‐cationic BPSCN to ultimately form BPSCNs@MTTPY‐P53. The ester bonds in this NM can be broken by lipases in the liver, facilitating the release of P53, upregulating its expression, and promoting the degradation of HIF‐1α in the mitochondria. Notably, the oxygen produced by BPSCNs@MTTPY‐P53 improves the hypoxic microenvironment of LC, synergistically downregulating HIF‐1α expression in mitochondria and promoting mitochondrial‐derived ferroptosis.^[^ ^42^ ^]^

### 2.2 ECM

ECM serves as an important physical scaffold in the surrounding environment, primarily consisting of two components, glycosaminoglycans and proteins such as collagen, elastin, fibronectin, and laminin. In addition to providing physical support, ECM plays a crucial role in maintaining tissue morphogenesis, differentiation, and homeostasis under physiological conditions by activating biochemical and biomechanical signals.^[^ ^43^, ^44^ ^]^ ECM is pivotal in the progression of LC, altering the mechanical properties and cellular signaling within the TME. Specifically, in LC, disrupted ECM contributes to increased tissue stiffness, which enhances integrin signaling and mechanotransduction pathways.^[^ ^45^ ^]^ This stiffening environment, largely because of the secretion of collagen by activated HSCs, accelerates the transition from liver fibrosis to LC. The mechanical characteristics of ECM activate cellular pathways such as FAK and YAP/TAZ, promoting tumor cell proliferation, migration, and survival.^[^ ^46^ ^]^ In addition to its structural role, ECM influences the behavior of surrounding stromal and immune cells.^[^ ^47^ ^]^ For instance, ECM components, particularly collagen and other matrix proteins, act as scaffolds for TAMs and MDSCs, which play significant roles in immune evasion. These cells typically polarize in ways that support tumor growth, exacerbating the immunosuppressive TME.^[^ ^48^, ^49^ ^]^ The complex interaction between ECM and immune cells is often a key reason for immunotherapy failure, as ECM can physically obstruct immune cell entry into tumors and impair effective immune surveillance.^[^ ^50^ ^]^

In addition to its role in tumor progression, ECM significantly impacts immune responses within the TME. Altered ECM hinders immune cell infiltration as well as regulates their activity.^[^ ^51^ ^]^ Key ECM components, such as transforming growth factor‐β (TGF‐β) and osteopontin, promote the polarization of TAMs to the M2 phenotype, weakening the activity of cytotoxic T cells and NK cells.^[^ ^52^, ^53^ ^]^ Moreover, the stiffened matrix in LC can upregulate immune checkpoint proteins such as PD‐L1, further suppressing T cell function.^[^ ^54^ ^]^ This immune evasion mechanism is exacerbated by the ability of the ECM to trap immune cells, limiting their ability to migrate toward tumor cells and decreasing their tumor‐killing efficacy.^[^ ^55^ ^]^ The interaction between ECM proteins and immune cells plays a crucial role in shaping the response to therapy, especially in ICIs treatments.^[^ ^56^ ^]^ The dense ECM in LC can form barriers, limiting the effectiveness of these therapies by restricting drug access to tumor cells. This indicated that ECM remodeling and targeted therapies may enhance ICIs efficacy.^[^ ^57^ ^]^ Understanding how ECM regulates these immune‐modulatory effects is vital for developing treatment strategies aimed at restoring effective immune surveillance and overcoming immune therapy resistance. Xiao et al. addressed the clinical challenge of T cell‐dependent immunotherapy inefficacy in LC because of ECM‐mediated cytotoxic T lymphocytes (CTLs) infiltration barriers. They developed a NM system combining a pH and MMP‐2 responsive polymer with calcium phosphate (CaP) to co‐deliver hyaluronidase (HAase), IL‐12, and an anti‐PD‐L1 antibody. The acidic TME triggered CaP dissolution, facilitating the release of IL‐12 and HAase, which degrade the ECM and enhance CTLs infiltration and proliferation. Simultaneously, the in situ release of the anti‐PD‐L1 antibody within the TME prevented tumor cells from evading CTLs‐mediated cytotoxicity. This dual‐responsive NM system provides a novel immunotherapeutic strategy for solid tumors characterized by dense ECM structures.^[^ ^58^ ^]^

### 2.3 Dysfunction of Antigen‐presenting Cells (APCs) Induces Immune Evasion

In the LC microenvironment, DCs as core APCs, are subjected to multifaceted inhibition, leading to immune evasion.^[^ ^59^ ^]^ Under physiological conditions, DCs capture tumor antigens through phagocytosis, macropinocytosis, or receptor‐mediated endocytosis.^[^ ^60^ ^]^ After processing, they present antigens to CD8^+^ or CD4^+^ T cells through major histocompatibility complex class I/II (MHC‐I/II), thereby activating specific anti‐tumor immune responses.^[^ ^61^ ^]^ However, the LC microenvironment disrupts this process through multiple mechanisms. Both tumor cells and DCs exhibit significantly upregulated expression of PD‐L1. When PD‐L1 binds to PD‐1 on T cells, it induces T cell exhaustion, manifested as a reduction in IFN‐γ secretion and loss of cytotoxic function.^[^ ^62^, ^63^ ^]^ In addition, cytotoxic T‐lymphocyte‐associated protein 4 (CTLA‐4) on T cells competes with the co‐stimulatory DC molecules CD80 and CD86, blocking the CD28‐B7 signaling pathway and inhibiting T cell activation.^[^ ^64^, ^65^ ^]^ Moreover, Tregs and MDSCs accumulate in the tumor, directly suppressing DC maturation by secreting cytokines such as TGF‐β and IL‐10. This process induces a tolerant phenotype in DCs, characterized by downregulation of CD80, CD86, and MHC‐II expression.^[^ ^66^, ^67^ ^]^ LC cells also epigenetically silence the human leukocyte antigen (HLA) gene or interfere with proteasomal function, leading to the loss of MHC‐I expression and limiting antigen presentation efficiency.^[^ ^68^ ^]^ The antigen cross‐presentation capacity of DCs is further impaired owing to dysfunction in the transporter associated with antigen processing (TAP), weakening CD8^+^ T cell recognition of tumor antigens.^[^ ^69^ ^]^

Dysfunction of APCs in the LC microenvironment involves the abnormal regulation of multiple signaling pathways. One core mechanism is the activation of the PD‐1/PD‐L1 axis. Under IFN‐γ stimulation, tumor cells upregulate PD‐L1 expression through the Janus kinase‐signal transducer and activator of transcription (JAK‐STAT) pathway. Binding of PD‐L1 to PD‐1 on T cells activates Src homology region 2 domain‐containing phosphatase‐2 (SHP2), which inhibits dephosphorylation of zeta‐chain‐associated protein kinase 70 (ZAP70) in the T‐cell receptor (TCR) signaling pathway, leading to T cell dysfunction. Anti‐PD‐1 antibodies or anti‐PD‐L1 antibodies can block this pathway and restore T cell activity.^[^ ^70^, ^71^ ^]^ In the CTLA‐4/B7 pathway, CTLA‐4 recruits protein phosphatase 2A (PP2A) through its intracellular immunoreceptor tyrosine‐based inhibitory motif (ITIM), suppressing phosphatidylinositol 3‐kinase‐protein kinase B‐mechanistic target of rapamycin (PI3K‐Akt‐mTOR) signaling downstream of TCR, while also competitively blocking the binding of CD28 to B7 molecules. Anti‐CTLA‐4 antibodies can reverse this co‐stimulatory signal inhibition.^[^ ^72^, ^73^ ^]^ In the TGF‐β/Smad pathway, phosphorylation of Sma‐ and Mad‐related proteins induces DC expression of IDO, promoting the accumulation of kynurenine, a metabolic product of tryptophan, which inhibits T cell proliferation and induces apoptosis. Targeting TGF‐β receptors with specific drugs can reverse this immune suppression.^[^ ^74^ ^]^ In the Toll‐like receptor (TLR)/nuclear factor kappa‐light‐chain‐enhancer of activated B cells (NF‐κB) pathway, damage‐associated molecular patterns (DAMPs) such as high mobility group box 1 (HMGB1) activate NF‐κB signaling in DCs through TLR4. However, chronic inflammation leads to a tolerant phenotype in DCs, manifested by decreased IL‐12 secretion and increased IL‐10 levels. Combining TLR agonists with ICIs can synergistically enhance DC maturation and antigen presentation.^[^ ^75^, ^76^ ^]^ Multi‐target intervention strategies targeting these pathways, including the combined use of antibody drugs and small molecule inhibitors, have become a research focus for remodeling APC function and enhancing anti‐tumor immune responses. NMs have emerged as efficient tools for this purpose, enabling precise drug delivery and immune system modulation. Zuo et al. developed a DC‐derived exosome (DEX)‐based vaccine (DEX_P&A2&N_) by coating DEX with a LC targeting peptide (P47‐P), an alpha‐fetoprotein epitope (AFP212‐A2), and a high‐mobility group nucleosome‐binding protein 1 (N1ND‐N) functional domain. This vaccine facilitates DC recruitment and activation. In LC‐bearing mice with a high tumor burden, DEX_P&A2&N_ induced significant tumor suppression and tumor‐specific immune responses.^[^ ^77^ ^]^

### 2.4 Metabolic Reprogramming

LC induces an immunosuppressive TME through metabolic reprogramming. In glucose metabolism, LC cells exhibit a significant Warburg effect, relying on aerobic glycolysis rather than oxidative phosphorylation for energy production.^[^ ^29^ ^]^ This is facilitated by the upregulation of key enzymes, including hexokinase 2 (HK2) and phosphoglycerate kinase 1 (PGK1), which accelerate glucose uptake and lactate production.^[^ ^78^, ^79^ ^]^ The excessive accumulation of lactate acidifies the TME and promotes tumor invasion and metastasis as well as induces M2 polarization in macrophages through activation of the GPR132 receptor on their surface, thereby suppressing the glycolytic activity and IFN‐γ secretion of CD8^+^ T cells.^[^ ^80^, ^81^ ^]^ Furthermore, lactate‐mediated histone lactylation upregulates the expression of the immune checkpoint molecule PD‐L1 in tumor cells, further promoting T cell exhaustion.^[^ ^82^ ^]^ In LC, the activity of the key enzyme succinate dehydrogenase (SDH) in the tricarboxylic acid (TCA) cycle is reduced, leading to the accumulation of succinate. Notably, succinate stabilizes HIF‐1α, activating angiogenesis pathways; however, it binds to the succinate receptor 1 (SUCNR1) on TAMs, inducing them to secrete MMP9 and pro‐inflammatory cytokine IL‐1β, which drive tumor metastasis and recruit immunosuppressive myeloid cells.^[^ ^83^, ^84^ ^]^ Recent studies have also revealed that mutations in isocitrate dehydrogenase (IDH) in LC cells lead to the abnormal accumulation of the oncogenic metabolite 2‐hydroxyglutarate (2‐HG), which impairs T cell anti‐tumor function by inhibiting their epigenetic reprogramming.^[^ ^85^ ^]^

LC reprograms lipid metabolism to drive immune evasion by altering fatty acid composition. Although linoleic acid has been shown to inhibit tumor proliferation, its levels are significantly reduced in the portal vein blood of patients with LC, whereas its derivative, arachidonic acid, is elevated. Arachidonic acid is converted to prostaglandin E2 (PGE2) through the cyclooxygenase‐2 (COX‐2) pathway, which inhibits dendritic cell maturation and promotes Treg expansion.^[^ ^86^ ^]^ Cholesterol metabolism is also critical, in LC cells, the overexpression of cholesterol esterification enzyme SOAT1 leads to abnormal accumulation of cholesterol esters in lipid droplets, forming cholesterol‐rich membrane microdomains. These microdomains activate the TLR7 signaling pathway, promoting tumor proliferation and migration.^[^ ^87^, ^88^ ^]^ Furthermore, the secondary bile acid deoxycholic acid (DCA) accumulates in the LC microenvironment, where it inhibits CD8^+^ T cell infiltration and cytotoxicity by activating the Farnesoid X receptor (FXR).^[^ ^89^ ^]^ In amino acid metabolism, LC cells upregulate indoleamine IDO1 and tryptophan‐2,3‐dioxygenase (TDO2), leading to substantial depletion of tryptophan and hindering T cell proliferation owing to tryptophan deficiency.^[^ ^90^ ^]^ In addition, tumor cells highly express glutaminase GLS1, which competitively depletes glutamine in the microenvironment, inhibiting T cell mTORC1 signaling and anti‐tumor activity.^[^ ^91^ ^]^ Notably, the loss of the urea cycle enzyme argininosuccinate synthetase 1 (ASS1) results in arginine depletion in the microenvironment, impairing T cell metabolic adaptation and activating MDSCs to secrete arginase 1 (ARG1), creating a positive feedback loop of immune suppression.^[^ ^92^, ^93^ ^]^ These metabolic disturbances collectively shape an immunosuppressive TME and provide a theoretical basis for targeting metabolic‐immune interactions with NMs. Through an in‐depth analysis of clinical samples from patients with LC resistant to Sor, scientists identified that cofilin (CFL1) was highly expressed in these patients and closely associated with poor prognosis. CFL1 promotes serine synthesis and metabolism, enhancing antioxidant production to eliminate excess ROS induced by Sor, thereby reducing LC cell sensitivity to the drug. To overcome this resistance, a redox‐responsive NM was designed for the co‐delivery of CFL1 siRNA (siCFL1) and Sor. This co‐delivery system effectively enhanced LC cell sensitivity to Sor by silencing CFL1 expression, significantly inhibiting tumor growth.^[^ ^94^ ^]^

### 2.5 Infiltration of Immunosuppressive Cells

Immune suppressive cells significantly influence the progression and therapeutic resistance of LC by remodeling the TME. Studies have indicated that TAMs, serving as core regulators of the TME, promote immune evasion through their M2 polarization phenotype through multiple mechanisms.^[^ ^95^ ^]^ For example, exosomes derived from LC cells carry factors such as GOLM1 and IL‐6, which induce high expression of PD‐L1 in TAMs, directly inhibiting CTLs.^[^ ^96^ ^]^ Moreover, M2 phenotype TAMs secrete IL‐10 and suppress IL‐12, driving the differentiation of CD4^+^ T cells into Th2 cells, further weakening the anti‐tumor immune response.^[^ ^97^ ^]^ Notably, CAFs recruit monocytes to differentiate into TAMs by secreting chemokines such as CCL2 and CSF‐1, and promote M2 polarization through TGF‐β signaling, thus creating a positive feedback immune suppression loop.^[^ ^98^, ^99^ ^]^ Preclinical experiments have confirmed that targeting key molecular points in the CAFs‐TAMs interaction axis, such as blocking the CCL2/CCR2 pathway and reprogramming TAMs to the pro‐inflammatory M1 type, significantly restores T cell activity, providing a theoretical foundation for multi‐target immunotherapy.^[^ ^100^, ^101^ ^]^

The accumulation of MDSCs is closely associated with reduced efficacy of ICIs in patients with LC. Mechanistically, MDSCs consume L‐arginine in the microenvironment through ARG1 and inducible nitric oxide synthase (iNOS), thereby blocking T cell metabolism and proliferation.^[^ ^102^ ^]^ In addition, the secretion of TGF‐β and IL‐10 by MDSCiption 3 (STAT3) pathway, inducing the expansion of Tregs and promoting M2 polarization of TAMs, thereby forming a cascading immune suppression network.^[^ ^103^ ^]^ Tregs further inhibit CD8+ T cell function through CTLA‐4 and CD25‐dependent mechanisms, and collaborate with CAFs to upregulate VEGF expression, promoting angiogenesis and immune evasion. Notably, inhibitors targeting key nodes in the MDSCs‐Tregs interaction axis, such as the ROS/STAT3 pathway or IDO pathway, have been shown to significantly reduce Treg infiltration and restore T cell activity in preclinical models. These findings indicate that combined strategies targeting both MDSCs and Tregs may provide a promising approach to reverse immune therapy resistance in LC.^[^ ^104^, ^105^ ^]^ For instance, Chen et al. developed a photosensitive, dual‐targeting NMs (M.RGD@Cr‐CTS‐siYTHDF1 NMs). This NM consists of a DSPE‐modified RGD peptide shell that targets integrin receptors on tumor cells and a carboxymethyl mannose moiety that targets CD206 receptors on macrophages. The core is composed of chitosan (CS), which adsorbs m6A reader protein YTHDF1 siRNA and chromium NMs. M.RGD@Cr‐CTS‐siYTHDF1 effectively suppresses the production of the immunosuppressive cytokine IL‐10, while upregulating the expression of immune‐stimulatory factors, including IL‐12 and IFN‐γ. Consequently, this leads to enhanced CD8+ T cell infiltration and a concurrent reduction in the infiltration of Tregs and MDSCs within the TME.^[^ ^106^ ^]^

### 2.6 Exhaustion of NK Cells and T Cells

NK cells and T cells, particularly CD8^+^ CTLs, are the primary immune effector cells in innate and adaptive immunity, respectively. However, despite their potent cytotoxic capabilities, tumor cells and the TME reduce their tumor infiltration and impair their functional efficacy, leading to immune exhaustion. NK and T cell activation relies on the integrated balance between activating and inhibitory signals mediated by cell surface receptors. During the development of LC, this balance is disrupted.^[^ ^107^, ^108^ ^]^ For example, the downregulation of the critical activating receptor NKG2D on NK cells marks their exhaustion and dysfunction.^[^ ^109^ ^]^ However, NK cells and T cells also exhibit persistent upregulation of various inhibitory receptors, including PD‐1 and TIGIT, which are hallmark features of immune exhaustion in the TME.^[^ ^110^ ^]^ Notably, immune‐related cytokines, including IL‐12, IL‐15, and IL‐6, play a crucial role in cell communication within the TME and are closely linked to the cytotoxic activity of T cells and NK cells. In the TME, abnormal secretion of anti‐tumor cytokines, such as IL‐12 and IL‐15, as well as pro‐tumor cytokines, such as IL‐6, contributes to immune effector cell exhaustion.^[^ ^111^, ^112^ ^]^ Therefore, targeting these immune‐related activation/inhibition receptors or cytokines may be an effective strategy to rejuvenate anti‐tumor immune responses.

### 2.7 Microbiota and Its Metabolites Mediating Immune Suppression

Maintaining a balanced microbiota composition is crucial for establishing an ecological barrier that defends against external stimuli. The gut microbiota and mucosal immunity interact synergistically to maintain intestinal homeostasis. When this balance is disrupted, dysbiosis provides a survival advantage for pathogenic bacteria, while reducing the number of beneficial microbes.^[^ ^113^ ^]^ In LC, an imbalance in the gut microbiota composition has been observed, with significant increases in *Escherichia coli* and *Atopobium* populations, whereas *Lactobacillus*, *Bifidobacterium*, and *Enterococcus* populations are notably reduced.^[^ ^25^ ^]^ The gut microbiota plays a central role in the development of the LC microenvironment through immune evasion mechanisms mediated by dysbiosis.^[^ ^114^ ^]^ First, dysbiosis impairs intestinal barrier function, resulting in the phenomenon of leaky gut, which facilitates the entry of microbial‐associated molecular patterns (MAMPs), such as lipopolysaccharides (LPS) and lipoteichoic acid (LTA), into the liver through the portal vein. Preclinical models and patients with LC have shown significantly elevated circulating LPS levels, a phenomenon closely associated with increased gut permeability and bacterial translocation.^[^ ^115^ ^]^ LPS induces chronic inflammation and immune tolerance by activating TLR4 on liver immune cells, promoting hepatocarcinogenesis.^[^ ^116^ ^]^ Notably, LTA, as an agonist of TLR2, targets hematopoietic stem cells, inducing a senescence‐associated secretory phenotype that enhances the fibrotic and proliferative functions of HSCs.^[^ ^117^ ^]^ Furthermore, dysbiosis exacerbates the interaction between bacterial DNA, flagellin, and immune cells by disrupting intestinal epithelial tight junctions, thus creating a chronic inflammatory microenvironment.^[^ ^118^ ^]^ This inflammatory backdrop, coupled with oxidative stress, further alters the gut microbiota composition, such as promoting the enrichment of resistant bacterial strains, and activates downstream NF‐κB and MAPK signaling pathways through TLRs. These pathways contribute to angiogenesis, suppress T cell‐mediated adaptive immunity, and ultimately result in immune evasion.^[^ ^119^, ^120^ ^]^

The imbalance of gut microbiota‐derived metabolites directly regulates the immune suppressive state within the LC microenvironment. Dysbiosis leads to the overproduction of secondary bile acids, such as DCA, which, through activation of the FXR and TGR5 signaling pathways, suppresses the expression of the chemokine CXCL16, reducing the recruitment of NK cells to the liver and directly impairing NK cell cytotoxic function, including decreased granzyme B secretion.^[^ ^121^, ^122^ ^]^ In addition, short‐chain fatty acids (SCFAs), such as butyrate, regulate Treg differentiation by inhibiting histone deacetylases, further suppressing anti‐tumor immune responses.^[^ ^123^ ^]^ These metabolites also induce a senescence‐associated secretory phenotype in HSCs, releasing pro‐fibrotic factors and immunosuppressive cytokines, including TGF‐β, IL‐6, and IL‐10, thereby creating an immune suppressive microenvironment enriched in MDSCs and TAMs.^[^ ^124^ ^]^ Notably, dysbiosis is closely linked to systemic endotoxemia, aberrant activation of TLR signaling, and a vicious cycle of chronic inflammation, ultimately driving LC initiation, progression, and resistance to immunotherapy through multi‐layered interactions within the gut‐liver axis.^[^ ^125^ ^]^ Targeting the gut microbiota and its metabolites has emerged as a promising direction for recent NMs therapies, as highlighted by Ren et al. The authors developed a NM by conjugating Fe@Fe_3_O_4_ NMs with ginsenoside Rg3 (NpRg3). During the progression of LC, the application of NpRg3 delayed LC‐induced morphological changes in the cecum and alterations in gut microbiota for >12 weeks. In addition, it increased the abundance of Bacteroidetes and Verrucomicrobia, while reducing Firmicute levels. The administration of NpRg3 restructured the correlation network between gut microbiota and metabolism during LC treatment, thereby inhibiting the progression and metastasis of LC.^[^ ^126^ ^]^

### 2.8 Abnormal Vasculature and Angiogenic Mechanisms in LC

Abnormal vasculature is a hallmark feature of the LC immune TME, manifesting as a disorganized and dysfunctional vascular network. This aberrant angiogenesis is driven by an imbalance between pro‐angiogenic and anti‐angiogenic factors, involving complex and multi‐level molecular regulation.^[^ ^127^, ^128^ ^]^ The VEGF/VEGFR signaling axis plays a central role in the generally hypoxic TME, HIF‐1α accumulates and transcriptionally upregulates the expression of VEGF and its receptor VEGFR‐2. This, in turn, activates downstream pathways such as PI3K‐Akt and MAPK, promoting endothelial cell proliferation, survival, migration, and increased vascular permeability.^[^ ^129^, ^130^ ^]^ Concurrently, the Angiopoietin (Ang)/Tie2 system provides a critical balance. Ang‐1 promotes vascular maturation and stability, whereas up‐regulated Ang‐2 in tumors destabilizes vessels in the presence of VEGF, fostering the formation of immature new blood vessels.^[^ ^131^ ^]^ Furthermore, factors like PDGF and fibroblast growth factors (FGFs), along with stromal cells such as TAMs and CAFs, create a paracrine signaling loop that sustains angiogenesis by secreting inflammatory cytokines like IL‐8 and tumor necrosis factor‐α (TNF‐α). These mechanisms collectively lead to vascular abnormalities, including tortuous dilation, an incomplete basement membrane, and inadequate pericyte coverage.^[^ ^132^ ^]^ This abnormal vascular structure causes highly heterogeneous perfusion and significantly increased vascular permeability. This not only forms the basis for the EPR effect and the passive targeting of NMs but also actively maintains and exacerbates LC hypoxia and acidosis, creating a self‐reinforcing vicious cycle.

The aberrant vascular ecosystem profoundly impacts the LC TME, therapeutic response, and anti‐tumor immunity. The resulting inefficient perfusion directly causes and sustains hypoxia, which further drives high VEGF expression and angiogenesis, while also leading to lactate accumulation and extracellular acidification via the Warburg effect. This severely hinders the uniform delivery of therapeutic agents, constituting a significant physical barrier contributing to treatment resistance.^[^ ^133^ ^]^ More importantly, abnormal vasculature is a key architect of the immunosuppressive TME. The distorted blood flow and dysfunctional endothelial cells physically impede the extravasation and infiltration of immune effector cells, such as CTLs, into the tumor parenchyma. Additionally, hypoxic conditions can upregulate the expression of immune checkpoint molecules like PD‐L1 on endothelial and tumor cells, and promote the recruitment and function of immunosuppressive cells, thereby posing a major obstacle to effective immunotherapy.^[^ ^134^ ^]^

Targeting this mechanism, anti‐angiogenic therapy, by blocking the VEGF pathway, has become a cornerstone of LC treatment. Cutting‐edge research is focused on developing NMs that can actively target VEGFR or integrins, or respond to specific conditions in the tumor vasculature (e.g., specific enzymes, pH).^[^ ^135^, ^136^ ^]^ In summary, abnormal blood vessels in LC are not a passive backdrop but a dynamic core component linking tumor growth, metabolic reprogramming, immune escape, and treatment resistance. A deeper understanding of this component is crucial for developing novel drug delivery systems and combination therapy strategies.

### 2.9 Specific Surface Markers of LC Cells and Their Functional Roles in Tumor Progression

The specific receptors or markers highly expressed on the surface of LC cells are not only crucial drivers of the malignant tumor phenotype but also ideal molecular targets for targeted therapy. These receptors promote the proliferation, invasion, and metastasis of LC by regulating signal transduction, metabolic reprogramming, and immune evasion.^[^ ^137^ ^]^ A thorough understanding of their biological functions provides a theoretical basis for designing highly specific nanotechnology‐based targeted therapeutic strategies.

Glypican‐3 (GPC3) is an oncofetal proteoglycan that is absent in healthy adult liver tissues but markedly overexpressed in LC, where it acts as a key driver of tumor progression by activating multiple oncogenic signaling pathways. Its oncogenic functions include serving as a co‐receptor to potentiate Wnt/β‐catenin signaling through stabilization of ligand–receptor complexes; interacting with growth factors such as FGF2 and hepatocyte growth factor (HGF) via its heparan sulfate chains to enhance mitogenic signaling; recruiting M2‐polarized TAMs to establish an immunosuppressive microenvironment; and promoting epithelial–mesenchymal transition (EMT) by downregulating E‐cadherin to facilitate tumor invasion and metastasis.^[^ ^138^ ^]^ Owing to this multifaceted role in hepatocarcinogenesis and its tumor‐specific expression profile, GPC3 has been recognized as a highly specific biomarker for LC diagnosis, valuable for immunohistochemical differentiation from benign hepatic lesions and as a serum marker, particularly in AFP‐negative cases. Moreover, GPC3 has emerged as a promising therapeutic target, with multiple strategies currently under development, including GPC3‐targeted monoclonal antibodies (e.g., GC33/codrituzumab) that induce antibody‐dependent cellular cytotoxicity (ADCC); peptide vaccines eliciting GPC3‐specific CTLs responses; immunotoxins delivering potent cytotoxins directly to tumor cells; and advanced cellular immunotherapies using chimeric antigen receptor T cells (CAR‐T) engineered to recognize GPC3.^[^ ^139^, ^140^, ^141^ ^]^ Collectively, these GPC3‐based modalities represent targeted approaches aimed at reprogramming the immune‐tolerant microenvironment and improving clinical outcomes in LC.

ASGPR is a liver‐specific C‐type lectin receptor composed of ASGPR1 and ASGPR2 subunits that form a hetero‐oligomer, serving as a key molecular mechanism mediating hepatocyte targeting. This receptor specifically recognizes ligands containing galactose or N‐acetylgalactosamine (GalNAc) moieties, efficiently triggering clathrin‐mediated endocytosis and thereby enabling the effective internalization of drug–carrier complexes.^[^ ^142^ ^]^ Although the expression level of ASGPR in LC tissues remains somewhat controversial, numerous studies have demonstrated that ASGPR‐mediated targeted delivery systems significantly enhance the intracellular accumulation and gene‐silencing efficacy of therapeutic oligonucleotides in LC cells. GalNAc‐modified NMs, such as liposomal NMs, can selectively bind ASGPR to promote drug internalization and suppress tumor proliferation by modulating key signaling pathways, including Wnt/β‐catenin.^[^ ^143^ ^]^ Moreover, ASGPR‐based targeted delivery systems enable hepatocyte‐selective drug delivery, markedly reducing systemic toxicity and improving the therapeutic index. ASGPR serves as a hepatocyte‐specific marker for differential diagnosis of LC; in therapeutics, GalNAc‐conjugated antisense oligonucleotides enable liver‐targeted gene‐silencing therapy; and in NMs development, ASGPR ligand‐functionalized NMs enhance LC‐specific drug delivery, thereby improving antitumor efficacy while minimizing systemic toxicity.^[^ ^144^ ^]^

The CXCR4 (C‐X‐C chemokine receptor 4) and its ligand CXCL12 constitute a signaling axis that plays a pivotal role in the progression of LC. Studies have shown that CXCR4 expression is markedly upregulated in LC tissues, promoting malignant progression through multiple mechanisms. Activation of CXCR4 mediates the secretion of MMP‐2 and MMP‐9, enhancing ECM degradation and thereby facilitating tumor cell invasion and distant metastasis. The CXCL12–CXCR4 signaling axis also stimulates oval cell proliferation and induces their abnormal differentiation, contributing to hepatocarcinogenesis. Clinical studies have demonstrated that elevated CXCR4 expression is significantly associated with local tumor progression, lymphatic metastasis, and reduced patient survival, independent of the p53 pathway.^[^ ^145^ ^]^ From an application perspective, CXCR4 has been established as a key biomarker for LC prognosis, with its expression level strongly correlated with tumor differentiation and metastatic risk.^[^ ^146^ ^]^ Targeted therapeutic strategies against this axis show considerable promise. For instance, fucoidan suppresses tumor cell proliferation by downregulating CXCL12 expression, while gene therapy targeting this pathway has exhibited notable antitumor activity in preclinical studies. Moreover, CXCR4 expression patterns aid in distinguishing primary from metastatic LC, providing valuable diagnostic insights.^[^ ^147^, ^148^ ^]^ Despite the incomplete elucidation of its underlying molecular mechanisms, CXCR4 remains one of the most important therapeutic targets in LC.

The folate receptor (FR) is a membrane protein with limited expression in normal tissues but markedly overexpressed on the surface of various malignant tumor cells, including those of LC. By mediating the internalization of its ligand folate, FR participates in purine and pyrimidine synthesis required for cell proliferation, thereby playing a critical role in tumor growth.^[^ ^149^ ^]^ In LC, elevated FR expression is closely associated with LC progression, involving receptor‐mediated endocytosis that enhances nutrient uptake and activates signaling pathways, thus promoting LC cell proliferation and survival. FR has been established as an important molecular target for LC‐specific therapy. Owing to its small molecular weight, low immunogenicity, and high affinity, folate is widely used as a targeting ligand in NMs.^[^ ^150^ ^]^ Studies have shown that folate‐modified NMs efficiently deliver chemotherapeutic agents to LC cells, achieving receptor‐mediated intracellular drug release, thereby enhancing antitumor efficacy while reducing systemic toxicity. Furthermore, folate‐functionalized NMs can co‐deliver siRNA and chemotherapeutic drugs, exerting synergistic effects through RNA interference and chemotherapy to further suppress tumor growth.^[^ ^151^ ^]^ These strategies offer promising directions for precision therapy in LC.

The transferrin receptor (TfR) is a transmembrane glycoprotein overexpressed on the surface of various cancer cells, including LC. By binding with high affinity to iron‐bound transferrin (Tf), TfR mediates the endocytosis of iron ions, thereby playing a central role in maintaining cellular iron homeostasis to support growth.^[^ ^152^ ^]^ In LC, TfR overexpression is closely associated with rapid tumor proliferation. The underlying mechanism involves increased iron uptake to meet the high demand for iron required by rapidly proliferating LC cells. Furthermore, TfR may promote cell survival and proliferation while inhibiting apoptosis by activating downstream signaling pathways such as PI3K/AKT.^[^ ^153^, ^154^ ^]^ Consequently, TfR has become an important molecular target for the targeted therapy of LC. Studies have shown that using Tf or anti‐TfR antibodies as targeting ligands to modify NMs enables the specific delivery of chemotherapeutic drugs to LC.^[^ ^137^ ^]^ These strategies enhance drug enrichment within LC cells via receptor‐mediated endocytosis, thereby improving therapeutic efficacy while reducing systemic toxicity.

## 3 Harnessing the LC Immune Microenvironment for Rational NMs Design

The immunosuppressive TME of LC is not only a barrier to therapy but also a valuable source of therapeutic targets and stimulus‐responsive triggers. Its complex cellular and physicochemical landscape provides a blueprint for designing next‐generation NMs capable of precisely navigating, modulating, and exploiting the TME to achieve effective antitumor immunity.^[^ ^155^ ^]^ This section elaborates on the rational design of NMs based on the specific characteristics of the LC immune microenvironment, enabling them to transcend conventional drug delivery and function as intelligent, integrated systems for immune reprogramming.

The extensive infiltration of immunosuppressive cells, including M2 phenotype TAMs, MDSCs, and Tregs, is a defining feature of the LC immune microenvironment and a major contributor to immunotherapy resistance.^[^ ^156^ ^]^ Rational NMs design enables the selective targeting of these cells to either deplete them or reprogram their immunosuppressive functions. This can be achieved by functionalizing NMs surfaces with ligands that recognize receptors highly expressed on these cell populations. For example, mannose or galactose residues can bind to the mannose receptor on M2 TAMs, while LA facilitates dual targeting through the ASGPR expressed on both LC cells and certain macrophage subsets.^[^ ^157^, ^158^ ^]^ To effectively neutralize distinct immunosuppressive populations, specific inhibitors must be strategically chosen. TAMs are commonly targeted using colony‐stimulating factor 1 receptor (CSF1R) inhibitors, whereas MDSC function can be disrupted by C‐X‐C motif chemokine receptor 2 (CXCR2) antagonists. In addition, STAT3 inhibitors have demonstrated the ability to modulate both cell types simultaneously.^[^ ^159^, ^160^, ^161^ ^]^ NMs encapsulating these inhibitors can be precisely delivered to their intended targets, thereby suppressing immunosuppressive activity within the TME. Moreover, the inherent tropism of certain NMs for the liver and spleen, organs enriched with myeloid cells, can be exploited for passive accumulation. Upon internalization, NMs release their therapeutic payload to repolarize M2 TAMs into the pro‐inflammatory M1 phenotype or induce apoptosis in MDSCs, effectively dismantling the immunosuppressive network of the TME.^[^ ^162^, ^163^ ^]^ This approach transforms NMs from passive carriers into active modulators of the TME, establishing a foundation for robust immune activation.

Beyond cellular targets, the abnormal physiological characteristics of the LC immune microenvironment provide endogenous stimuli for precise spatiotemporal drug release. The mildly acidic pH, elevated activity of specific enzymes such as MMP‐2, MMP‐9, and cathepsins, as well as the redox imbalance characterized by high glutathione (GSH) concentrations, serve as ideal cues for the design of “smart” responsive NMs.^[^ ^164^, ^165^, ^166^, ^167^ ^]^ NMs can be engineered with acid‐labile linkers or pH‐sensitive polymers such as poly(β‐amino ester), which undergo structural transformation or degradation within the acidic tumor interstitium or intracellular endo/lysosomal compartments, thereby triggering drug release.^[^ ^168^ ^]^ Similarly, NMs incorporating enzyme‐cleavable peptides or GSH‐sensitive disulfide linkages ensure that therapeutic agents are predominantly released within the TME, minimizing off‐target toxicity.^[^ ^169^ ^]^ Furthermore, NMs can be designed not only to respond to these pathological conditions but also to actively normalize them. For instance, calcium carbonate‐based NMs can buffer intratumoral acidosis by neutralizing excess protons, thereby alleviating immunosuppression while inducing cytotoxic ionic stress.^[^ ^170^ ^]^ Manganese dioxide‐based NMs, on the other hand, can react with hydrogen peroxide and protons to generate oxygen, mitigating hypoxia and reversing HIF‐1α‐mediated immunosuppression, while simultaneously serving as contrast agents for MRI.^[^ ^171^ ^]^ This dual‐function strategy, which integrates responsive drug release with microenvironment normalization, enables NMs to synergize with their therapeutic payloads and foster a more favorable environment for immune activation.

The most advanced NMs designs aim to establish a self‐sustaining cycle of antitumor immunity by integrating cancer cell elimination with the strong activation of APCs. A central approach involves the targeted induction of ICD. NMs can be loaded with ICD inducers such as chemotherapeutic drugs like doxorubicin (DOX) and oxaliplatin, photosensitizers used in PDT, or agents that amplify oxidative stress, and deliver them specifically to tumor cells.^[^ ^172^, ^173^, ^174^ ^]^ During ICD, cancer cells release DAMPs, including calreticulin, adenosine triphosphate (ATP), and HMGB1, together with tumor‐associated antigens (TAAs). These molecules act as “eat‐me” signals and strong adjuvants that promote DCs activation.^[^ ^175^, ^176^ ^]^ To enhance this process, NMs can be co‐engineered to facilitate antigen capture and presentation by DCs, either through surface modification with DC‐targeting ligands or through the co‐loading of immune adjuvants within the same nanocarrier. This strategy produces an in situ vaccination effect, as the NMs ensure the co‐delivery of both the ICD inducer and the adjuvant to the tumor site, where dying cells supply the antigen source and the adjuvant effectively activates DCs.^[^ ^177^, ^178^ ^]^ The result is efficient cross‐priming of tumor‐specific CTLs, which converts immunologically “cold” tumors into “hot” ones. Furthermore, integrating ICD‐inducing NMs with ICIs within a single formulation can simultaneously overcome immune tolerance, activate effector T cells, and block the PD‐1/PD‐L1 inhibitory pathway, ultimately generating a powerful and coordinated antitumor immune response.

## 4 Targeted Delivery: Overcoming the Barriers in LC Drug Delivery

Multidrug resistance (MDR) frequently occurs during chemotherapy in LC, leading to cancer recurrence and a decline in quality of life. Compared to traditional chemotherapy drugs, targeted therapies provide higher specificity for tumor tissues and greater efficacy in eradicating cancer cells.^[^ ^179^ ^]^ NMs can alter the pharmacokinetics of drugs, enabling either passive or active targeting for immunotherapy, thereby enhancing drug concentrations at LC tumor sites. Moreover, after entering the body, NMs typically accumulate in the liver, depending on their properties. NMs recognize tumor regions through the physicochemical differences between tumor and normal cells.^[^ ^180^ ^]^ Passive targeting, also referred to as the EPR effect, can be optimized by adjusting the size and surface charge of NMs. The EPR effect indicates that molecules or particles of specific sizes accumulate more readily in tumor tissues than in normal tissues, indirectly determining tumor localization. Active targeting involves endowing drugs or their carriers with the ability to actively bind to target markers, determining tumor localization through surface receptors overexpressed on tumor cells. In practice, both targeting strategies are often used simultaneously. Before ligand‐mediated cellular internalization occurs, ligand‐modified NMs initially rely on passive uptake mechanisms in the liver.^[^ ^181^ ^]^

Stimuli‐responsive NMs can dynamically adjust their physical or chemical structure in response to subtle changes in the external environment. These NMs can target the desired site through various stimuli, including external factors such as light, ultrasound, or magnetic fields, and internal factors such as pH and tissue microenvironments. This ability allows for precise and controlled drug release at the tumor site, ensuring the accurate location, quantity, and timing of drug delivery. By doing so, it minimizes potential harm to normal tissues, while maximizing therapeutic efficacy, thus producing highly effective treatment outcomes.^[^ ^182^ ^]^

### 4.1 Passive Targeting: Optimization of NMs Based on the EPR Effect

**Figure 2:**
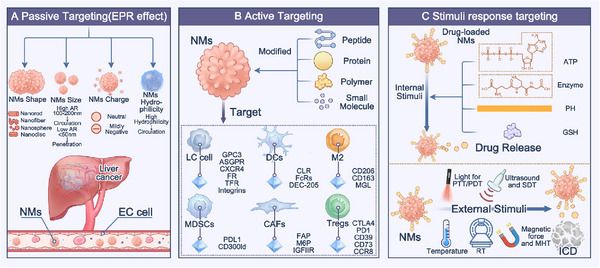
Strategies for overcoming obstacles in drug delivery to LC using NMs: A) NMs achieve passive targeting based on the EPR effect by adjusting their shape, size, Zeta potential, and surface hydrophilicity. B) NMs target LC cells in the immunosuppressive TME through surface modification with various ligands, specifically targeting LC cells, DCs, M2 macrophages, MDSCs, CAFs, and Tregs. C) NMs respond to various endogenous or exogenous stimuli to trigger targeted drug release, while inducing ICD.

In LC treatment, the design of NMs based on the EPR effect is a key strategy for achieving passive targeting (**Figure** **2**A). The newly formed vasculature in solid tumors is abundant, with wider endothelial gaps and relatively unstable structures, coupled with inadequate lymphatic drainage, creating favorable conditions for NMs accumulation.^[^ ^183^ ^]^ Unlike most other tissues, the liver lacks an impermeable basement membrane. Consequently, in the absence of interfering mechanisms such as aggregation or protein binding, most NMs exhibit rapid passive hepatic accumulation after systemic administration.^[^ ^184^ ^]^ Passive targeting of hepatocytes is primarily determined by the diameter of the hepatic sinusoid fenestrae, which can select for sinusoidal endothelial cells (SECs) and KCs on one side (>100 nm) or hepatocytes and hepatic stellate cells (HSCs) on the other (<100 nm). In addition to these mechanisms, it is hypothesized that in certain cases, forced extrusion, potentially due to transient interactions with SECs, may allow larger (<400 nm) deformable nanocarriers to extravasate into the perisinusoidal space.^[^ ^185^ ^]^ Early passive targeting approaches primarily targeted non‐parenchymal cells located within the sinusoids (i.e., KCs) due to their intravascular location. KCs are well‐recognized for their intrinsic role in particle endocytosis via scavenger receptors.^[^ ^186^ ^]^ Besides KCs, SECs are also involved in the clearance of negatively charged liposomes through scavenger receptor‐mediated stabilization.^[^ ^187^ ^]^ Studies have shown that the shape of NMs significantly affects their circulation lifespan, flow characteristics, tumor accumulation, cellular uptake, and tumor tissue penetration. Generally, short nanorods and spherical NMs with a diameter of 100–200 nm exhibit favorable circulation behavior. Nanorods and nanofibers with a high aspect ratio (AR) tend to accumulate more in tumor sites, whereas small nanospheres or nanodiscs (<50 nm) and short nanorods with a low AR are more conducive to tumor tissue penetration. In addition, the AR and surface hydrophilicity of NMs regulate their interactions with cells, cellular uptake efficiency, and drug accumulation at tumor sites.^[^ ^183^ ^]^ A neutral or mildly negative charge minimizes nonspecific plasma protein adsorption, thereby prolonging circulation time. Besides particle properties, the route of injection also influences hepatic uptake and intrahepatic distribution. Intratumoral and intra‐arterial injections have been used to deliver small‐molecule anticancer drugs like DOX to LC.^[^ ^188^, ^189^ ^]^ Local injection is one method to circumvent the drawback of low intracellular uptake associated with intravenous administration; however, it is characterized by low patient compliance and is difficult to implement. Therefore, these techniques remain a niche application, and most research focuses on improving hepatic targeting of NMs via standard routes of administration such as intravenous injection.^[^ ^190^ ^]^ Polyethylene glycol (PEG) modification, a well‐established strategy, reduces clearance by the mononuclear phagocyte system (MPS) as well as enables active targeting and synergistic enhancement of the EPR effect by incorporating functional molecules such as folic acid (FA) or hyaluronic acid (HA).^[^ ^191^ ^]^ For instance, Dong et al. developed a drug delivery system based on PLGA‐PEG NMs, which effectively targeted HECTD2 in vivo, significantly enhancing the targeting efficiency in LC cells.^[^ ^192^ ^]^

However, clinical translation remains challenging. Tumor heterogeneity leads to variability in the EPR effect. Factors such as vascular maturity, interstitial fluid pressure, and ECM density in LC significantly influence NMs distribution.^[^ ^193^ ^]^ In the second place, the discovery of the basement membrane barrier has challenged conventional understanding. The dense basement membrane on the abluminal side of tumor endothelial cells hinders further NMs penetration, causing their retention in vascular “pools.” Enzymatic degradation or inflammation‐induced neutrophil migration can temporarily open this barrier to enhance penetration.^[^ ^194^, ^195^ ^]^ In addition, the dense collagen network and tumor metabolic microenvironment may further restrict NMs penetration, necessitating vascular disruption strategies, such as STING pathway activation, for precise modulation.^[^ ^196^ ^]^ Future research should focus on personalized therapy by leveraging genomics and multimodal imaging to optimize NMs design.

### 4.2 Active Targeting: Modification with LC‐Specific Ligands

The primary objective of active targeting is to achieve precise identification and binding of targets by endowing drugs or their carriers with specific binding capabilities. Through the surface functionalization of NMs, ligands are used to modify the surfaces of NMs with targeting agents such as peptides, proteins, small molecules, and polymers.^[^ ^197^ ^]^ These modifications enable the specific recognition of receptors or cell markers overexpressed on the surfaces of LC cells or immune cells, as shown in **Table** **1**. This active targeting technology allows immunotherapeutic drugs to be selectively delivered to target cells, thereby significantly enhancing therapeutic efficacy and reducing systemic toxicity during drug administration, such as in immunotherapy.^[^ ^198^, ^199^ ^]^

**Table 1:** Current common targets for NM targeting and their mechanisms for reversing the immunosuppressive TME.

Targeted cell	Targeting receptor	NMs	Specific ligand	Mechanisms	Refs.
LC cell	GPC3	GV‐Lipo/SF/DT	Anti‐GPC3 mAb	Targets GPC3, disrupts clusters via Ca^2^⁺, inhibits neutrophil adhesion, and induces cytotoxicity.	[200]
		CR‐PEG‐GBP	GPC3‐binding peptide	Targets GPC3, self‐assembles at low pH, enabling imaging‐guided PTT and SDT.	[201]
	ASGPR	NO‐DOX@PDA‐TPGS‐Gal	Galactose	Targets ASGPR via galactose, pH‐responsive dissociation enables chemo‐photothermal therapy with MDR reversal.	[202]
		Dox/Pac Lac‐BSA NMs	Lactose	Lactose‐ASGPR targeting enables pH‐responsive dual‐drug synergistic therapy against LC	[203]
	CXCR4	ADOPSor NMs	AMD3100	Targets CXCR4, remodels TME, enabling anti‐angiogenesis and drug resistance reversal	[204]
		CTCE‐p53 NMs	CTCE‐9908 peptide	Delivers p53 mRNA via CXCR4 targeting, synergizes with anti‐PD‐1 to reprogram TME and overcome ICB resistance.​	[205]
	FR	FA‐CS/mIP‐10 NMs	Folate	Recruits CXCR3+ CTLs via IP‐10, synergizes with DC vaccine to remodel immunosuppressive TME.	[206]
		FA‐modified multifunctional NMs	FA‐PEG‐DSPE	Targets FR, reverses chemoresistance, and induces apoptosis in LC	[207]
	TfR1	Tf‐LP‐CA	Tf	Targets TfR, enhances tumor accumulation; Carnosic Acid induces mitochondrial apoptosis.	[208]
		Tf@IR820‐DHA	Genetically engineered Tf	Targets TfR1, induces oxidative stress and ICD, and synergizes with anti‐PD‐L1 for systemic immunity.	[209]
	Integrins(αvβ3/αvβ5)	RGD‐PEG‐CS‐SA	RGD peptide (GSSSGRGDSPA)	RGD targets integrin αvβ3/5, enhances DOX uptake and cytotoxicity via endocytosis.	[210]
	Integrins(αvβ3)	Au@PDA‐RGD	SH‐PEG‐RGD	RGD targets integrin αvβ3, induces hyperthermia via NIR, causing autophagic cell death.	[211]
DCs	CLR	MN‐PLGA NMs	Mannose	Mannose targets CLR on DCs, enhances uptake and promotes maturation (CD40/CD86↑).	[212]
	FcγRII/III	IgG‐coated liposomes	IgG1/IgG2a	Multivalent IgG targets surface Ig or FcγR, enhances antigen uptake and presentation.	[213]
	DEC‐205	anti‐DEC‐205‐EUPS‐PLGA NMs	Anti‐DEC‐205 mAb	Anti‐DEC‐205 mAb directs NMs to DEC‐205 receptors on DCs, enhancing antigen uptake.	[214]
		hr‐8‐PLGA@Ag/CpG	hr‐8 peptide	Peptide hr‐8 targets DEC‐205 on DCs, promotes maturation, and antigen cross‐presentation.	[215]
M2 macrophages	CD206	ManNP	α‐Mannose	Mannosylated NPs target CD206 on M2 macrophages, enhancing siRNA delivery.	[216]
	CD163	PEG‐LNP(Cal)	αhCD163/αmCD163	CD163‐targeted LNPs enter macrophages, inhibit NF‐κB, downregulate pro‐inflammatory cytokines, and upregulate IL‐10.	[217]
		CD163‐GNPs	αCD163	CD163‐GNPs preferentially target M2 macrophages, synergize with radiotherapy to reduce tumor growth.	[218]
	MGL	GDO	Galactose residues	Galactose targets Mgl on TAMs, anti‐IL‐10 ODNs block immunosuppression, and repolarize TAMs to pro‐inflammatory phenotype.	[219]
MDSCs	PD‐L1	LPIP	αPD‐L1	LPIP releases IPI549 and αPD‐L1 upon mild hyperthermia, blocks PI3Kγ and PD‐L1 pathways.	[220]
CAFs	FAP	GHSO@PTX	dipeptide Z‐glycine‐proline	ZGP peptides target FAP on CAFs, pH/ROS‐responsive PTX release kills LC cells and CAFs, and reverses immunosuppressive TME.	[221]
CAFs/HSCs	M6P/IGFIIR	M6P_28_‐HSA	M6P_28_	M6P_28_‐HSA targets M6P/IGFII receptor on HSCs, enables lysosomal delivery of antifibrotic drugs.	[222]

#### 4.2.1 Receptors for Targeting LC Cells

The surface of NMs can be modified with ligands that have high affinity for receptors to achieve targeted delivery. After passive delivery to the liver, these ligands can bind to receptors on specific cell types and exert their corresponding effects. LC cells express various receptors that may facilitate drug targeting. This section summarizes the major LC cell receptors currently being studied.^[^ ^223^ ^]^

The surface of NMs can be modified with ligands that have high affinity for receptors to achieve targeted delivery. After passive delivery to the liver, these ligands can bind to receptors on specific cell types and exert their corresponding effects. LC cells express various receptors that may facilitate drug targeting. This section summarizes the major LC cell receptors currently being studied.

GPC3 is a cell membrane‐anchored oncofetal proteoglycan primarily expressed in the fetal liver but almost absent in the liver of healthy adults. However, in patients with LC, GPC3 is overexpressed at both the gene and protein levels, and its expression is associated with poor prognosis. Mechanistic studies indicate that GPC3 plays a critical role in LC progression by binding to molecules such as Wnt signaling proteins and growth factors. Consequently, GPC3 has been widely recognized as a potential diagnostic and therapeutic target for LC.^[^ ^138^ ^]^ One study evaluated liposomes coated with anti‐GPC3 antibodies (GV‐Lipo/SF/DT) for the delivery of Sor in LC treatment. These liposomes were able to target circulating tumor cells, dissociate tumor cell clusters, prevent the formation of circulating tumor cell‐neutrophil clusters, and inhibit tumor metastasis. In the H22 tumor mouse model, the liposomes showed significant antitumor effects.^[^ ^200^ ^]^ In addition, GPC3‐targeted NMs are an effective strategy for tumor imaging. Li et al. developed a pH‐sensitive self‐assembled GPC3‐binding peptide (GBP) dye CR‐PEG‐GBP as an intelligent nanoprobes for NIR imaging, photoacoustic (PA) imaging‐guided PTT, and sonodynamic therapy (SDT) in LC. Experimental results indicated that this small‐molecule assembled nanoprobe exhibited favorable properties, such as responding to changes in pH (from normal tissue pH ≈7.4 to TME pH ≈6.5), and aggregated from small NMs (less than 20 nm at pH ≈7.4) into larger NMs (greater than 160 nm at pH ≈6.5, and greater than 510 nm at pH ≈5.5), thus enhancing imaging and therapeutic effects. Because CR‐PEG‐GBP can self‐assemble in situ in the acidic TME, it shows high tumor accumulation and prolonged tumor retention time, while being cleared from normal tissues and showing significant safety.^[^ ^201^ ^]^

ASGPR is predominantly expressed on hepatocytes, with minimal expression in extrahepatic cells. Extensive studies have confirmed its presence in LC, making it an attractive target for receptor‐mediated drug delivery, while minimizing toxicity concerns. ASGPR facilitates endocytosis through clathrin‐mediated pathways and exhibits a high affinity for carbohydrates, particularly galactose, N‐acetylgalactosamine, and glucose.^[^ ^142^ ^]^ DOX is a primary chemotherapeutic agent for LC treatment. However, its long‐term use is limited by severe side effects, such as cardiomyopathy and congestive heart failure. In addition, prolonged administration of DOX and other anthracycline‐based chemotherapeutic agents can induce drug resistance in LC, which poses a major obstacle to successful treatment.^[^ ^224^ ^]^ The development of targeted drug carriers that enhance drug delivery efficiency remains a key focus in LC research. Du et al. developed a novel NM based drug delivery system with a core‐shell structure composed of D‐alpha‐tocopheryl polyethylene glycol 1000 succinate (TPGS)‐galactose (Gal)/polydopamine (PDA). These NMs were loaded with thermosensitive DOX and the nitric oxide (NO) donor N,N’‐disec‐butyl‐N,N’‐di‐nitroso‐1,4‐phenylenediamine (BNN) to release NO (DOX@PDA‐TPGS‐Gal). The specific binding of Gal to ASGPR, combined with the pH‐sensitive degradation of NMs, ensured targeted transport to hepatocytes and controlled DOX release in LC cells. Consequently, the combination therapy of NO‐DOX@PDA‐TPGS‐Gal exhibited potent anticancer activity against drug‐resistant LC cells in both in vitro and in vivo studies, significantly prolonging the survival of tumor‐bearing mice. This NM‐based strategy provides a promising approach for highly targeted LC therapy and overcoming tumor drug resistance.^[^ ^202^ ^]^

**Figure 3:**
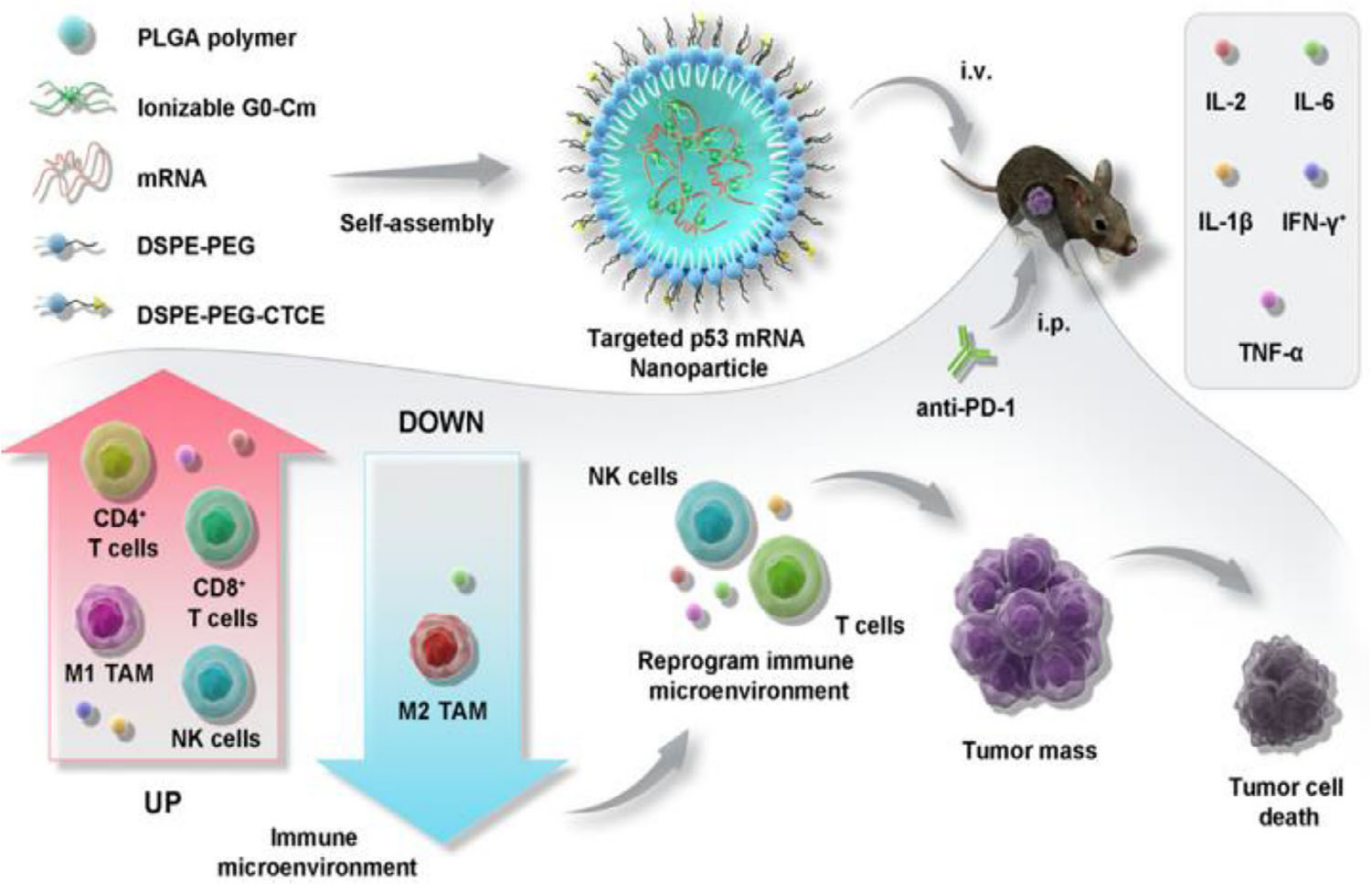
Schematic of CXCR4‐targeted p53 mRNA NMs and the combinatorial strategy of using anti‐PD‐1 therapy to reprogram the immunosuppressive TME for effective treatment of p53‐deficient LC. Reproduced (Adapted) with permission.^[^ ^205^ ^]^ Copyright 2022, Springer Nature.

CXCR4 has garnered significant attention over the past decade owing to its upregulation in various cancer types, including LC.^[^ ^146^ ^]^ CXCR4‐associated signaling pathways, such as the CXCR4/stromal‐derived factor 1α (SDF1α) pathway, promote tumor growth and metastasis through multiple mechanisms. Gao et al. formulated Sor within CXCR4‐targeted lipid‐coated poly(lactic‐co‐glycolic acid) (PLGA) NMs, modifying the NMs with the CXCR4 antagonist AMD3100 to enhance systemic Sor delivery to LC and improve tumor sensitivity to treatment. This approach achieved cytotoxic and anti‐angiogenic effects both in vitro and in vivo.^[^ ^204^ ^]^ Targeting CXCR4 with NMs provides an effective strategy to counteract the immunosuppressive TME in LC. A study developed and optimized a CXCR4‐targeted mRNA NM platform to efficiently induce p53 expression in an LC model. Combining CXCR4‐targeted p53 mRNA NMs with PD‐1 therapy successfully reprogrammed cellular and molecular components within the immunosuppressive TME. Compared to monotherapies with either anti‐PD‐1 treatment or therapeutic p53 expression alone, this combination therapy significantly enhanced antitumor efficacy (**Figure** **3**).^[^ ^205^ ^]^

FR is a secretory protein anchored to the membrane through glycosyl‐phosphatidylinositol linkage or exists in a soluble form. The distribution of FRs is highly limited in normal tissues but is overexpressed in malignant cells. Owing to its small size, lack of immunogenicity, non‐toxic nature, low cost, and ease of conjugation with NMs, folate has been widely used as a homing ligand in cancer‐targeted therapies.^[^ ^225^, ^226^ ^]^ To enhance the therapeutic efficacy of DC and tumor cell fusion vaccines, Hu et al. used folic acid (FA)‐modified CS NMs, a non‐viral carrier capable of targeting tumor cells with high FA receptor expression. FA‐CS NMs were used as biological carriers for the murine interferon‐inducible protein‐10 (mIP‐10) gene expression plasmid, a potent chemical chemoattractant for CTLs. The combination of FA‐CS/mIP‐10 NMs and DC/anti‐LC fusion vaccines significantly reduced the MDSCs in the spleen, local tumors, and bone marrow of mice, while increasing tumor‐specific IFN‐γ responses, thereby enhancing the therapeutic efficacy of the original fusion vaccine and alleviating the immunosuppressive TME.^[^ ^206^ ^]^ In addition, the therapeutic effect on LC can be enhanced by designing combination drug delivery systems conjugated with FA. It has been reported that FA‐modified multifunctional NMs, loaded with docetaxel and iSur‐pDNA (survivin), provide a safer and more effective strategy for treating advanced LC.^[^ ^207^ ^]^

TfR, also known as cluster of differentiation 71 (CD71), is a type II transmembrane glycoprotein that binds Tf and plays a crucial role in cellular iron uptake through interaction with iron‐bound Tf.^[^ ^227^ ^]^ The binding of the ligand to the receptor initiates endocytosis, resulting in the internalization of the iron‐bound transferrin‐TfR complex. Iron is essential for various cellular processes, including DNA synthesis and cell proliferation. Owing to its central role in cancer cell pathology, malignant cells often overexpress TfR, and this increased expression may be associated with poor prognosis in various cancer types.^[^ ^228^ ^]^ The elevated expression of TfR on malignant cells, along with its extracellular accessibility, internalization ability, and central role in cancer cell pathology, makes this receptor an attractive target for targeted delivery.^[^ ^227^ ^]^ Several studies have revealed that TfR ligand‐modified NMs promote the accumulation of the modified NMs in LC cells, enhancing drug efficacy. Therefore, TfR ligand‐modified NMs hold promise as targeted therapies for LC.^[^ ^208^, ^229^, ^230^ ^]^ Bai et al. developed a gene‐engineered transferrin‐expressing cell membrane NM encapsulating IR 820‐dihydroartemisinin nanodrug (Tf@IR820‐DHA) to enhance anti‐PD‐L1‐mediated immune checkpoint blockade (ICB) through synergistic triple‐stimulation activation of oxidative stress‐related ICD. They showed that these NMs possess targeted tumor specificity and Fe (III) loading characteristics, inducing high levels of targeted ICD through oxidative stress activation. This represents a promising approach for cancer nanoimmunotherapy.^[^ ^209^ ^]^

Integrins are a family of heterodimeric transmembrane glycoproteins composed of α and β subunits. To date, 24 different integrin subtypes have been identified, nearly half of which interact with various ECM proteins through the tripeptide motif Arg‐Gly‐Asp (RGD). These integrins play critical roles in cell signaling, cell‐cell adhesion, cell‐matrix adhesion, and apoptosis.^[^ ^231^ ^]^ Certain integrin subtypes are overexpressed on cancer cells or endothelial cells of newly formed blood vessels, where they play pivotal roles in tumor angiogenesis and metastasis.^[^ ^225^ ^]^ Currently, the most common integrin subtypes targeted by NMs are αvβ3 and α6β1. In vitro and in vivo studies have reported that RGD‐modified NMs hold great potential as drug carriers for active‐targeted therapy of LC. Compared to free DOX and DOX‐loaded NMs lacking the RGD ligand, DOX‐loaded RGD‐modified micelles exhibit enhanced cytotoxicity against BEL‐7402 cells.^[^ ^210^ ^]^ Moreover, improved antitumor efficacy was observed in an orthotopic mouse hepatoma model.^[^ ^232^ ^]^ Yang et al. developed RGD peptide‐conjugated PDA‐coated gold NMs (Au@PDA‐RGD NMs) for targeted LC therapy. Their findings indicate that Au@PDA‐RGD NMs effectively target and treat HepG2 cells that overexpress the αvβ3 integrin receptor in vitro.^[^ ^211^ ^]^

#### 4.2.2 Receptors for Targeting DC Cells

In LC therapy, the design of NMs targeting DCs is emerging as a key strategy to enhance antitumor immunity. These NMs can precisely deliver antigens or immunomodulators by specifically targeting DC surface markers or microenvironmental regulatory molecules, thereby activating DC‐mediated T cell responses. This section summarizes the major DC cell receptors currently under investigation.^[^ ^233^ ^]^

The mannose receptor is a C‐type lectin receptor (CLR) expressed on DCs and macrophages. CLRs are calcium‐dependent receptors, and their carbohydrate recognition domains share structural homology.^[^ ^234^ ^]^ In addition to immature DCs, monocytes, macrophages, and subsets of endothelial cells also express this receptor. Targeting this receptor by simply conjugating cancer antigens with mannose complexes, without using a specialized delivery system, has achieved significant success in both preclinical and clinical studies.^[^ ^235^ ^]^ Hamdy et al. chemically conjugated mannan with PLGA NMs and loaded them with a model antigen, ovalbumin (OVA), to develop a system capable of targeting DCs and enhancing antigen‐specific CD4^+^ and CD8^+^ T cell responses.^[^ ^212^ ^]^

Fc receptors (FcRs) bind to the constant domain of antibodies, serving as a critical link between humoral and cellular immune responses. Each immunoglobulin class has a distinct FcR.^[^ ^236^ ^]^ Leserman and Machy first used antibodies targeting these receptors for research purposes. In their study, IgG‐coated liposomes specific to FcγRII and FcγRIII were used. These targeted liposomes were efficiently internalized by DCs through FcRs and effectively presented to T cells.^[^ ^213^ ^]^ Park et al. fused apolipoproteins to the Fc domain of targeting antibodies, enabling their spontaneous display on the surface of mRNA‐lipid nanoparticles (NPs) (mRNA@LNPs). This approach significantly enhanced the specificity and delivery efficiency of mRNA@LNPs, while reducing their cytotoxicity.^[^ ^237^ ^]^

**Figure 4:**
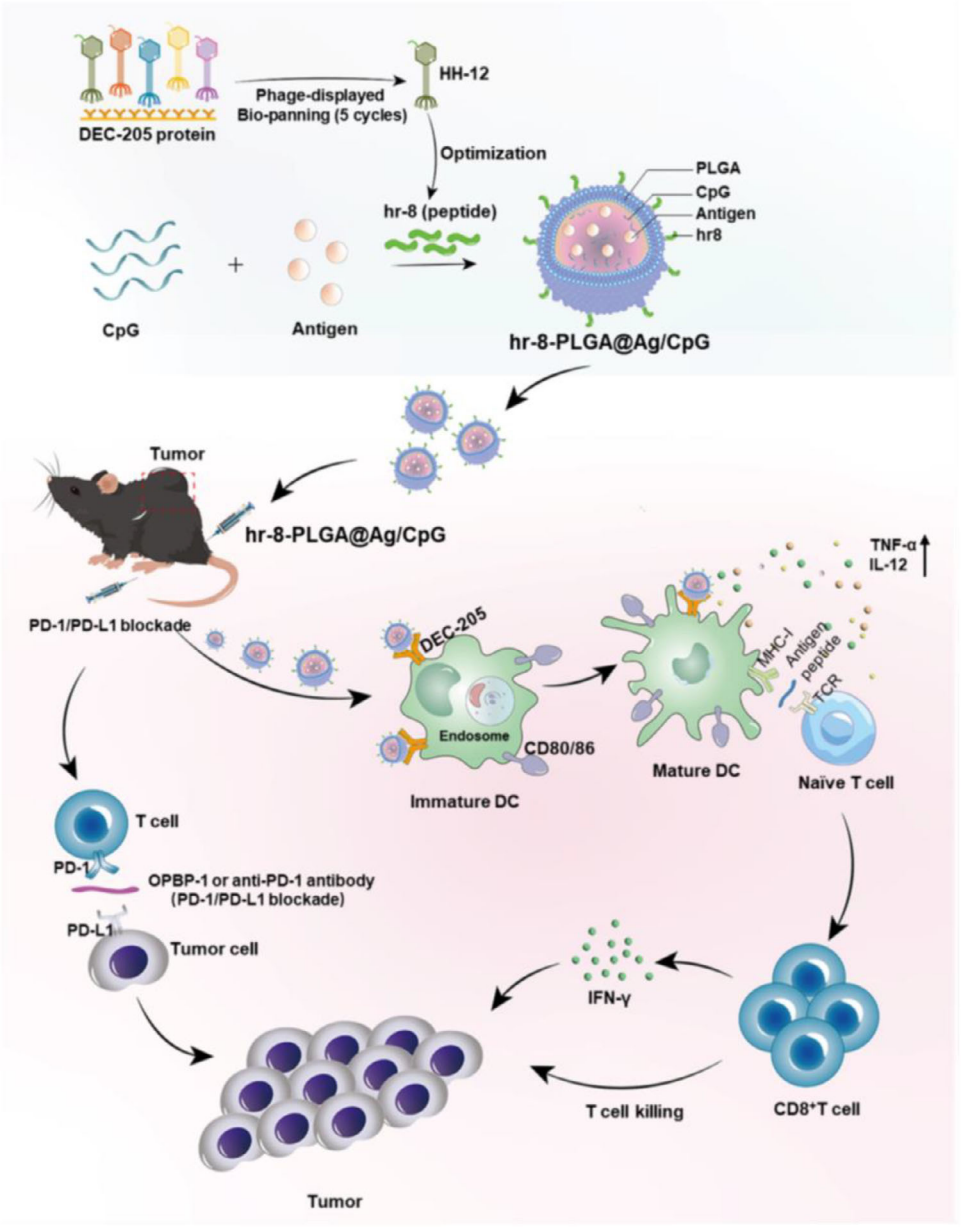
Schematic of the HR‐8‐PLGA@Ag/CpG NMs targeting cDC1 endocytic receptor DEC‐205 for cancer immunotherapy. Reproduced (Adapted) with permission.^[^ ^215^ ^]^ Copyright 2024, Elsevier.

DEC‐205 is a transmembrane protein that binds to carbohydrates and mediates endocytosis.^[^ ^238^ ^]^ Studies have shown that NMs targeting the DEC‐205 receptor induce antigen‐specific protective immune responses. When the delivery system carries both antigens and immunomodulators, it enhances therapeutic efficacy and elicits strong humoral and cellular immune responses. Feng et al. conjugated *Eucommia ulmoides Oliv*. polysaccharides to PLGA NMs and further functionalized them with anti‐CD205 monoclonal antibodies, generating PLGA NMs specifically targeting the DEC‐205 receptor (anti‐DEC‐205‐EUPS‐PLGA NMs). Their findings revealed that these targeted adjuvants promoted dendritic cell maturation and induced both humoral and cellular immune activation, providing new insights into vaccine adjuvant development.^[^ ^214^ ^]^ Zheng et al. identified peptides that target the endocytic receptor DEC‐205, primarily expressed on cDC1 cells. They optimized a hydrolysis‐resistant peptide, hr‐8, and conjugated it to PLGA NMs loaded with antigens and CpG adjuvants, creating a dendritic cell‐targeting nanovaccine. The resulting hr‐8‐PLGA@Ag/CpG nanovaccine enhanced dendritic cell maturation and improved antigen cross‐presentation. This study introduced an innovative approach to dendritic cell‐targeting nanovaccines using DEC‐205‐binding peptides, contributing to cancer vaccine development (**Figure** **4**).^[^ ^215^ ^]^

#### 4.2.3 Receptors for Targeting M2 Macrophages

In the TME of LC, M2 phenotype TAMs promote tumor progression, metastasis, and drug resistance by secreting immunosuppressive factors such as IL‐10 and TGF‐β, which facilitate angiogenesis and matrix remodeling.^[^ ^239^ ^]^ The design of NMs targeting M2 phenotype TAMs has emerged as a crucial strategy for reshaping the immunosuppressive microenvironment. This approach relies on the specific recognition of M2 cell biomarkers and the precise delivery of drugs, genes, or immunomodulators to achieve targeted intervention.

CD206 is a mannose receptor belonging to the calcium‐dependent type I transmembrane protein family. It is widely expressed in macrophages within lymph nodes, thymic cortex, and splenic red pulp and serves as the most specific marker of M2 phenotype TAMs.^[^ ^240^ ^]^ CD206 recognizes various glycomolecules on the cell walls or surfaces of pathogens and participates in immune defense regulation through receptor‐mediated phagocytosis and endocytosis.^[^ ^241^ ^]^ Kaps et al. designed mannose residue‐coated nanohydrogel particles (ManNP), which exhibited significantly enhanced siRNA delivery to M2 polarized macrophages compared to their non‐targeted NP (NonNP) in vitro. In vivo NIR imaging revealed that siRNA‐ManNP exhibited a high degree of colocalization with CD206^+^ M2 macrophages at the cellular level, whereas NonNP showed minimal colocalization and was predominantly taken up non‐specifically by other hepatic cells.^[^ ^216^ ^]^ These findings confirm that targeting CD206 is an effective strategy for drug delivery to reprogram M2 phenotype TAMs into the M1 phenotype and mediate immunotherapy.

CD163 is a cysteine‐rich class B scavenger receptor with a molecular weight of ≈130 kDa and belongs to the single‐pass transmembrane glycoprotein family.^[^ ^242^ ^]^ As a key member of the scavenger receptor superfamily, CD163 is primarily expressed on the surface of monocytes and macrophages and is considered a highly specific marker of M2 phenotype TAM.^[^ ^243^ ^]^In addition to its role as an anti‐inflammatory immunomodulator, CD163 plays a critical role in tumor proliferation and metastasis as a member of the TAM family. Studies have shown a strong correlation between CD163 and various malignancies, including LC, bladder cancer, lung cancer, and colorectal cancer.^[^ ^244^, ^245^, ^246^, ^247^ ^]^ Aisha et al. designed and developed NIR calcitriol‐loaded polyethylene glycol‐modified lipid NPs (PEG‐LNP(Cal)) using a microfluidic mixing technique. These modified LNPs targeted the M2‐specific endocytic receptor CD163. In vivo pharmacodynamic and biodistribution studies revealed that targeting CD163 significantly enhanced drug delivery efficiency.^[^ ^217^ ^]^ In another study, CD163 antibody‐conjugated silica‐coated gold NPs (CD163‐GNPs) were synthesized, resulting in improved uptake by M2 macrophages. Immunostaining analysis revealed that M2 macrophages internalized more CD163‐GNPs compared to MO or M1 macrophages.^[^ ^218^ ^]^

Macrophage galactose‐type lectin (MGL) is a CLR primarily expressed by myeloid cells, such as macrophages and immature dendritic cells. MGL exhibits immunosuppressive properties, contributing to immune homeostasis.^[^ ^248^ ^]^ Consequently, tumors and certain pathogens exploit MGL to evade immune surveillance by inducing an immunosuppressive microenvironment. The M2 phenotype TAMs leads to high MGL expression, whereas macrophages in normal tissues, such as peritoneal macrophages, exhibit lower MGL levels. This differential expression enables selective targeting through MGL.^[^ ^249^ ^]^ Huang et al. developed a nanocomplex (GDO) by electrostatically binding cationic dextran (C‐Dextran) with negatively charged oligonucleotides (ODN). To enhance MGL recognition, galactose residues were introduced onto dextran through LA conjugation. This modification facilitated the targeted delivery of nucleic acid therapeutics to M2 phenotype TAMs, reprogramming their immunosuppressive phenotype and ultimately inhibiting tumor growth and angiogenesis.^[^ ^219^ ^]^

#### 4.2.4 Receptors for Targeting Other Immunosuppressive Cells

PD‐L1 is an immune checkpoint molecule that typically binds to PD‐1, inhibiting T cell activity and thereby modulating immune responses. Under normal conditions, PD‐L1 expression helps maintain immune tolerance and prevent autoimmune reactions.^[^ ^250^ ^]^ Generally, PD‐L1 expression is low in MDSCs, but it is significantly induced in tumor or inflammatory microenvironments, leading to immune suppression and escape. Targeting PD‐L1, such as through the use of αPD‐L1 antibodies, can block this compensatory mechanism and restore anti‐tumor immune responses.^[^ ^251^ ^]^ Tang et al. developed a size‐adjustable nano‐micro hybrid liposome (LPIP) that responds to the transition zone temperature of incomplete radiofrequency ablation (iRFA) to co‐deliver MDSC inhibitors (IPI549) and anti‐PD‐L1 antibodies. Compared to untargeted iRFA treatment, LPIP releases drugs in the mild thermal environment induced by iRFA, selectively suppressing MDSCs and blocking their compensatory upregulation of PD‐L1. In vivo bioluminescence imaging, flow cytometry, and immunohistochemistry (IHC) revealed that LPIP combined with iRFA significantly reduced MDSC infiltration, promoted M1 macrophage polarization, and enhanced cytotoxic T cell activity, effectively inhibiting residual tumor recurrence. This study confirms that combined targeting of MDSCs and their PD‐L1‐mediated immune escape is a key strategy to enhance anti‐tumor immune responses.^[^ ^220^ ^]^

Targeted immune approaches against MDSCs have gained significant research attention. CD300ld is a recently identified immune inhibitory receptor that is highly expressed on polymorphonuclear MDSCs (PMN‐MDSCs) and promotes tumor immune escape by regulating neutrophil recruitment and function. In tumor‐bearing mouse models, CD300ld knockout significantly reduces PMN‐MDSC infiltration into tumors, enhances the activation and migration of CD8^+^ T cells, and reshapes the immunosuppressive microenvironment into an antitumor state. Therefore, antibodies targeting CD300ld may provide a novel strategy for constructing NMs targeting MDSCs.^[^ ^252^ ^]^

Fibroblast activation protein (FAP) is considered a specific marker of CAFs. It is a membrane‐bound serine dipeptidyl peptidase composed of 760 amino acids.^[^ ^253^ ^]^ FAP is nearly absent in healthy tissues but is upregulated 14–18‐fold in cirrhosis and LC compared to healthy liver tissue.^[^ ^254^ ^]^ FAP supports tumor growth through multiple mechanisms, with one consistent finding being its role in promoting tumor cell proliferation, migration, and invasion, all of which contribute to tumor progression. The high specificity of FAP expression on the surface of CAFs makes it a key marker for distinguishing CAFs from normal fibroblasts.^[^ ^194^ ^]^ Guo et al. designed a targeted NMs modified with the dipeptide Z‐glycine‐proline (ZGP) to specifically bind FAP on the surface of CAFs. In vitro experiments revealed that CAFs exhibited a 2.1‐fold increase in uptake efficiency of ZGP‐modified NMs compared to unmodified ones, confirming that targeting CAFs can effectively overcome stromal barriers, enhance deep drug penetration, and improve therapeutic efficacy.^[^ ^221^ ^]^

Because of shared immune signaling pathways in liver fibrosis and LC, as well as similarities between activated HSCs and CAFs, regulatory targets for NMs used in LC treatment may also serve as promising candidates for selectively targeting CAFs in future research.^[^ ^255^ ^]^ Mannose‐6‐phosphate/insulin‐like growth factor II receptor (M6P/IGFIIR) is a transmembrane glycoprotein with a large extracellular domain. This receptor plays a crucial role in the removal and degradation of extracellular proteins through endocytosis and participates in G protein‐coupled receptor (GPCR) signaling. Several IGFIIR ligands, with or without M6P modifications, have been identified, including renin, granzyme B, thyroglobulin, latent TGF‐β1, proliferin, and the urokinase‐type plasminogen activator receptor.^[^ ^256^ ^]^ IGFIIR is upregulated in activated HSCs and is strongly expressed on HSCs/CAFs during LC progression.^[^ ^257^ ^]^ Beljaars et al. designed a human serum albumin (HSA) carrier modified with 28 M6P groups (M6P_28_‐HSA), which exhibited specific binding to activated HSCs compared to unmodified HSA. Through in vitro receptor competition assays and lysosomal pathway inhibition experiments, M6P28‐HSA was confirmed to be efficiently internalized by activated HSCs through M6P/IGFIIR‐mediated endocytosis, whereas quiescent HSCs showed minimal binding. This study showed that the M6P/IGFIIR‐targeting system enables intracellular drug delivery to activated HSCs, providing a precise strategy for the treatment of liver fibrosis and LC.^[^ ^222^ ^]^

Tregs are key mediators of immunosuppression in the TME, making their surface molecular targets crucial for advancing tumor immunotherapy. In addition to classical immune checkpoints such as CTLA‐4 and PD‐1, as well as metabolism‐related molecules such as CD39/CD73, the chemokine receptor CCR8 has emerged as a highly promising therapeutic target because of its specific expression in tumor‐infiltrating Tregs (TI‐Tregs).^[^ ^258^, ^259^ ^]^

CCR8, a GPCR, binds to its ligand CCL1, which is secreted by CAFs and TAMs. This interaction activates the STAT3 signaling pathway, upregulating immunosuppressive molecules such as FOXP3, CD39, and IL‐10, while promoting the recruitment of peripheral Tregs to tumor sites and facilitating the conversion of CD4⁺ T cells into Tregs. These processes collectively enhance tumor immune evasion.^[^ ^260^ ^]^ Notably, CCR8 is highly expressed in up to 80% of TI‐Tregs in solid tumors but is nearly absent in Tregs from normal tissues. This tumor‐specific expression enables precise targeting of TI‐Tregs without disrupting physiological immune homeostasis.^[^ ^202^ ^]^ Currently, antibody‐based therapies targeting CCR8 have entered clinical translation. The core strategy involves selective depletion of TI‐Tregs through ADCC. For instance, BMS‐986340 enhances ADCC activity through afucosylation and, when combined with PD‐1 inhibitors, significantly improves objective response rates (ORR) in advanced solid tumors. However, studies incorporating CCR8 into NMs‐based therapies remain limited. Because of its critical role in Treg‐mediated immune regulation, CCR8 represents a highly promising target for NMs‐based immunomodulation.^[^ ^261^, ^262^ ^]^

In summary, the advantages of active targeting are primarily manifested in the following aspects:

1. Enhanced tumor cell‐specific uptake to overcome extracellular barriers: passive targeting primarily relies on the EPR effect, leading to the accumulation of NMs within the tumor interstitium. However, this represents merely an extracellular enrichment. To exert their therapeutic effects, NMs must be efficiently internalized by the LC cells themselves.^[^ ^263^ ^]^ Active targeting strategies significantly enhance the binding and internalization of NMs to LC cells, thereby enabling more precise intracellular drug delivery and reducing drug loss or degradation in the ECM.
2. Improved tumor penetration depth: Owing to tumor heterogeneity and elevated interstitial fluid pressure, the penetration depth of NMs within the tumor mass, when relying solely on passive diffusion, is often limited, potentially resulting in therapeutic blind spots. The active targeting mechanism functions as a “molecular guidance” system, directing NMs towards tumor regions expressing the target antigens, including the core areas, thereby promoting more uniform distribution and enhancing tumor cell killing efficacy.^[^ ^264^, ^265^ ^]^
3. Potential reduction of side effects associated with MPS sequestration: Although MPS uptake contributes to the hepatic accumulation of NMs, a significant portion is actually phagocytosed by non‐tumorigenic cells, including hepatocytes and KCs. This not only potentially reduces the dose reaching LC cells but may also induce hepatotoxicity. The objective of active targeting is to improve the recognition capability of NMs specifically for LC cells, which may, in turn, reduce off‐target toxicity to normal liver tissue while maintaining therapeutic efficacy.^[^ ^266^, ^267^ ^]^
4. Addressing tumor heterogeneity and treatment resistance: The EPR effect is not uniformly significant across all regions of LC. Active targeting, particularly strategies directed against specific signaling pathways or cancer stem cell markers, can overcome intratumoral heterogeneity, enabling precise eradication of critical cell subpopulations responsible for therapeutic sensitivity or recurrence.^[^ ^268^, ^269^ ^]^

Overall, passive targeting establishes the fundamental basis for LC‐targeted therapy by enabling NMs accumulation in liver tumor regions, while active targeting provides a sophisticated approach to overcome the last‐mile delivery challenge. This strategic combination ensures more efficient and specific internalization of therapeutics by target LC cells, ultimately achieving enhanced therapeutic efficacy with minimized adverse effects.

### 4.3 Stimuli‐Responsive Targeting: Precision in Spatiotemporal Controlled Release

Stimuli‐responsive NMs can dynamically adjust their physical behavior or chemical structure in response to subtle changes in the external environment. This capability enables precise and controlled drug release at tumor sites, ensuring accurate drug delivery in terms of location, dosage, and timing. As a result, these NMs minimize potential damage to normal tissues, while maximizing therapeutic efficacy. This breakthrough has significantly advanced tumor research and diagnostics. Stimuli‐responsive NMs can achieve targeted therapy through various external stimuli, such as light, ultrasound, temperature, or magnetic fields, as well as internal stimuli, including pH levels, enzyme concentrations, redox gradients, and ATP.^[^ ^270^, ^271^ ^]^ These stimuli facilitate drug release from NMs, allowing for highly specific interactions with target cells and enhancing therapeutic effectiveness.

#### 4.3.1 NMs Respond to Internal Stimuli

Tumor tissues exhibit distinct cellular microenvironments owing to their unique physiological characteristics. Compared to normal tissues, the TME is significantly more acidic, making pH‐responsive NMs highly relevant in cancer immunotherapy.^[^ ^272^ ^]^ Although the pH of normal tissues is ≈7.4, tumor tissues display a pH range of 5.7–7.8. In addition, pH variations can be observed at the subcellular level, with late endosomes and lysosomes exhibiting notably lower pH values, typically ranging 4.5–5.5. Biopharmaceutical formulations incorporating pH‐sensitive carriers can respond to subtle pH fluctuations, facilitating endosomal escape and preventing the degradation of nucleic acids or proteins within lysosomes.^[^ ^273^ ^]^ Shi et al. developed a pH‐sensitive DOX@HmA nanoparticle system, which is self‐assembled from histidine‐zinc ion coordination and loaded with the chemotherapeutic drug DOX for TACE in LC treatment. This nanostructure remains stable owing to histidine‐zinc ion coordination and undergoes dissociation in the acidic TME, triggering the release of DOX. When emulsified with iodized oil, DOX@HmA NMs significantly enhance drug retention within tumor tissues and achieve sustained DOX release through a pH‐responsive mechanism.^[^ ^274^ ^]^ Zhang et al. designed a stepwise pH‐responsive NMs using a charge‐reversible branched starch‐based (CAPL) shell and a poly(β‐amino ester) (PBAE)/ PLGA core to encapsulate paclitaxel (PTX) and combretastatin A4 (CA4) for combined anti‐angiogenic and chemotherapeutic treatment of LC. The CAPL/PBAE/PLGA nanoparticles exhibit sequential responses to the mildly acidic TME (pH ≈ 6.5) and the endo/lysosomal compartments (pH ≈ 5.5) through CAPL β‐carboxylamide bond cleavage and the “proton sponge” effect of PBAE, respectively. This controlled release strategy ensures the efficient and orderly delivery of CA4 and PTX. In HepG2 tumor‐bearing mice, CAPL/PBAE/PLGA NMs exhibit excellent tumor‐targeting capabilities and significantly enhance the inhibitory effects of PTX and CA4 on tumor growth and angiogenesis.^[^ ^275^ ^]^

**Figure 5:**
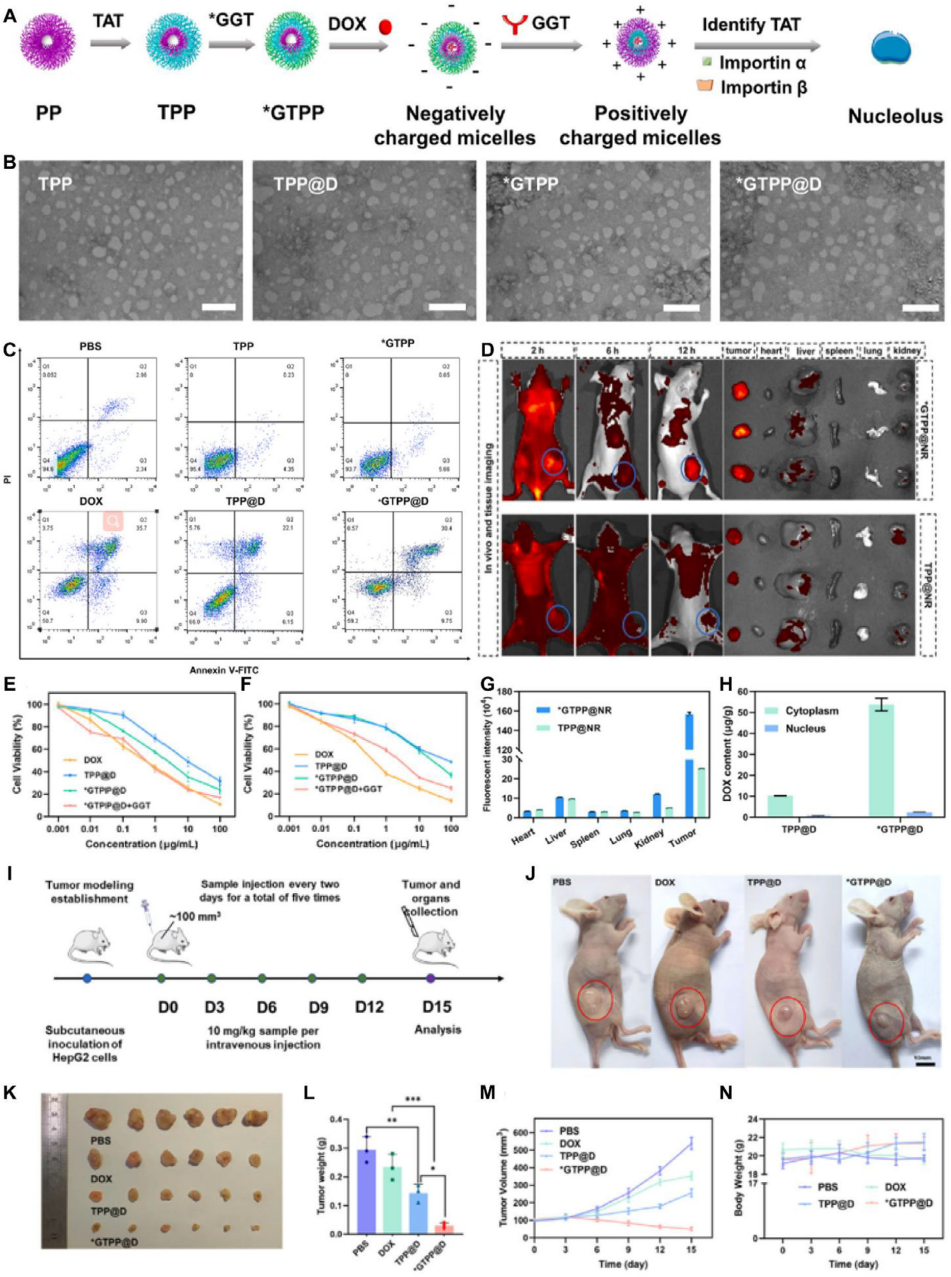
A) Schematic of the synthesis of *GTPP@D. B) TEM images of TPP, *GTPP, TPP@D, and *GTPP@D. Scale bar represents 100 nm. C) In vitro cytotoxicity, assessed by flow cytometry, showing apoptosis in HepG2 cells. D) Fluorescence imaging of tumor‐bearing mice and their tissues 12 hours after intravenous injection of two drug‐loaded micelles. E) Cytotoxicity of free DOX, TPP@D, *GTPP@D (with or without GGT pretreatment) in HepG2 cells. F) Cytotoxicity of free DOX, TPP@D, *GTPP@D, and *GTPP@D after GGT pretreatment in L‐929 cells. G) Nile Red content in tumor tissues and organs, detected by flow cytometry 12 h after intravenous administration. H) Quantification of DOX content in the nucleus and cytoplasm by high‐performance liquid chromatography. I) In vivo experimental protocol. J) Tumor morphology of labeled mice after treatment. K) Tumor dissection images at the end of the treatment experiment. L) Tumor weight after treatment in each group. M) Tumor volume. N) Changes in mouse body weight during the treatment period. Reproduced (Adapted) with permission.^[^ ^280^ ^]^ Copyright 2023, Elsevier.

Enzyme‐responsive NMs are promising candidates for designing intelligent drug delivery systems because of the overexpression of specific enzymes in certain tissues, particularly under pathological conditions. Upon detecting enzyme overexpression in patients with cancer, the use of enzymes as stimuli for site‐specific drug release have been proposed.^[^ ^276^ ^]^ In chemical action mechanisms, NMs can be designed to release drug payloads by degrading specific structural components upon exposure to enzymes. Based on this strategy, enzymatic transformation or degradation of NMs can also trigger therapeutic drug release, enabling the development of multimodal NMs with synergistic effects. In physical action mechanisms, enzyme‐responsive NMs can undergo macroscopic structural changes induced by enzymatic interactions, facilitating controlled drug release. In this approach, NMs can be surface‐modified with enzyme‐sensitive molecules to alter their physical properties. Enzyme activity dysregulation, commonly observed in various pathological conditions, provides novel opportunities for in vivo drug delivery.^[^ ^272^, ^277^, ^278^ ^]^ Kim et al. developed an enzyme‐triggered cascade‐responsive polymeric micelle system, consisting of a hydrophobic core of NQO1 enzyme‐responsive polycaprolactone (QPA‐PCL) and a hydrophilic PEG shell. This micelle system was designed as a DOX carrier for targeted treatment of LC overexpressing NAD(P)H:quinone oxidoreductase‐1 (NQO1). The QPA‐PCL/PEG (QPA‐P) micelles undergo an NQO1‐mediated quinone reduction, triggering a cascade cyclization reaction involving sequential intramolecular cyclization of amide and ester bonds. This process results in complete depolymerization of the hydrophobic core and micelle disassembly, enabling precise and controlled intracellular drug release.^[^ ^279^ ^]^ Yan et al. developed an enzyme‐triggered transcellular drug delivery system (GTPP@D), which consists of a γ‐glutamyl transpeptidase (GGT)‐responsive molecule (GGT), a nuclear‐targeting TAT peptide, and a Mal‐PEG‐PCL copolymer for efficient DOX delivery. This system specifically recognizes γ‐glutamyl substrates overexpressed in the TME, triggering a charge‐reversal mechanism, while leveraging TAT for active nuclear transport. It maintains a neutral charge in circulation to prolong its half‐life. However, upon reaching the tumor site, GGT hydrolyses the γ‐glutamyl group, inducing a positive charge that enhances cellular penetration and transcellular transport through electrostatic interactions. TAT further facilitates nuclear membrane penetration, ensuring DOX release within the nucleus to inhibit cancer cell division. Experimental results have shown that the enzyme‐triggered charge reversal and nuclear‐targeting synergy significantly enhance drug penetration depth (up to 95 µm) and nuclear drug concentration (0.68 µg/10⁷ cells within 4 h) in LC tissues. In a HepG2 xenograft tumor model, this system exhibited superior antitumor efficacy, achieving a 3.9‐fold increase in tumor inhibition rate compared to the control group. These findings provide a novel deep‐penetration drug delivery strategy for LC treatment (**Figure** **5**).^[^ ^280^ ^]^

GSH plays a critical role in microenvironmental regulation. Studies have shown that the concentration of GSH in tumor tissues is at least fourfold higher than that in normal tissues, with particularly elevated levels in some multidrug‐resistant tumors. This difference in redox conditions between tumor and healthy cells provides an opportunity for designing nanodrug carriers.^[^ ^281^, ^282^ ^]^ Compared to other stimuli, such as pH, redox‐responsive NMs have several advantages, including strong responsiveness to intracellular GSH, direct drug release into the nucleus and cell membrane, and stability in extracellular environments with low GSH levels.^[^ ^283^ ^]^ In tumor tissues, high concentrations of reduced GSH reduce disulfide bonds (─S─S─) to thiol groups (─SH). Consequently, most redox‐responsive NMs are designed using disulfide‐containing compounds. In normal tissues with lower reductive potential, disulfide bonds remain stable; however, in tumor environments, their reduction disrupts the drug delivery system, leading to a rapid drug release at the tumor site.^[^ ^284^, ^285^ ^]^ In one study, a disulfide‐bridged dihydroartemisinin (DHA) dimer nanoprodrug (DHA_2_‐SS) was developed. This system self‐assembles into NMs (SS NMs) through disulfide linkage, achieving an ultra‐high drug loading efficiency (>90%) and TME responsiveness. Under high intracellular GSH and ROS levels, the disulfide bond cleavage enables targeted DHA release. In vivo experiments using an H22 tumor‐bearing mouse model revealed that SS NMs exhibited superior tumor inhibition (64.3%) without systemic toxicity, validating their enhanced therapeutic efficacy through tumor accumulation and redox‐triggered drug release.^[^ ^286^ ^]^ Li et al. developed a novel redox‐responsive polyprodrug NM (polyprodrug NMs) for targeted siRNA delivery and synergistic cancer therapy. These NMs consist of a redox‐responsive 10‐hydroxycamptothecin (HCPT)‐based polyprodrug (polyHCPT) as the core, an amphiphilic lipid‐PEG shell, and a lactose acid (LA)‐decorated surface. This NM specifically accumulates in tumor tissues and targets LC cells through the recognition between LA and ASGPR. In the cytoplasm, the high GSH concentration breaks the disulfide bonds within polyHCPT, enabling the rapid release of intact HCPT molecules and siRNA, thereby achieving synergistic tumor growth inhibition.^[^ ^287^ ^]^

Owing to the unconventional concentration of ATP in the TME, ATP serves as a crucial stimulus for promoting the release of preloaded drugs from delivery carriers. Studies indicate that intracellular ATP (iATP) levels in cancer cells are significantly higher than those in non‐cancerous cells because of excessive glycolysis, rapid proliferation, and accelerated tumor growth. In the TME, ATP concentrations range 100–500 µm, markedly higher than the 10–100 nm observed in normal tissues. This significant difference facilitates the design of ATP‐mediated drug delivery systems.^[^ ^288^ ^]^ Ozalp et al. developed an ATP‐aptamer‐based molecular beacon hairpin platform, which integrates mesoporous silica nanoparticles (MSNs) covalently linked to ATP aptamers. In low‐ATP environments, the hairpin structure closes the nanopores, whereas in high‐ATP concentrations, ATP‐aptamer binding induces structural changes, enabling controlled release of fluorophores and drugs. Experimental results have shown a positive correlation between release efficiency and ATP concentration; however, interactions between silanol groups and red blood cell membrane (RBCm) present a risk of hemolysis.^[^ ^289^ ^]^ Huang and colleagues designed an ATP‐responsive manganese‐based bacterial material (*E. coli*@PDMC‐PEG), which comprises a PDA‐modified manganese‐porphyrin complex (PDMC) shell and a PEG protective layer. This biohybrid material is engineered to enhance tumor immunotherapy by synergistically activating the cGAS‐STING pathway. The PDMC‐PEG nanocoating responds to high ATP concentrations (0.1–0.4 mm) in the TME through a competitive ligand displacement mechanism, triggering the rapid release of Mn^2^⁺. Arterial interventional therapy delivering VNP20009@PDMC‐PEG effectively reduced the volume of in situ liver tumors in rabbits from 5.81 to 1.66 cm^3^ (*p* < 0.01) and decreased the number of lung metastases.^[^ ^290^ ^]^ Currently, studies on ATP‐responsive NMs drug release remain limited, and further investigation is required to validate their biosafety and clinical translation.

#### 4.3.2 NMs Respond to External Stimuli

Light is a non‐invasive modality that serves as an important trigger for the rapid and precise release of anticancer drugs. Since the 1980s, PDT has emerged as a promising treatment strategy that involves the combined use of photosensitizers and an appropriate light source.^[^ ^291^ ^]^ These photosensitizers exhibit low toxicity in the absence of light but selectively induce cancer cell death upon irradiation, thereby minimizing damage to normal tissues. In addition to the precise localization of tumors using well‐designed photosensitizers, spatial control of light exposure further reduces the risk of harming healthy tissues. This dual selectivity of phototherapy significantly mitigates the systemic toxicity associated with conventional chemotherapy and RT. Although only a limited number of approaches have been translated into clinical applications, numerous photosensitizers have been identified in research studies.^[^ ^292^ ^]^ Ongoing studies aim to optimize light‐responsive materials to achieve efficient and reproducible drug release profiles.

**Figure 6:**
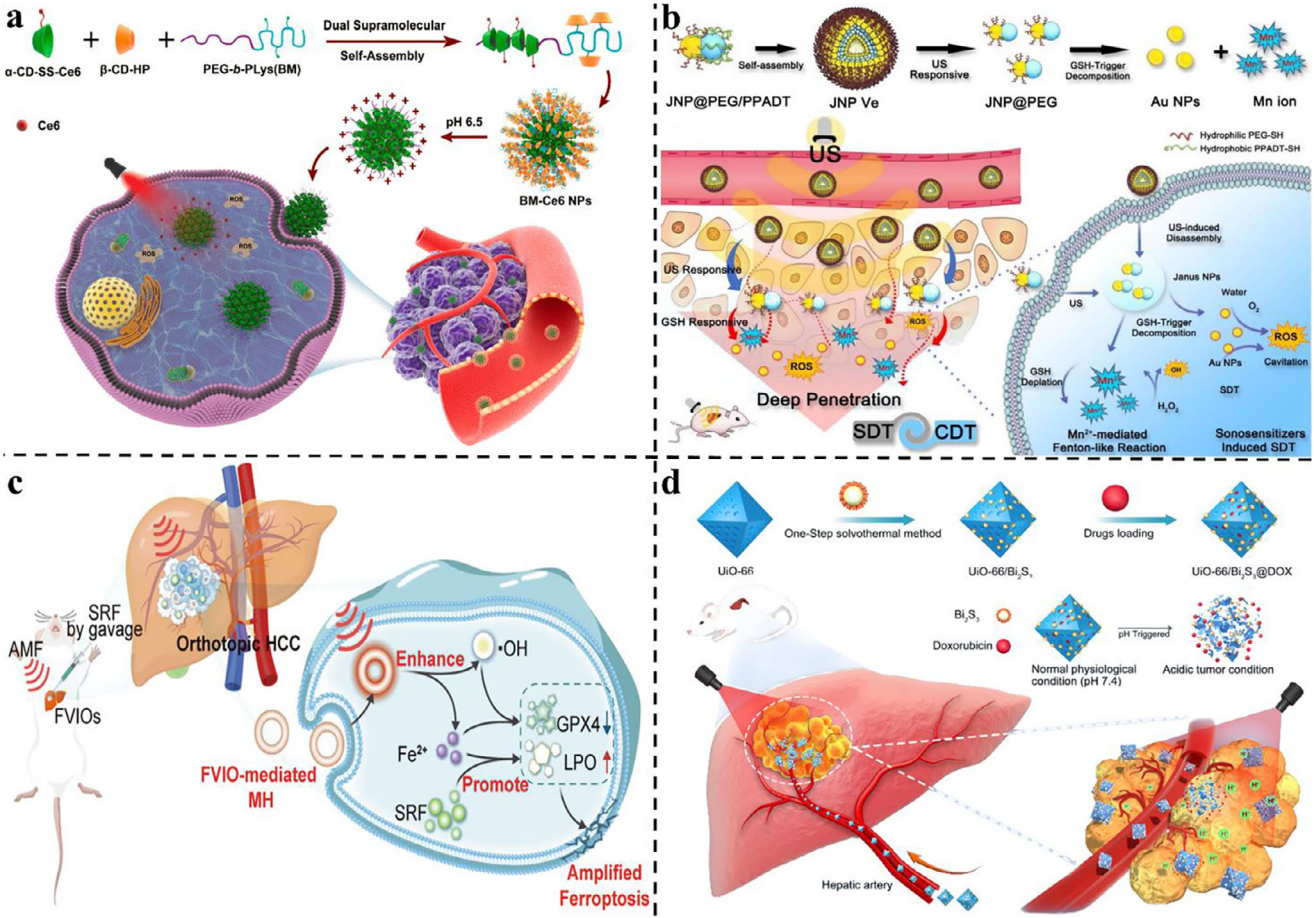
a) Schematic illustrating the preparation of BM‐Ce6 NMs through dual supramolecular assembly and their in vivo delivery for combined PDT. Reproduced (Adapted) with permission.^[^ ^295^ ^]^ Copyright 2022, Elsevier. b) Schematic of the self‐assembly of amphiphilic Janus Au‐MnO NMs into functional vesicles, which disassemble into smaller JNPs under ultrasound and GSH induction. These JNPs exhibit deep tumor penetration and are utilized for SDT and CDT. Reproduced (Adapted) with permission.^[^ ^303^ ^]^ Copyright 2019, John Wiley and Sons. c) Schematic illustrating the mechanism by which FVIO‐mediated MHT significantly enhances the sensitivity of LC cells to Sor‐induced ferroptosis. Reproduced (Adapted) with permission.^[^ ^312^ ^]^ Copyright 2024, American Chemical Society. d) Schematic of the preparation of UiO‐66/Bi_2_S_3_@DOX and its drug release under acidic TME conditions in combination with laser irradiation to generate effective PTT. Reproduced (Adapted) with permission.^[^ ^313^ ^]^ Copyright 2022, American Chemical Society.

Phototherapy primarily requires NIR light, which possesses deep tissue penetration capabilities, and can be categorized into three major types. The first is PTT, where light irradiation generates heat, directly inducing cancer cell death. The second is PDT, which activates photosensitizers to generate ROS responsible for killing cancer cells. Lastly, light‐induced heat can trigger the release of encapsulated drugs, thereby destroying cancer cells.^[^ ^293^ ^]^ These three approaches are often used in combination.^[^ ^294^ ^]^ Hu et al. developed NMs based on dual supramolecular self‐assembly. This system comprises a supramolecular host, PEG–polylysine block copolymer (PEG‐b‐Plys (BM)) modified with benzimidazole, along with functionalized guest molecules, α‐cyclodextrin–disulfide bond–chlorin e6 (α‐CD‐SS‐Ce6) and hydroxypropyl‐β‐cyclodextrin (β‐CD‐HP). The NMs load the photosensitizer Ce6 through the polyrotaxane interaction between α‐CD and PEG, whereas β‐CD‐HP encapsulates BM to regulate surface charge, aiming for TME‐activated PDT. In both subcutaneous and orthotopic LC models, the system showed excellent tumor targeting and PDT efficacy, achieving TME‐specific activation and precise intracellular drug release (**Figure** **6**a).^[^ ^295^ ^]^ Zhang et al. developed an aptamer‐conjugated nanosphere system composed of a GPC3‐targeting aptamer, PDA nanosphere core, platinum quantum dots (Pt QDs), and Ce6 photosensitizers. This multifunctional NM was designed as an integrated theranostic system for LC, enabling fluorescence imaging‐guided PTT and PDT combination therapy. The Apt‐Ce6‐PDA@Pt nanoformulation achieves active tumor targeting by specifically recognizing GPC3‐positive cancer cells through the aptamer, whereas PDA facilitates NIR absorption for efficient photothermal conversion. Moreover, Pt QDs catalyze hydrogen peroxide decomposition in the TME, generating oxygen to alleviate hypoxia and enhance Ce6‐mediated PDT efficiency. This system accurately delineates tumor boundaries through fluorescence imaging and enables the spatiotemporal coordination of PTT and PDT. Ultimately, the NMs showed significant tumor ablation and favorable biocompatibility in mouse models.^[^ ^296^ ^]^

Ultrasound is a periodic oscillating mechanical wave that induces mechanical or thermal stimulation, with a frequency exceeding the human auditory limit of 20 kHz. It plays a crucial role in modern medicine and is widely applied in both diagnostics and therapy. Low‐frequency ultrasound is primarily used for imaging, whereas high‐frequency ultrasound is used for tumor ablation and the removal of other abnormal masses. In medical applications, ultrasound with frequencies ranging 20–100 kHz is classified as low‐frequency ultrasound, which is commonly used to initiate ultrasound‐mediated tissue repair and induce the mechanical disruption of drug carriers. The design of nanoscale drug carriers responsive to ultrasound must meet three essential criteria. To begin with, these carriers should reliably and stably encapsulate therapeutic agents before ultrasound waves are applied to the tissue. In addition, they need to release the drug efficiently when exposed to specific ultrasound frequencies. Moreover, the carriers should enable real‐time monitoring of drug release, allowing for simultaneous imaging and therapy.^[^ ^297^, ^298^ ^]^ Ultrasound‐targeted microbubble destruction (UTMD) is an advanced technique that integrates ultrasound with functional carriers, providing significant advantages for chemotherapy drug delivery in solid tumors.^[^ ^299^ ^]^ Zhang and colleagues developed a nanobubble system consisting of CD133‐specific antibodies and miR‐107‐3p‐loaded nanobubbles. The assembly process involved biotin–avidin interactions and cationic lipid nanobubbles. Using a subcutaneous mouse tumor model, this system was combined with UTMD, and a marked accumulation of miR‐107‐3p/CD133 Ab‐NBs was observed at the tumor site. Their findings revealed that UTMD significantly enhances drug concentration in tumors, improving therapeutic efficacy.^[^ ^300^ ^]^

SDT is a non‐invasive treatment modality based on ultrasound stimulation, using sonosensitizers to generate ROS and induce acoustic cavitation effects for cancer cell destruction. This approach has several advantages, including deep tissue penetration, high therapeutic precision, strong controllability, and minimal side effects, making it particularly suitable for the treatment of deep‐seated tumors, while significantly improving patient compliance. Owing to the engineering capabilities of nanotechnology, NM‐based sonosensitizers can greatly enhance SDT efficacy by optimizing loading efficiency, improving targeting specificity, and strengthening binding affinity. For instance, modulating the surface properties of NMs can facilitate selective tumor accumulation, while simultaneously enhancing the conversion efficiency of ultrasound energy into ROS, providing new strategies for precise cancer treatment.^[^ ^301^, ^302^ ^]^ Yang et al. developed a novel sonosensitizer by grafting Janus Au‐MnO NMs with hydrophilic thiolated PEG and hydrophobic ROS‐sensitive polythioketal derivatives. These Janus Au‐MnO NMs self‐assembled into JNP vesicles (JNP Ve), which respond to both ultrasound and GSH stimuli. Upon ultrasound stimulation, the vesicles disassemble into smaller Janus Au‐MnO NMs with enhanced penetration capability. Subsequently, in the presence of high levels of GSH within tumors, these NMs further decompose into even smaller Au NPs and Mn^2^⁺ ions, generating large amounts of ROS for chemodynamic therapy (CDT). This intelligent NM simultaneously enables second NIR (NIR‐II) photoacoustic imaging and T1‐weighted magnetic resonance imaging, effectively inhibiting in situ liver tumor growth through synergistic SDT/CDT (Figure 6b).^[^ ^303^ ^]^

Temperature is an extensively studied stimulus and plays a crucial role in controlling the spatiotemporal drug release of stimulus‐responsive NMs. The elevated temperature characteristic of inflamed pathological sites and tumors serves as an internal trigger, whereas external temperature variations can activate thermoresponsive NMs. These properties make thermoresponsive NMs an attractive option, as they can rapidly respond to temperature changes. Another significant advantage of these NMs is their ability to be formulated as injectable solutions, allowing for implantation into diseased tissues without the need for surgical intervention. An ideal smart NM should retain its payload until the appropriate moment and release the encapsulated drug in a controlled manner within target tissues such as tumors.^[^ ^304^ ^]^ Shen et al. designed a thermoresponsive NM system (OXP@ZIF‐8/CTX@CPNPs) by modifying zeolitic imidazolate framework (ZIF‐8) with CS‐poly(N‐isopropylacrylamide) (C‐PNIPAM) to co‐deliver oxaliplatin (OXP) and cabazitaxel (CTX) in LC cells. These NMs exhibited thermoresponsive drug release, characterized by sustained and prolonged drug release profiles.^[^ ^305^ ^]^

In addition to external heat sources, intracellular mitochondria act as endogenous heat generators. Peng et al. synthesized a mitochondria‐targeting thermoresponsive NM composed of triphenylphosphine (TPP) for mitochondrial targeting, a HA shell, and a tetradecanol (TCA) core. This system encapsulates the uncoupler 2,4‐dinitrophenol (DNP) and the free radical initiator V057, enabling mitochondrial self‐heating to induce ICD for LC immunotherapy. The system achieves precise accumulation of NMs in tumor mitochondria through TPP‐mediated mitochondrial targeting and HA‐facilitated CD44 receptor‐mediated tumor targeting. Upon reaching the tumor site, the inherent mitochondrial temperature of 45 °C triggers the phase transition of TCA, leading to the release of DNP. The released DNP exacerbates mitochondrial heat production through the proton leakage mechanism, activating V057 decomposition and generating a radical storm, ultimately causing mitochondrial oxidative damage. When combined with a CTLA‐4 inhibitor, this strategy effectively suppresses deep‐seated in situ liver tumors and establishes long‐term immune memory.^[^ ^306^ ^]^

Magnetic force is considered an optimal external stimulus because of its minimal physical interaction with the human body compared to other stimuli such as light exposure, ultrasound, or temperature. When combined with magnetic NMs (MNMs), it serves as an ideal mechanism for selectively controlling drug accumulation and release in target tissues. Engineered MNMs can be functionalized, magnetically driven, and magnetically controlled, making them valuable carriers for biomedical targeting and drug delivery.^[^ ^307^, ^308^, ^309^ ^]^ These engineered MNMs can also be triggered by external stimuli, such as an alternating magnetic field (AMF), to achieve targeted and controlled drug release. The unique ability of MNMs to generate heat under high‐frequency AMF is known as the magnetothermal effect, also referred to as magnetic hyperthermia therapy (MHT). Superparamagnetic iron oxide nanoparticles (SPIONs) exhibit high magnetic stability and low toxicity, making them suitable for therapeutic applications when used in conjunction with a magnetic field.^[^ ^310^ ^]^ Jeon et al. delivered clinically approved MNMs conjugated with DOX to an LC animal model and exposed the animals to AMF for MHT, while monitoring tumor temperature variations. The results showed that MNMs combined with DOX increased the intratumoral temperature by 8.95 ± 1.31 °C, approximately sixfold that of the control group. In terms of tumor cell apoptosis, the apoptosis rate in the experimental group was approximately threefold higher than in the control group. These findings indicate that the intratumoral injection of DOX‐conjugated MNMs, in combination with AMF‐induced MHT, yields superior therapeutic effects compared to using either DOX or MNMs alone.^[^ ^311^ ^]^ Tang et al. investigated the potential of high‐performance ferromagnetic vortex‐domain iron oxide nanorings (FVIOs) to enhance MHT for overcoming Sor resistance in patients with LC. Their study confirmed that FVIO‐mediated MHT significantly increased the sensitivity of LC cells to Sor‐induced ferroptosis. This study provides a novel perspective on the combination of MHT and targeted LC therapy (Figure 6c).^[^ ^312^ ^]^

X‐rays and γ‐rays, owing to their superior tissue penetration and well‐established clinical applicability, have become ideal external stimuli for controlled drug release within deep‐seated tumors such as LC.^[^ ^314^ ^]^ The strategies for radiation‐induced drug release can be broadly classified into two categories. The first involves radiation‐activated prodrug strategies, in which prodrugs are designed to remain inert prior to irradiation and are activated by bioactive species generated post‐irradiation. For instance, ·OH produced by radiolysis of water can reduce diazonium compounds, while hydrated electrons (e^−^_a_q) efficiently activate N‐oxide prodrugs or cleave quaternary ammonium groups, enabling dose‐dependent precision drug control.^[^ ^315^ ^]^ In addition, radiation can induce apoptosis and upregulate Caspase‐3 activity, which specifically cleaves DEVD peptide linkers to release chemotherapeutic agents such as DOX.^[^ ^316^ ^]^ The second category involves radiation‐responsive NMs, where drugs are encapsulated within radiation‐sensitive nanomaterials that enable controlled release through material‐specific responses. For example, mesoporous organosilica NPs containing diselenide bonds undergo bond cleavage and degradation under the synergistic action of X‐rays and elevated ROS in the TME, releasing agents such as DOX or PD‐L1 inhibitors to achieve combined chemo‐immunotherapy.^[^ ^317^ ^]^ A more sophisticated approach employs inorganic scintillators that convert high‐energy photons (X‐rays or γ‐rays) into UV/visible light, which then activates surface‐loaded photosensitive molecules or photoresponsive polymers. This dual energy‐conversion process markedly enhances X‐ray utilization efficiency while reducing the required radiation dose.^[^ ^318^ ^]^ Despite these promising advances, several key challenges remain, including the development of organic scintillators with both high biocompatibility and energy‐conversion efficiency, elucidation of the precise molecular mechanisms underlying radiation–biomolecule interactions, and further reduction of clinically required radiation doses.^[^ ^319^ ^]^ Future research will focus on material innovation and multi‐mechanistic synergy, advancing radiation‐controlled drug release strategies toward higher precision, efficiency, and safety, ultimately offering transformative solutions for the treatment of LC.

#### 4.3.3 NMs Respond to Dual or Multiple Stimuli

The interior of a LC cell is a complex assembly of various components. Each cell is surrounded by specific receptors, enzymes, and other molecules.^[^ ^320^ ^]^ When designing NMs, external and internal stimuli are often combined to optimize drug release. These multifunctional NMs are more intelligent and provide higher loading efficiency and longer sustained release. Furthermore, multi‐responsive NMs can better sense minute changes in their environment.^[^ ^272^ ^]^ Liu and colleagues developed a nanocomposite material (UiO‐66/Bi_2_S_3_@DOX) based on a metal–organic framework (UiO‐66) and bismuth sulfide (Bi_2_S_3_), prepared using a one‐step solvothermal method and loaded with the chemotherapy drug DOX. This system was designed to enhance the synergistic therapeutic effect of TACE combined with PTT for LC. The UiO‐66/Bi_2_S_3_ uses the pH‐responsive properties of UiO‐66, which releases DOX in acidic TME, and the NIR photothermal conversion capability of Bi_2_S_3_, enabling targeted drug release and localized hyperthermia. Experimental results showed that this NM released 35.9% of DOX within 8 h under acidic conditions, and upon exposure to 808 nm laser irradiation, generated an efficient photothermal effect that increased the temperature to 77 °C within 4 min. This treatment significantly inhibited tumor growth in the N1S1 rat LC model, achieving a tumor volume inhibition rate of 65.2%. Furthermore, the system effectively downregulated the angiogenesis marker CD31 and promoted tumor cell apoptosis, confirming its synergistic therapeutic advantages (Figure 6d).^[^ ^313^ ^]^

Chen et al. developed a convenient integrated NM, FTY720@AM/T7‐TL, for the treatment of LC. This NM achieves charge reversal in an acidic environment and releases its drug payload upon NIR irradiation. Specifically, the platform consists of gold manganese dioxide (Au‐MnO_2_) NMs, which serve as a photosensitizer, photothermal agent, peroxidase catalyst, and T1 MRI agent, along with tetraphenylethene (TPE) for fluorescence imaging and FTY720 as the chemotherapy drug. These components are encapsulated in mixed liposomes. After entering the TME from the bloodstream, the acidic conditions cause the NMs to undergo a charge reversal, promoting their phagocytosis. Once inside the cells, the platform releases Au‐MnO_2_, TPE, and FTY720 under NIR stimulation. This dual external and internal stimulation effect has two key advantages. First, it ensures that the drug primarily interacts with cancer cells in the mildly acidic TME, and second, it minimizes damage to normal tissues by controlling the range of NIR irradiation.^[^ ^321^ ^]^

## 5 Immune Modulation: Reversing the Immunosuppressive Microenvironment of LC

**Figure 7:**
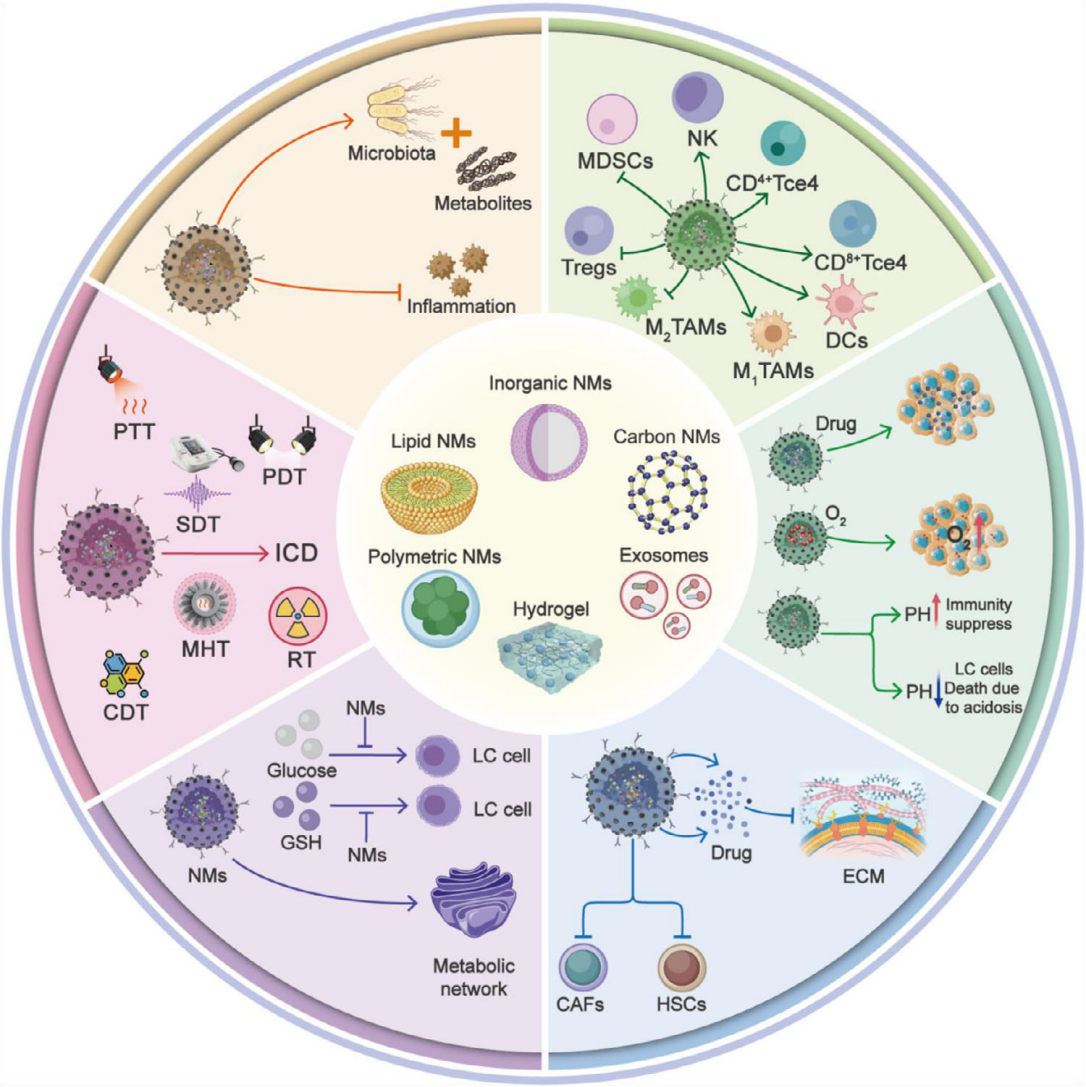
Various types of NMs reverse the immunosuppressive TME in LC through multiple mechanisms, including the modulation of immune‐related cells, remodeling of the abnormal physiological microenvironment, inhibition of ECM deposition, regulation of metabolic reprogramming, induction of ICD via multiple pathways, and modulation of the microbiota and its metabolites.

The immunosuppressive TME of LC is composed of multiple components. Using nanotechnology, this immunosuppressive TME can be counteracted through various strategies, including regulating immune‐related cells, remodeling the abnormal physiological microenvironment, inhibiting ECM deposition, and modulating metabolic reprogramming (inducing ICD and modulating microbiota and their metabolites). Furthermore, combining these approaches with ICB therapies can significantly enhance immune reversal. This section elaborates on these strategies in detail (**Figure** **7**).

### 5.1 Regulation of Immune‐Related Cells

NMs, with their advantages of precise targeted delivery, multimodal synergistic regulation, and biocompatibility, provide innovative strategies for reshaping the hepatic immune microenvironment at the cellular level. Through the design of functionalized NMs, the functional network of liver immune‐related cells can be regulated through three primary approaches: activating and enhancing the infiltration and function of NK and T cells, improving the antigen‐presenting capability and immune‐priming function of APCs, and inhibiting or reprogramming immunosuppressive cells to overcome local immune tolerance. These approaches have opened promising research directions for LC treatment.

#### 5.1.1 Activating and Enhancing the Infiltration and Function of NK and T cells

Studies have shown that the activation status of T lymphocytes and NK cells is regulated by interactions between membrane‐bound receptors and ligands at intercellular contact sites. This mechanism forms a fundamental molecular basis of immune regulation. Based on this biological principle, engineered NMs, functionalized with ICIs or other specific recognition elements, can mimic such biomolecular recognition interfaces.^[^ ^322^ ^]^ These systems enable targeted tumor therapy and significantly enhance immune function. This biomimetic strategy provides a novel approach for precise modulation of immune responses. In one study, iron oxide nanoparticles were conjugated with anti‐PD‐1 antibodies and systemically administered to mice bearing orthotopic LC tumors. Thirteen days after the final treatment, mice treated with the NM‐bound anti‐PD‐1 exhibited significant modulation of tumor‐infiltrating leukocytes, particularly through enhanced T cell activation and increased recruitment of M1 macrophages.^[^ ^323^ ^]^ In addition to serving as carriers for ICIs, NMs can also mimic ICI functions through alternative mechanisms. For example, Lee et al. used PD‐1 peptides as molecular templates to fabricate magnetic peptide‐imprinted poly(ethylene‐co‐vinyl alcohol) NMs (MPIP NMs) as artificial antibodies. Mechanistically, MPIP NMs bind to and block the inhibitory checkpoint PD‐1, thereby activating and stimulating NK cell‐mediated immune responses, which are essential for the efficacy of ICIs.^[^ ^324^ ^]^

**Figure 8:**
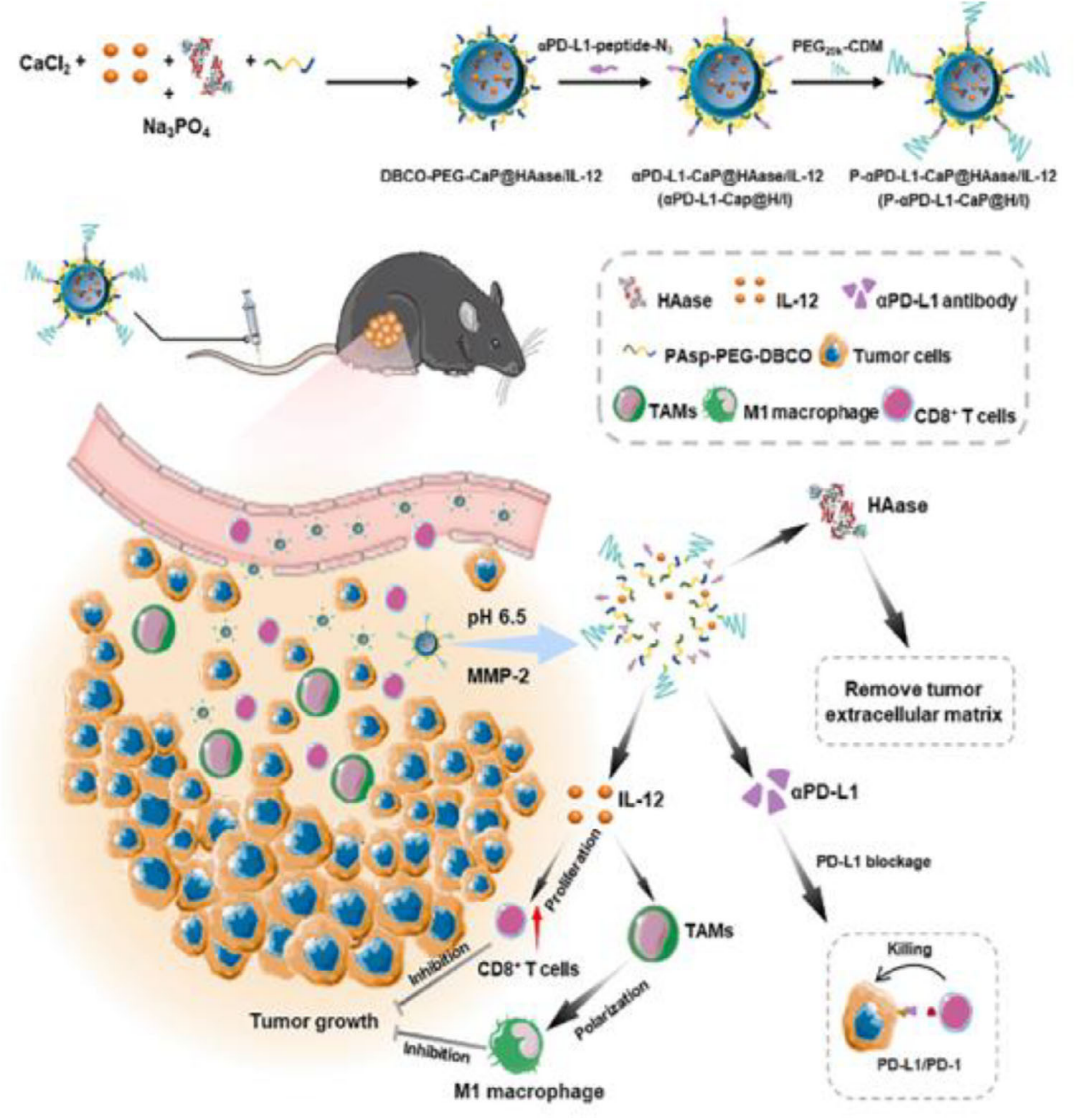
a) Schematic of the preparation of pH and MMP‐2 dual‐sensitive PEG shielded nanodrug P‐αPD‐L1‐CaP@HAase/IL‐12 for tumor immunotherapy, and its in vivo performance in promoting IL‐12 release and stimulating CTL proliferation. Reproduced (Adapted) with permission.^[^ ^58^ ^]^ Copyright 2023, Elsevier.

Molecular studies have revealed that cytokines such as IL‐12 and IL‐15 exhibit significant antitumor effects and have become a fundamental component of LC treatment frameworks.^[^ ^325^ ^]^ However, clinical translation is hindered by two major barriers, their intrinsic molecular characteristics result in rapid in vivo metabolism, and their narrow therapeutic window can easily trigger systemic toxicity.^[^ ^326^ ^]^ To overcome these pharmacological limitations, intelligent NMs have been engineered using liposomes or polymer‐based NMs to encapsulate cytokine proteins or their encoding mRNAs. This targeted delivery strategy enhances drug stability, improves hydrophilicity, and promotes tumor‐specific accumulation, thereby significantly reducing the off‐target toxicity associated with conventional cytokine therapies.^[^ ^327^ ^]^ Delivering IL‐12 through TME‐responsive NMs for efficient intratumoral release is an effective strategy to enhance its therapeutic efficacy. For instance, Xiao et al. developed a dual‐sensitive NM composed of pH and MMP‐2‐responsive polymers and CaP for co‐delivery of IL‐12 and αPD‐L1, with an outer detachable PEG coating. Acidic tumor conditions dissolve the CaP, promoting IL‐12 release and stimulating CTL proliferation (**Figure** **8**).^[^ ^58^ ^]^

In non‐viral RNA delivery systems, LNPs and cationic polymer‐based NMs are the two predominant platforms.^[^ ^328^, ^329^ ^]^ Despite the diverse mechanisms of RNA therapeutics, the delivery systems must fulfil two core requirements, targeted accumulation in specific organs, while evading immune clearance, and precise intracellular delivery through endocytosis.^[^ ^330^ ^]^ Cationic polymers, such as polyethyleneimine (PEI) and polylysine (PLL), form stable complexes with RNA through electrostatic interactions and exhibit unique advantages in cellular delivery. Lai et al. reported that IL‐12 mRNA‐loaded LNPs (IL‐12‐LNPs) could suppress MYC‐driven LC progression. Treatment with IL‐12‐LNPs induced significant infiltration of activated CD44^+^ CD3^+^ CD4^+^ helper T cells into tumors and increased IFN‐γ production.^[^ ^331^ ^]^ To deliver IL‐15 mRNA, Liu et al. developed a biodegradable galactose‐PEG‐conjugated copolymer (LA‐PegPI). This gene delivery platform enabled targeted transfection of hepatoma cells through ASGPR‐mediated uptake and PEG‐induced steric stabilization. PEGylation improved the physiological stability of the NMs and reduced clearance by the reticuloendothelial system, whereas the galactose ligand enhanced hepatocyte uptake through receptor‐mediated endocytosis. Results showed that intraperitoneal injection of these NMs led to efficient IL‐15 expression in hepatic parenchymal cells of tumor‐bearing mice, significantly promoting CD8^+^ T cell and NK cell proliferation, upregulating antitumor cytokines such as IFN‐γ and TNF, prolonging survival, and reducing tumor weight by 60%.^[^ ^332^ ^]^ This study presents a novel delivery platform for immune gene therapy in LC.

In addition to conventional NM‐based drug delivery systems for immunomodulatory agents, pure drug nanoassemblies (PDNAs), which are formed by the self‐ or co‐assembly of drug molecules without carriers, have recently garnered significant attention. Their simple and reproducible preparation methods help address key challenges in NMs, including large‐scale production, quality control, and clinical translation. Acting as both carriers and cargos, carrier‐free PDNAs have exceptionally high drug‐loading capacities, even up to 100%. Moreover, PDNA‐based combination therapies hold promise in tackling some of the most formidable challenges in cancer treatment, such as tumor metastasis and drug resistance. Le et al. designed a controllable co‐assembled dual‐drug NM for targeted and synergistic treatment of LC. Through a one‐pot assembly approach, they coated a concentrated NM core composed of SOR, ursolic acid (UA), and indocyanine green (ICG) with a cell‐penetrating peptide and an aptamer, successfully constructing a dual‐functionalized NM (USILA NMs). These USILA NMs exhibited enhanced cellular uptake and cytotoxicity in HepG2 and H22 cells, both of which highly express epithelial cell adhesion molecule (EpCAM). In addition, their efficacy was significantly improved by co‐administering iRGD peptide or a PD‐L1 antibody to reverse the immunosuppressive TME. Compared to the control group, tumor inhibition rates for USILA NMs combined with iRGD peptide or PD‐L1 antibody were 72.38% and 67.91%, respectively, showing good therapeutic safety.

#### 5.1.2 Activating and Enhancing the Function of APCs

Activation of APCs is essential in LC immunotherapy, as enhancing their antigen presentation capabilities can effectively augment specific T cell responses. Nanovaccines have emerged as a promising strategy for safe and targeted drug delivery. Notably, the co‐delivery of tumor antigens and immunoadjuvants through NMs has become a focal point in LC immunotherapy.^[^ ^333^ ^]^ TLR agonists, such as CpG oligodeoxynucleotides (CpG ODNs), are commonly used as vaccine adjuvants to promote DC activation and T cell priming.^[^ ^334^ ^]^ Meng et al. developed lipid‐coated iron oxide NMs (IONP‐C/O@LP) as nanovaccines capable of co‐delivering peptide antigens and CpG DNA adjuvants to DCs through membrane fusion and endocytosis. This dual uptake mechanism synergistically activates immature DCs. In addition, the iron oxide core induces intracellular ROS, further promoting DC maturation. Accumulation of IONP‐C/O@LP in draining lymph node DCs effectively increases antigen‐specific T cells in tumors and spleens, inhibits tumor growth, and improves survival rates.^[^ ^335^ ^]^ In addition to co‐delivery strategies, RNA‐based vaccines have shown significant therapeutic potential. They can express multiple antigens directly in the cytoplasm following a single immunization, eliciting both humoral and cellular immune responses to counteract immunosuppressive TMEs. Although RNA vaccines possess strong immunogenicity without adjuvants, protecting RNA from enzymatic degradation before reaching tumors remains challenging. A prior study developed a targeted liposome–polycation–DNA (LPD) NM complex that effectively safeguards RNA, exhibiting excellent serum stability.^[^ ^336^ ^]^ Studies have shown that the combination of activated DCs and ICIs produces a strong synergistic antitumor effect in the treatment of various malignancies, including LC. Researchers have reported a dual‐catalytic oxide nanosponges (DON) system that serves as both a remote catalytic enhancer and a DCs programming inducer for programmable immunotherapy. Experimental results demonstrated that the catalytic DON combined with ICIs effectively inhibited tumor growth and significantly improved the 40‐day survival rate in metastatic tumor models.^[^ ^337^ ^]^

**Figure 9:**
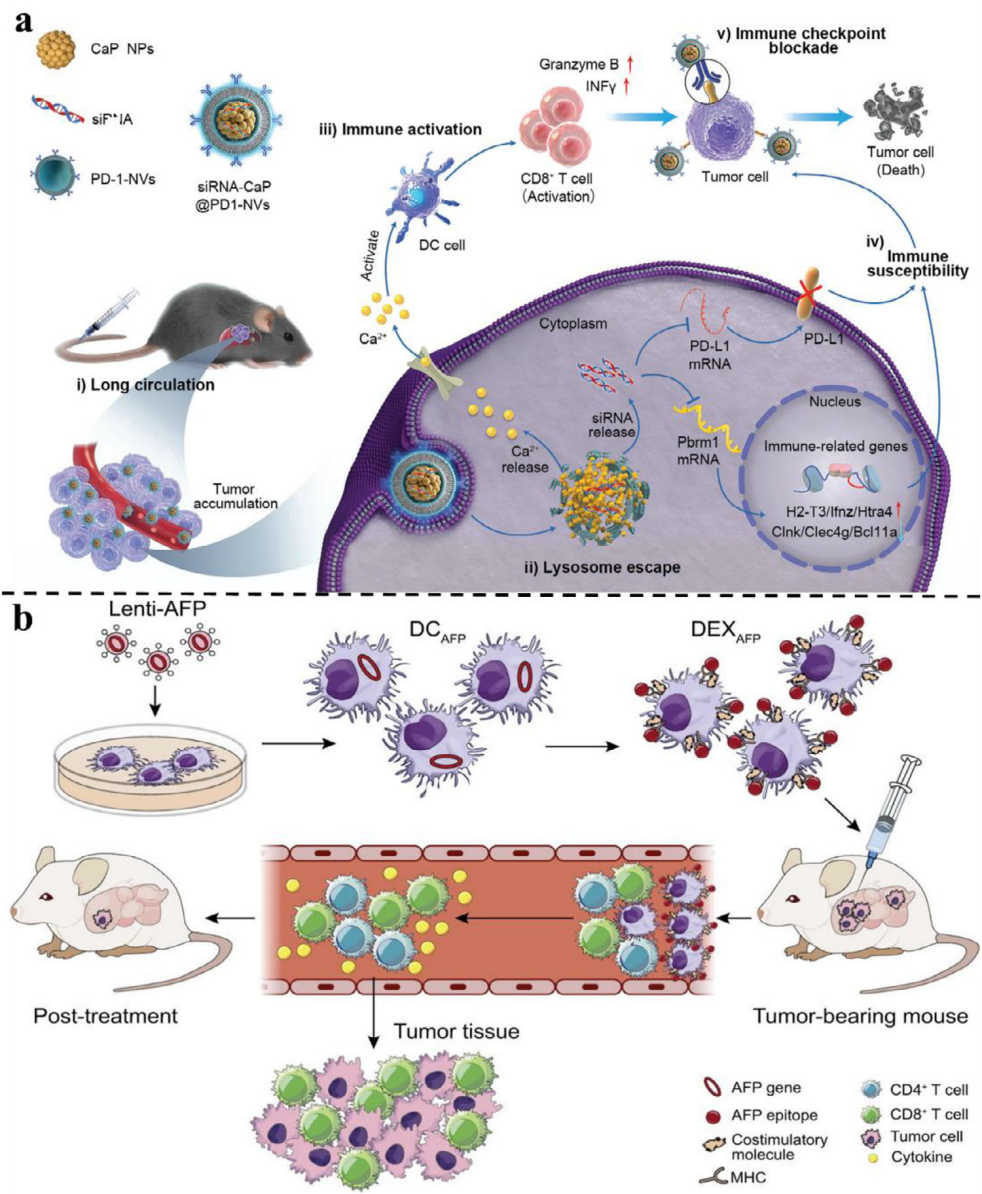
a) Schematic of siRNA‐CaP@PD‐1‐NVs for dual‐targeted immunogene therapy. Calcium ion adjuvant enhances DC maturation, increases CD8^+^ T cell infiltration and activation, and promotes the formation of long‐term immune memory, ultimately improving immune checkpoint blockade therapy. Reproduced (Adapted) with permission.^[^ ^341^ ^]^ Copyright 2024, John Wiley and Sons. b) Schematic of the preparation of DEXAFP and its in vivo mechanism of action. Reproduced (Adapted) with permission.^[^ ^343^ ^]^ Copyright 2017, Elsevier.

The advent of biomimetic NMs provides innovative avenues for immunotherapeutic drug delivery. In cancer treatment, cell membrane‐derived nanovesicles and extracellular vesicles are frequently used as biomimetic delivery systems. Integrating NMs with cell membranes endows them with the inherent biological properties of source cells. These platforms are advantageous because of their ease of genetic modification, intrinsic targeting capabilities, and immunogenicity enhancement.^[^ ^338^, ^339^, ^340^ ^]^ Sun et al. engineered HEK 293FT cells to overexpress PD‐1‐mCherry fusion proteins on their surfaces. After membrane isolation and complexation with CaP cores, they constructed virus‐like biomimetic nanovesicles (siRNA‐CaP@PD‐1‐NVs). Designed for co‐delivery of siRNA targeting PD‐L1/Pbrm1 genes and ICIs, these carriers facilitate dual‐targeted immunogene therapy for LC. The pH‐responsive dissolution of CaP cores enables lysosomal escape and siRNA release, whereas PD‐1‐NVs specifically bind to tumor cell PD‐L1 for targeted delivery. Experiments revealed enhanced DC maturation, increased CD8^+^ T cell infiltration and activity, and long‐term immune memory formation through calcium ion adjuvant effects, culminating in complete tumor eradication and prevention of recurrence and metastasis (**Figure** **9**a).^[^ ^341^ ^]^ Apart from cell membrane‐based biomimetic NMs, extracellular vesicles secreted by cells, owing to their role in intercellular communication and biocompatibility, have become primary sources for biomimetic NMs.^[^ ^342^ ^]^ A previous study tested exosomes derived from AFP‐expressing DCs (DEX_AFP_) in three different LC mouse models, monitoring tumor growth and microenvironment. The results confirmed their feasibility and efficacy in monotherapy for LC, highlighting the potential of DEX_AFP_ as a delivery vector (Figure 9b).​^[^ ^343^ ^]^

The innate cGAS‐STING immune signaling pathway plays a pivotal role in DC activation, antigen presentation, and T cell‐mediated immune responses. The second messenger 2’,3’‐cyclic GMP‐AMP (cGAMP) activates STING, inducing type I interferon production, promoting DC maturation, and mediating T cell cross‐priming for robust antitumor immunity.^[^ ^344^ ^]^ Various STING agonists, including ions, ionizable lipids, small molecules, cell membrane dsDNA, and polymers, have been developed.^[^ ^345^ ^]^ However, the clinical efficacy of cGAMP is hindered by instability, poor cellular permeability, and rapid clearance. Moreover, intracellular cGAMP‐bound STING translocate from the endoplasmic reticulum to the Golgi and then to lysosomes for degradation, attenuating STING pathway activation. Therefore, dual‐function NM delivery strategies that simultaneously promote STING activation and inhibit its degradation holds significant potential for enhancing antitumor immune responses.^[^ ^346^ ^]^ To synergistically enhance STING pathway activation and prevent STING degradation, Wang et al. proposed “three‐in‐one” NMs (IAHA‐LaP/siPTPN6 NMs) comprising cGAMP, lanthanum ions (La^3^⁺), and PTPN6 siRNA. In vitro results indicated that La^3^⁺ significantly promotes cGAMP‐mediated STING pathway activation by enhancing phosphorylation of STING, TBK1, IRF3, and NF‐κB p65. Compared to mixtures of La^3^⁺ and cGAMP, these NMs increased secretion of IFN‐β (2.4‐fold), IL‐6 (1.5‐fold), and TNF‐α (1.4‐fold), thereby promoting DC maturation. In vivo, IAHA‐LaP/siPTPN6 NMs elevated the percentage of mature DCs in tumor‐draining lymph nodes, facilitated CD8^+^ T cell infiltration into tumors, and significantly inhibited primary tumor growth.^[^ ^347^ ^]^ This NM design provides a novel strategy for enhancing STING pathway activation.

#### 5.1.3 Regulating Cells Associated with Immunosuppression

In addition to modulating NK cells, T cells, and APCs, NMs can reverse the immunosuppressive TME of LC by targeting immunosuppressive cells such as MDSCs, Tregs, and M2 phenotype TAMs. These cells contribute to tumor progression by secreting immunosuppressive factors, inhibiting effector T cell functions, and promoting angiogenesis. MDSCs exacerbate immune evasion in LC by suppressing CD8⁺ T cell activity and promoting Treg differentiation.^[^ ^348^ ^]^ Studies have shown that NMs can regulate MDSC function by targeting exosome secretion. For instance, Zhu et al. developed a custom‐designed NM (AM/GW@PDA) incorporating GW4869 (a neutral sphingomyelinase inhibitor) and amlodipine, leveraging the multifunctional properties of PDA. This formulation enabled precise and tumor‐specific drug release, synergistically inhibiting both exosome biogenesis and PD‐L1 expression, reducing MDSC infiltration, and restoring CTL function.^[^ ^349^ ^]^ Tregs suppress antitumor immune responses through molecules such as CTLA‐4 and IL‐10. Surface‐modified NMs can specifically target Treg‐related signaling pathways. For example, liposomal NMs loaded with rapamycin block Treg differentiation by inhibiting the mTOR pathway, while promoting effector T cell proliferation.^[^ ^350^ ^]^ Another strategy involves delivering TLR agonists to activate DC antigen presentation, thereby disrupting Treg‐mediated immune tolerance.^[^ ^351^ ^]^

Current TAM‐targeted therapies primarily follow two strategies, depletion or functional reprogramming.^[^ ^352^ ^]^ Although macrophage depletion may yield short‐term benefits in early‐stage tumors, it presents limitations by eliminating antitumor M1 phenotype macrophages and posing risks such as increased infections and homeostatic imbalance in normal tissues. Therefore, attention has shifted to reprogramming tumor‐promoting M2 phenotype TAMs into tumoricidal M1 phenotype, a promising translational approach.^[^ ^353^ ^]^ Several studies have used NMs to target TAM‐associated signaling pathways, such as STAT, NF‐κB, PI3Kγ/AKT, and ROS, to reprogram TAMs and reverse the immunosuppressive TME. The JAK‐STAT pathway plays a key role in TAM polarization, with STAT1 activation correlating with M1 markers and STAT3 activation correlating with M2 markers. IFN‐γ promotes M1 polarization through JAK1/JAK2‐mediated phosphorylation of STAT1 and induction of IRF5 transcription.^[^ ^354^, ^355^ ^]^ Zheng et al. developed a genetically engineered bacterial biomimetic vesicle (IFN‐γ BBV), in which IFN‐γ molecules were anchored to the vesicle surface through the ClyA protein, forming a nano‐assembled membrane pore structure. This engineered vesicle system was designed to act as a delivery platform for both pathogen‐associated molecular patterns (PAMPs) and the immunoregulatory cytokine IFN‐γ, with the goal of reprogramming TAMs and remodeling the immunosuppressive TME. IFN‐γ BBVs activate the TLR/NF‐κB pathway through surface‐presented PAMPs, and synergistically engage the JAK‐STAT signaling pathway mediated by IFN‐γ. This dual‐pathway activation efficiently converts M2 phenotype TAMs into the tumoricidal M1 phenotype, thereby reprogramming TAMs and transforming the “cold” TME into a “hot” one, providing a novel multifunctional platform for cancer immunotherapy.^[^ ^356^ ^]^

Moreover, the ROS signaling pathway has been shown to mediate MDSC‐induced immunosuppression as well as trigger the differentiation and polarization of M2 phenotype TAMs.^[^ ^357^ ^]^ During the process of incomplete radiofrequency ablation (iRFA)‐induced metastasis of residual LC, iRFA leads to abnormally elevated ROS levels in remaining LC cells, which enhances tumor cell invasiveness, promotes M2 phenotype macrophage polarization, and ultimately accelerates LC metastasis. This highlights the need for an effective ROS scavenger. Polyoxometalates (POMs), a type of molybdenum‐based nanocluster, are potent ROS scavengers; however, their small size limits renal clearance, reducing their efficiency in eliminating iRFA‐induced ROS. To address this limitation, Yang et al. developed an injectable coacervate‐based delivery system loaded with POM (POM@Coa), which enables sustained ROS elimination through controlled release. POM@Coa significantly reduces LC invasiveness, reverses macrophage polarization from M2 to M1 phenotype, enhances CD8⁺ T cell infiltration and activation, and ultimately inhibits LC metastasis.^[^ ^358^ ^]^ Another study reported that exosomes derived from H22 cells were used to encapsulate polymeric iron oxide NPs loaded with chlorin e6 (PIONs@E6). These engineered exosomes enhanced antitumor immunity by modulating the ROS signaling pathway and reprogramming TAMs toward the M1 phenotype.^[^ ^359^ ^]^

### 5.2 Remodeling the Abnormal Physiological Microenvironment

#### 5.2.1 Alleviating Hypoxia

**Figure 10:**
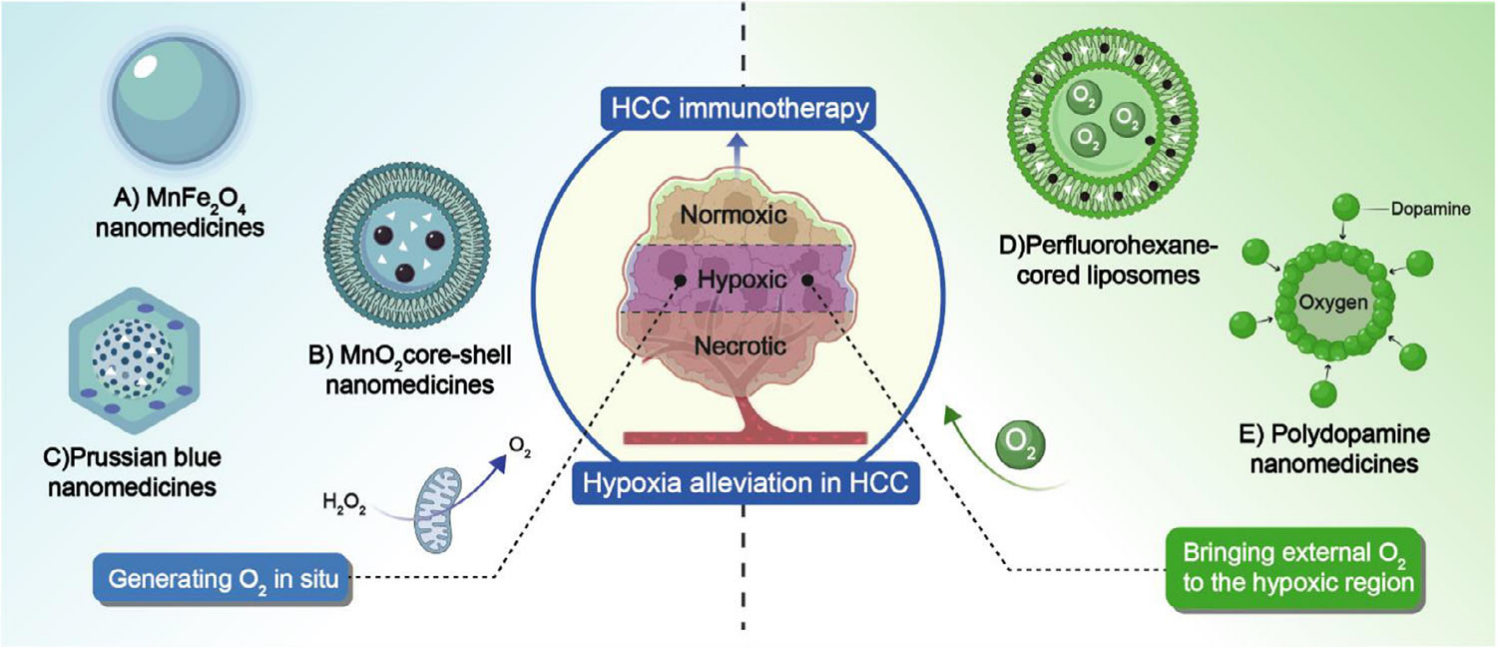
Nanomaterials alleviate hypoxia in LC treatment. Metal oxide NMs, such as MnO_2_ and MnFe_2_O_4_, as well as Prussian blue, can catalyze the decomposition of H_2_O_2_ to generate O2. Additionally, NMs have been developed as oxygen carriers to deliver external oxygen to hypoxic regions of LC.

Previous studies have shown that hypoxia‐driven TMEs directly suppress the cytotoxic activity of CD8⁺ T cells as well as promote the infiltration of immunosuppressive cells. Consequently, targeting hypoxia has become a promising strategy for NM based immunotherapy of LC. Currently, two primary approaches are used to alleviate hypoxia, generating oxygen in situ at the tumor site using NMs, and delivering exogenous oxygen to hypoxic regions through NMs (**Figure** **10**).^[^ ^360^ ^]^

In situ oxygen generation is primarily achieved using inorganic biomaterials (such as metal oxides and metals) or biomaterials with catalase‐like activity to decompose abundant H_2_O_2_ present in tumors into molecular oxygen.^[^ ^361^ ^]^ Among these, metal oxides are the most widely used oxygen‐generating agents, with manganese‐based oxides being particularly common in NMs development. Drug resistance, driven by mechanisms including the presence of CSCs, remains a major obstacle to improving the therapeutic efficacy of Lenvatinib (Len) in LC.^[^ ^362^ ^]^ To overcome this challenge, Yang et al. developed a NM (siCD24‐Len‐MnO@PLAP) by co‐loading manganese oxide (MnO), Len, and anti‐CD24 siRNA (siCD24) into micelles composed of a triblock copolymer consisting of mPEG, poly(L‐lysine) (PLLys), and poly(aspartic acid)‐N‐(2‐aminoethyl)piperidine (PAsp(PIP)). This NM is capable of releasing Len and generating Mn^2^⁺ and O_2_ in response to the TME. In a mouse model of LC, it exhibited significant anticancer effects by enhancing Len efficacy through the downregulation of CD24 and HIF‐1α expression.^[^ ^363^ ^]^

Catalase or catalase‐mimicking biomaterials are frequently used to decompose H_2_O_2_ in the TME, thereby generating oxygen. FDA‐approved Prussian blue (PB) NMs possess excellent photothermal conversion efficiency for PTT as well as exhibit catalytic activity, effectively decomposing excess H_2_O_2_ to release oxygen.^[^ ^364^ ^]^ Wang et al. developed a novel composite nanozyme system comprising mesoporous silica and cerium oxide NMs (MSN‐Ce@SP/PEG), which exhibits potent catalase activity to convert intracellular H_2_O_2_ into oxygen. This activity is significantly enhanced under photothermal conditions, effectively suppressing the recurrence and metastasis potential of drug‐resistant LC cells.^[^ ^365^ ^]^

NMs generate oxygen in situ within tumors as well as serve as oxygen carriers to deliver exogenous O_2_ to hypoxic regions. Common nanoscale oxygen carriers include perfluorohexane (PFH) and oxygen microcapsules constructed using PDA.^[^ ^366^, ^367^ ^]^ Hypoxia is a major barrier to the efficacy of RT in LC. Dai et al. designed a PDA NM‐stabilized oxygen microcapsule through interfacial polymerization, encapsulating oxygen within the NM shell. These capsules exhibit excellent aqueous dispersibility and biocompatibility, rapidly increasing and sustaining oxygen concentration in hypoxic environments, making them an efficient and stable oxygen delivery system. In LC mouse models, local injection of oxygen microcapsules significantly enhanced RT efficacy, reduced TAM numbers, and reprogrammed pro‐tumor M2 phenotype TAMs into anti‐tumor M1 phenotype TAMs.^[^ ^366^ ^]^ Clinically, Sor treatment activates the CXCR4/SDF‐1α axis, exacerbating hypoxia in LC and contributing to tumor progression, metastasis, immunosuppression, and ultimately, Sor resistance. Notably, Yu et al. developed a multifunctional oxygen delivery NM based on PFH‐core liposomes (PFH@LSLP), modified with CXCR4 antagonist peptide LFC131 and co‐loaded with Sor and CSF1/CSF1R inhibitor PLX3397. In both H22 tumor‐bearing mice and patient‐derived xenograft (PDX) models, PFH@LSLP effectively overcame Sor resistance by alleviating hypoxia, regulating drug resistance‐related genes, and remodeling the immune microenvironment.^[^ ^367^ ^]^

#### 5.2.2 Regulating the Acidity of TME

Numerous studies have shown that LC cells undergo significant metabolic alterations and gene reprogramming within the acidic TME.^[^ ^368^ ^]^ In addition to cancer cell polarization changes, elevated proteolytic activity of TAMs and fibroblasts, and suppression of immune surveillance, LC cells in an acidic TME acquire enhanced motility, which in turn promotes local invasion and distant metastasis. Moreover, the acidic TME impairs the functions of CD8⁺ T cells, NK cells, and DCs; facilitates the recruitment of TAMs and MDSCs; and upregulates immune checkpoint molecules such as PD‐L1 and CTLA‐4.^[^ ^369^, ^370^, ^371^ ^]^ Thus, in addition to serving as an endogenous trigger for pH‐responsive NM drug delivery systems, the acidic TME is considered a valuable therapeutic target. To enhance therapeutic efficacy against LC, strategies that focus on modulating the acidity of the TME have been developed. One widely explored approach involves the use of NMs to neutralize the acidic environment and elevate the pH, thereby mitigating immunosuppression and improving treatment response. Alternatively, another approach aims to exacerbate the already acidic TME by inducing intracellular acidosis in LC cells, thereby directly promoting cancer cell death through metabolic disruption.

Calcium carbonate (CaCO_3_), an insoluble calcium‐based biomineral, has attracted significant attention for its biocompatibility and pH‐dependent dissolution, making it an excellent material for constructing pH‐responsive drug delivery systems.^[^ ^372^ ^]^ Chen et al. showed that locally administered CaCO_3_ NMs loaded with anti‐CD47 antibodies synergistically inhibited both postoperative tumor recurrence and distant metastasis by sequentially scavenging protons and activating innate and adaptive antitumor immunity.^[^ ^373^ ^]^ Li et al. developed lipid‐coated CaCO_3_@CuO_2_ NMs (CaCO_3_@CuO_2_@L), which release CuO_2_ under acidic conditions, while simultaneously increasing pH, depleting GSH, and boosting oxygen production. In LC cells, these NMs modulated acidity, GSH levels, and hypoxia through a “triple mechanism,” providing valuable insights into comprehensive therapeutic strategies.^[^ ^374^ ^]^ Sodium bicarbonate (NaHCO_3_) NMs also possess the ability to regulate tumor acidity. Ding et al. fabricated simple, drug‐free NaHCO_3_ NMs using a rapid microemulsion method for application in cancer immunotherapy. The mildly alkaline NaHCO_3_ neutralized excess lactic acid and adjusted lactate metabolism, thereby reversing the immunosuppressive effects of the acidic TME. This approach significantly inhibited tumor growth and metastasis and enhanced antitumor immunity.^[^ ^375^ ^]^ Other inorganic NMs, such as calcium phosphate‐based NMs, have also been shown to be effective in treating various solid tumors.^[^ ^376^ ^]^

In addition to neutralizing extracellular acidity, other innovative strategies have aimed to induce intracellular acidosis, leveraging the fact that a slightly alkaline intracellular pH is essential for both normal and malignant cell survival. Studies have shown that inhibiting monocarboxylate transporters (MCTs) using small interfering RNAs or specific inhibitors leads to the accumulation of H⁺ ions and/or lactate within LC cells, thereby inducing intracellular acidosis.^[^ ^377^ ^]^ Wang et al. proposed a precise therapeutic strategy that disrupts tumor metabolic symbiosis by enhancing pyruvate levels and promoting acidification within LC cells. For this method, PEGylated gold and lactate oxidase‐modified aminated dendritic mesoporous silica loaded with lonidamine and ferrous sulfide (PEG‐Au@DMSNs/FeS/LND@LOX) was used. Under acidic conditions, the NMs released hydrogen sulfide gas, which, in combination with LND (an MCT inhibitor), resulted in lactate accumulation and excessive proton buildup, triggering intracellular acidosis. This disruption of the lactate–pyruvate axis led to metabolic collapse as well as enhanced the efficacy of CDT by promoting the Fenton reaction.^[^ ^378^ ^]^ In summary, these studies highlight that the acidity of the TME serves as an effective therapeutic target for the development of innovative cancer treatments, either by neutralizing extracellular acidity or inducing intracellular acidosis in LC cells.

### 5.3 Inhibiting ECM Deposition

The development and progression of LC, along with the surrounding TME, are significantly influenced by the disorganized ECM, which renders cancer cells sensitive to mechanical stress, signaling, and structural alterations.^[^ ^379^ ^]^ Efforts to design intelligent NMs to reduce ECM deposition have primarily focused on regulating HSCs and CAFs, either directly or indirectly. Activated HSCs overproduce ECM components. To address this, Luo et al. developed an NM targeting the Golgi apparatus of activated HSCs. Using a thin‐film hydration–high‐pressure homogenization technique, they fabricated chondroitin sulfate‐modified lipid nanoparticles (CSNs) loaded with DOX and retinoic acid (RA). The resulting DOX+RA‐CSNs were effectively internalized by both cultured SMMC‐7721 LC cells and HSCs, where they accumulated in the Golgi apparatus, disrupted its function, and suppressed ECM production. Immunofluorescence (IF) and IHC analyses of liver tissue revealed a significant reduction in ECM component expression.^[^ ^380^ ^]^ CAFs also play a critical role in supporting cancer growth, metastasis, and resistance to chemotherapy or ICIs through mechanisms such as angiogenesis, ECM remodeling, and the secretion of tumor‐promoting and immunosuppressive cytokines, chemokines, and growth factors.^[^ ^381^ ^]^ Therefore, targeting CAFs represents another strategy to suppress ECM accumulation. One study reported a liposomal oxymatrine system modified with a CFH peptide (CFHKHKSPALSPVGGG) with high affinity for Tenascin‐C, designed to inactivate CAFs by reversing EMT. When combined with a berberine‐loaded liposomal complex, this system significantly enhanced antitumor efficacy in a xenograft nude mouse model, reversed EMT in vivo, and markedly reduced collagen levels, facilitating deep tumor penetration of NMs. Interestingly, this system also reprogrammed TAMs toward the M1 phenotype and activated NK cells, thereby reversing the immunosuppressive TME. This strategy provides a gentle approach to TME remodeling without depleting CAFs and presents a promising tool for designing combination therapies for LC.^[^ ^382^ ^]^

In addition to directly targeting HSCs and CAFs, NMs can modulate other cells interacting with these stromal components to cooperatively suppress ECM deposition. Liver cancer stem cells (LCSCs) play a pivotal role in drug resistance, metastasis, and recurrence of LC, with their stemness maintained by CAFs.^[^ ^383^ ^]^ To target this unique microenvironment, Kong et al. developed a novel liposomal NM (CAP@CD133‐D/X‐Lip), which activates a CAP‐cleaving peptide upon encountering CAFs, leading to size reduction and exposure of a CD133 aptamer. This facilitates secondary targeting of LCSCs and the release of both DOX and the LCSC inhibitor XAV‐939 in deep tumor regions. The efficacy of CAP@CD133‐D/X‐Lip was validated in vitro and in vivo, confirming its ability to disrupt CAF–LCSC communication, thereby slowing tumor progression and improving therapeutic outcomes.^[^ ^384^ ^]^ NMs can also directly target the ECM itself by delivering agents that degrade ECM components. For instance, Xiao et al. showed that NMs carrying HAase effectively degraded ECM within the TME, enhancing CTL infiltration and proliferation. This strategy provides a promising therapeutic approach for tumors characterized by dense ECM structures.^[^ ^58^ ^]^

### 5.4 Modulating Metabolic Reprogramming

**Figure 11:**
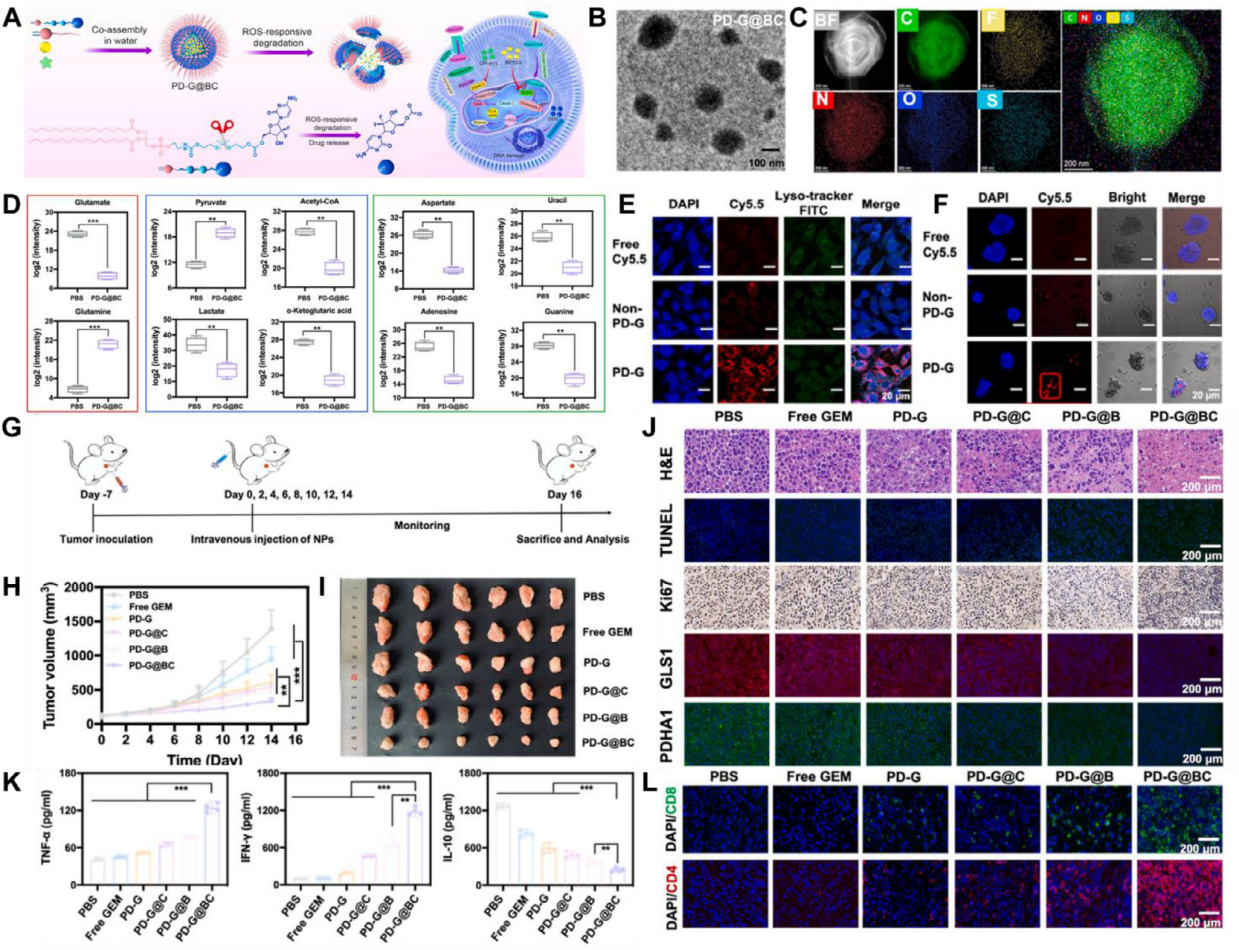
A) Schematic of the preparation of PD‐G@BC in tumors, its ROS‐mediated degradation, and the mechanism of BPTES and CPI‐613 combined inhibition of glycolysis and glutaminolysis in HepG2 cells. B) TEM images of PD‐G@BC NMs. C) Elemental mapping of PD‐G@BC NMs. D) Concentration levels of various metabolites in HepG2 cells. The red box indicates changes in glutamine metabolism, the blue box shows changes in TCA cycle metabolites, and the green box represents changes in amino acid synthesis metabolites. Metabolite levels were measured by liquid chromatography‐mass spectrometry, with results expressed as nmol/mg protein and mmol/g tumor cells. Data significance was determined by two‐sided Student's *t*‐test: **p < 0.01, ***p < 0.001. E) CLSM imaging of intracellular uptake of free Cy5.5 and Cy5.5‐labeled Non‐PD‐G or PD‐G in HepG2 cells. Lysosomes were stained with Lyso‐tracker FITC, and nuclei were stained with DAPI. F) CLSM images of intracellular uptake in HepG2 cell spheroids. G) Timeline of PD‐G@BC‐mediated metabolic immunotherapy in vivo. H) Tumor volume changes in mice over 14 days. I) Appearance of tumors excised after 14 days of treatment. J) Histological analysis of tumor tissue after 14 days of treatment, including H&E staining, TUNEL staining, Ki67 staining, anti‐GLS1 staining, and anti‐PDHA1 staining. Data are expressed as mean ± SD (*n* = 6), **p < 0.01, ***p < 0.001. K) ELISA detection of serum TNF‐α, IFN‐γ, and IL‐10 secretion levels (mean ± SD, **p < 0.01, ***p < 0.001). L) Immunofluorescent detection of CD8^+^ and CD4^+^ T cell infiltration in tumor tissues at the end of the anti‐tumor study (scale bar = 200 µm). Reproduced (Adapted) with permission.^[^ ^386^ ^]^ Copyright 2023, Elsevier.

To meet the demands of rapid proliferation, LC cells undergo metabolic reprogramming, involving significant alterations in pathways such as glucose, lipid, and amino acid metabolism. This reprogramming supports their growth and survival as well as promotes tumor progression and metastasis by remodeling the TME and regulating immune responses.^[^ ^29^ ^]^ These metabolic adaptations provide various therapeutic targets for novel strategies aimed at exploiting the metabolic vulnerabilities of LC cells. Regulating the metabolic reprogramming in LC cells has become a key area in the design of novel therapeutic strategies. Extensive studies have shown that certain metabolic pathways in drug‐resistant cancer cells are highly active, providing essential energy for cell activities and drug resistance. For example, enhanced glycolysis is often associated with reduced apoptosis. LC cells undergo glucose metabolic reprogramming to meet their abnormal proliferative needs, inadvertently leading to competition for available glucose with CTLs, thereby enabling tumor growth and immune evasion.^[^ ^385^ ^]^ In response to this challenge, Wang et al. developed ROS‐responsive NMs containing a thioketal‐based gemcitabine prodrug, co‐loaded with GLS1 inhibitor BPTES and PDHC inhibitor CPI‐613. This novel metabolic nanoregulator, termed PD‐G@BC, effectively disrupts glucose metabolism in tumor cells, depriving them of essential nutrients. Regarding immune response regulation, PD‐G@BC weakens the glucose supply to LC cells, thereby enhancing the metabolic activity of anti‐tumor immune cells, increasing the infiltration and function of immunogenic cells within the tumor, and alleviating immune suppression (**Figure** **11**).^[^ ^386^ ^]^

As the liver is the primary organ responsible for lipid metabolism, its dysfunction can lead to an imbalance in lipid synthesis, storage, and degradation, further promoting tumor cell proliferation, invasion, and drug resistance.^[^ ^387^ ^]^ However, the precise molecular mechanisms regulating lipid metabolism in LC remain poorly understood, particularly concerning the roles of key lipid‐metabolizing enzymes and their targeted therapeutic strategies, which require further investigation. A study explored key enzymes in lipid metabolism and their therapeutic application as NM‐targeted therapies. Through proteomic and single‐cell RNA sequencing data from patients with LC, the study identified high expression of long‐chain acyl‐CoA synthetase 3 (ACSL3) in LC cells, with ACSL3 overexpression positively correlating with abnormal lipid metabolism and poor prognosis. Based on this, an endosome pH‐responsive NM was developed for siACSL3 delivery, revealing effective inhibition of LC growth and metastasis.^[^ ^388^ ^]^

In studies targeting amino acid metabolism for NM‐based therapies, significant attention has been given to GSH metabolism. Proliferative tumor cells have an increased demand for GSH, and its breakdown, synthesis, and transport are critical for LC cell survival and development. Consequently, glutamine‐based smart NMs have been proposed as a potential cancer treatment strategy.^[^ ^389^, ^390^ ^]^ One study introduced an acid‐degradable tumor‐targeted nanosheet, Cu‐Hemin‐PEG‐LA (Cu‐Hemin‐PEG‐LA), in HepG2 cells. Cu‐Hemin‐PEG‐LA induces ferroptosis by depleting intracellular GSH and increasing intracellular Fe2^+^ levels, revealing superior anti‐tumor efficacy in vivo.^[^ ^391^ ^]^ Although the unique urea cycle and associated key metabolic enzymes of the liver are inextricably linked to LC development, studies on NM regulation of the urea cycle are limited.^[^ ^392^ ^]^ This represents a promising future direction. Branched‐chain amino acid metabolism plays an important role in tumor progression. Liu et al. identified a pyruvate metabolism enzyme, dihydrolipoamide S‐acetyltransferase (DLAT), which acetylates the K109 residue of AU RNA‐binding methylglutamate CoA hydrolase (AUH), inhibiting its activity and leading to leucine accumulation. Notably, upregulation of DLAT is associated with poor prognosis in patients with LC. Consequently, the authors developed an AUH K109R‐mRNA LNP therapy, effectively inhibiting tumor growth by restoring leucine catabolism.^[^ ^393^ ^]^

LC cells also reprogram other metabolic pathways, including the pentose phosphate pathway, one‐carbon cycle, and nucleotide metabolism. These pathways are crucial for supporting the rapid proliferation of LC cells and significantly impact the stability of the TME. These metabolic changes do not occur in isolation; instead, they are intricately interconnected to form a comprehensive network that meets the extensive biosynthesis and energy demands of LC cells.^[^ ^385^ ^]^ Targeting these metabolic networks using NMs for precise delivery, alongside immune modulation, is a highly effective combinatory therapeutic strategy.

### 5.5 Inducing ICD

**Figure 12:**
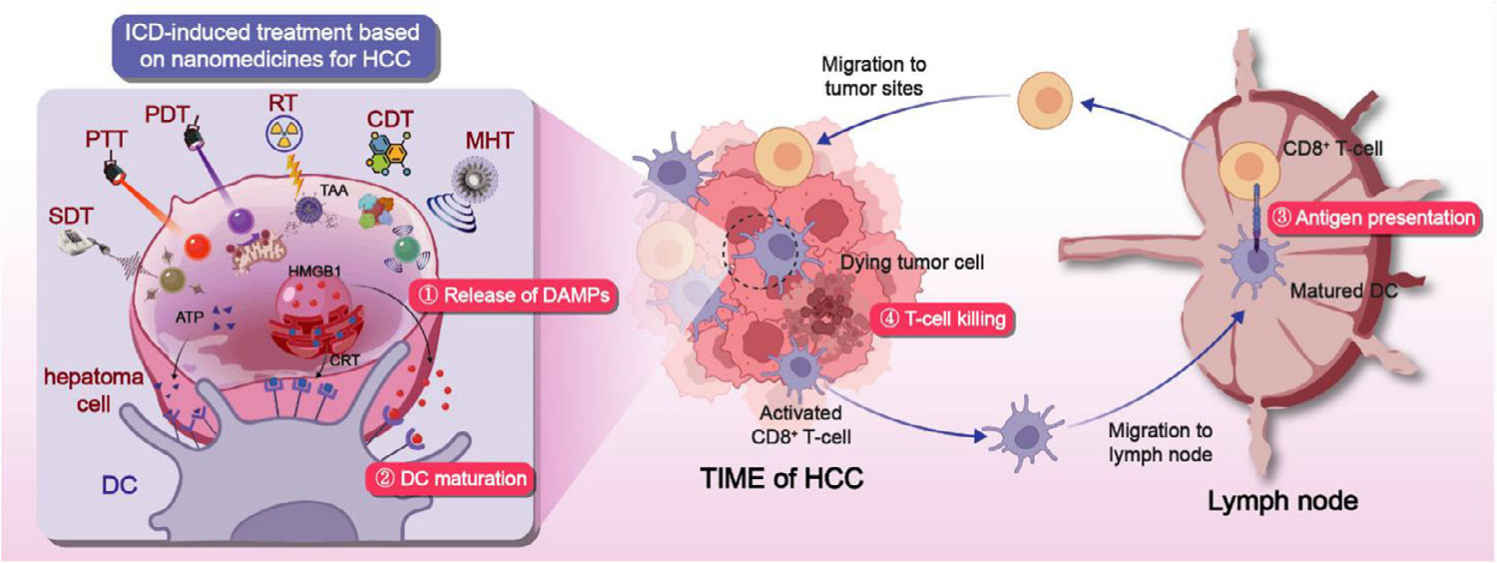
NMs induce ICD to reverse the immunosuppressive TME in LC. NMs, when combined with immunotherapy and other treatments (including PTT, PDT, SDT, MHT, CDT, and RT), induce ICD‐mediated antitumor effects, enabling multimodal synergistic treatment of LC.

Based on the mechanism of LC cell ICD, TAAs released by dying cells can form personalized vaccines in situ. This process activates DCs, promoting their maturation, differentiation, and antigen‐presenting functions, thereby driving a potent CD8^+^ T lymphocyte immune response.^[^ ^394^ ^]^ Simultaneously, DAMPs are co‐released, acting as immunoadjuvants to significantly enhance antigen recognition and presentation efficiency.^[^ ^395^ ^]^ This type of in situ vaccine system, characterized by a short preparation period and high clinical response rate, has become an important research focus in LC synergistic immunotherapy. Recent studies have confirmed that PTT, PDT, SDT, MHT, CDT, and RT can induce ICD for LC treatment, as shown in **Table** **2**.^[^ ^396^, ^397^ ^]^ NMs can respond to external energy stimuli, releasing therapeutic agents, while integrating these stimuli with immunotherapy to conduct multi‐modal synergistic treatment, thus reversing the immune‐suppressive TME in LC (**Figure** **12**).

**Table 2:** Common methods for inducing ICD with NMs.

Treatment	NMs	Therapeutic agents	Triggering conditions	Mechanisms	LC model	Ref.
PTT	GSBVVP	Gold nanorods, PD‐L1 inhibitor	NIR‐II light at 1208 nm	NIR‐II activates GSBVVP for hyperthermia, inducing ICD and promoting DC maturation, synergizing with anti‐PD‐L1 to enhance CD8^+^ T cell immunity.	Subcutaneous	^[^ ^402^ ^]^
	BPSP	Sorafenib, αPD‐L1 mAb	NIR‐II light at 1064 nm	NIR‐II activates BPSP for hyperthermia, triggering CRT exposure and DC maturation via DAMPs signaling.	Subcutaneous Orthotopic	[403]
	Apt/PPII/IR780‐NMs	Polyphyllin II (PPII), photosensitizer IR780	NIR light at 808 nm	PPII induces pyroptosis, synergized with phototherapy to release DAMPs, enhancing DC maturation and CD8^+^ T cell infiltration.	Subcutaneous	^[^ ^423^ ^]^
	Melanin@PLGA/Nuciferine (MPN)	Melanin, Nuciferine	NIR light at 808 nm	NIR activates MPN for hyperthermia and controlled nuciferine release, enhancing photothermal ablation and suppressing LC progression.	Subcutaneous	[424]
PDT	CIC‐Gal	Cisplatin, ICG	NIR light at 808 nm	CIC‐Gal targets SR‐BI receptor, PDT induces pyroptosis, releasing DAMPs to promote DC maturation.	Subcutaneous	[407]
	CTEP	CTEP	NIR‐III light at 1550 nm	CTEP targets via homologous recognition, membrane‐anchored PDT activates pyroptosis, releasing DAMPs for DC maturation.	Orthotopic	^[^ ^408^ ^]^
	mitochondria‐targeting enhanced NM(NZ@TG)	ZnPcC4, TH302	NIR light at 808 nm	NZ@TG targets mitochondria via GA, NIR‐PDT induces oxidative damage and apoptosis, releasing DAMPs for DC maturation.	Subcutaneous Orthotopic	[425]
	Spherical nucleic acids (SNAs)	Ce6, liver‐specific miR122	NIR light at 660 nm	SNA targets SR‐BI receptor, PDT induces ER stress releasing DAMPs for DC maturation, synergized with Met and miR122 to reverse immunosuppressive TME.	Subcutaneous	^[^ ^426^ ^]^
SDT	RGD@Ce6@MSA‐2@Liposome	Ce6, MSA‐2	ultrasound (1 MHz, 1 W cm^−2^, 50% duty cycle)	RCM‐Lip co‐delivers Ce6 and MSA‐2, combining SDT and STING pathway activation to enhance anti‐tumor immune response.	Subcutaneous	^[^ ^412^ ^]^
	IR780@FOM‐cRGD	Sonosensitizer IR780	ultrasound (1 MHz)	IR780@FOM‐cRGD enables ultrasound‐responsive delivery, combining SDT and immune checkpoint blockade.	Subcutaneous	[413]
	195/Ce6‐NBs	Ce6, miR‐195	ultrasound (1 MHz, 1.5W/cm2)	195/Ce6‐NBs co‐deliver Ce6 and miR‐195, integrating SDT and gene regulation.	Subcutaneous	^[^ ^427^ ^]^
	Mn‐GMSs	Sonosensitizer MnWOx	ultrasound (40 kHz, 3 W/cm2, 2 min)	Mn‐GMSs target delivery of TAE, integrating SDT, embolization, and metal‐induced immunomodulation to remodel the immune microenvironment	Subcutaneous Orthotopic	^[^ ^428^ ^]^
MHT	MSCH	Zn‐CoFe_2_O_4_@Zn‐MnFe_2_O_4_	AMF (1.7 mT)	MSCH targets CD44 receptor, acidic TME‐triggered Ca^2^⁺ release polarizes TAMs to the M1 phenotype, synergized with Ca^2^⁺ overload and MHT to induce ICD.	Orthotopic	[417]
	NLG919/PI‐FVIOs	NLG919, PI‐FVIOs	AMF (300 Oe, 360 kHz, 10 min)	Thermal release of NLG919 counteracts IDO‐mediated immune suppression, synergizing with MHT to enhance ICD.	Subcutaneous	^[^ ^429^ ^]^
	Zn‐CoFe_2_O_4_@Zn‐MnFe_2_O_4_ (ZCMF)‐aVEGF	ZCMF, aVEGF	AMF(1.7 mT, 20min)​	AMF activates ZCMF for mild hyperthermia, inducing NK cell activation and ICD to promote antitumor immunity.	Subcutaneous	^[^ ^430^ ^]^
	FVIO‐DHCA	FVIO	AMF (300 Oe, 360 kHz, 10 min)	Lysosome‐targeted magnetic hyperthermia induces lysosomal membrane permeabilization, activating Bid/caspase‐1 pathway to enhance ICD.	Subcutaneous	[414]
CDT	UCCu^2^⁺ NMs	Cu^2^⁺	pH 5.5	UCCu^2^⁺ NMs disintegrate in TME via pH/GSH/H_2_O_2_ response, releasing Cu^2^⁺, which consumes GSH via Fenton‐like reaction, generating ROS for apoptosis/necrosis.	Subcutaneous	^[^ ^418^ ^]^
	USFe^3^⁺LA NMs	Fe^3^⁺	pH 5.0	USFe^3^⁺LA NMs accumulate via EPR effect releasing Fe^3^⁺, consumes GSH generating ROS, inducing CRT/HMGB1/ATP release for DC maturation.	Subcutaneous	^[^ ^419^ ^]^
	PM‐FM/C	FeMoO_4_, CORM‐401	ultrasound (1 MHz, 1.25W/cm2)	Ultrasound triggers FM for ·OH production via CDT depleting GSH, ROS activate CORM‐401 releasing CO disrupting mitochondria, inducing lipid peroxidation and DAMPs release.	Subcutaneous Orthotopic	^[^ ^431^ ^]^
RT	UiO‐66‐Hf(2OH)‐C/B@HA	CB‐839 (GLS1 Inhibitor), BSO (γ‐GCS Inhibitor)	X‐ray (6 Gy, 1 Gy/min)	UiO‐66‐Hf targets via HA, X‐ray RT generates ROS, synergized with glutamine metabolism inhibition to deplete GSH and induce ICD.	Subcutaneous Orthotopic	[422]
	RKT@gel, Ta NMs	Ta NMs	X‐ray (5 Gy)	RKT@gel targets via HA coating, radiation‐triggered ROS causes DNA damage and mitochondrial apoptosis, inducing ICD.	Subcutaneous Orthotopic	[432]

PTT induces ICD by damaging DNA, denaturing proteins, and disrupting cell membranes, thereby releasing TAAs.^[^ ^398^ ^]^ However, because of the limited tissue penetration of laser sources, the immune activation effect is suboptimal, meaning that PTT alone cannot eradicate distant or metastatic tumors. NMs, acting as carriers or even as photothermal agents themselves, can target tumor areas for localized heating without damaging normal cells, thereby enhancing photothermal conversion efficiency and the anti‐tumor immune response.^[^ ^399^, ^400^, ^401^ ^]^ Dai et al. developed a multifunctional NMs, GSBVVP, by integrating gold nanorods with mesoporous silica core–shell nanoparticles, loading the PD‐L1 small‐molecule inhibitor BMS1166, and modifying the surface with a VEGF peptide vaccine (VVP). During LC progression, the application of GSBVVP combined with NIR‐II laser irradiation effectively induced photothermal ablation of tumor cells, triggered ICD, and significantly delayed tumor growth and progression. Meanwhile, the system enhanced CD8⁺ T‐cell infiltration within the TME, reduced the proportion of Tregs, and promoted DCs maturation. Through the synergistic effect of PTT and dual PD‐L1/VEGF blockade, GSBVVP remodeled the immunosuppressive TME, reversed T‐cell exhaustion and angiogenic signaling pathways, thereby suppressing the growth, metastasis, and recurrence of LC, and providing long‐term antitumor protection via the induction of immune memory responses.^[^ ^402^ ^]^ The combined use of PTT and ICB shows strong therapeutic potential. Yang et al. constructed a self‐delivering, photothermal‐boosted NanoBike (BPSP), which consists of black phosphorus (BP)‐conjugated anti‐PD‐L1 monoclonal antibodies (mAb) and Sor. The mechanism involves anti‐PD‐L1 mAb blocking PD‐1/PD‐L1 interactions, whereas PTT‐induced ICD activates effector T cells and enhances the response to PD‐L1 mAb. The highly encapsulated BPSP transformed “cold” tumors into “hot” tumors, improving the CTL/Treg ratio, and cured orthotopic LC in mice.^[^ ^403^ ^]^

PDT activates photosensitizers to generate ROS, further inducing ICD and antigen presentation.^[^ ^404^ ^]^ However, similar to PTT, PDT therapeutic efficacy is limited by tissue penetration and the hypoxic TME.^[^ ^405^ ^]^ Increasing evidence shows that chemotherapeutic drugs and natural products can induce ICD or generate ROS, which is why PDT is often combined with CDT to more effectively activate immune responses.^[^ ^406^ ^]^ Wang et al. designed polysaccharide‐based nanomissiles (CIC‐Gal) loaded with cisplatin (CDDP) and ICG, which accumulate effectively in tumors. By combining CDT/PDT, these NMs exhibited potent anti‐tumor effects against LC. Importantly, CIC‐Gal NMs protect cisplatin from GSH‐mediated detoxification, while promoting CD8^+^ T cell infiltration, inhibiting the proliferation and migration of MDSCs, and extending the survival of tumor‐bearing mice by >40 d. This strategy showcases remarkable TME reprogramming ability.^[^ ^407^ ^]^ PDT combined with ICB treatment synergistically enhances the immune response. One study reported a smart NM based on acid‐etched layered double hydroxide nanosheets (E‐LDH) with H2O2/pH dual gatekeepers (CTEP), targeting LC for membrane‐anchored PDT and triggering strong pyroptosis to enhance anti‐tumor immunity. The system, when combined with anti‐PD‐L1 therapy, reduces side effects and activates adaptive immunity, inhibiting both primary and metastatic LC progression.^[^ ^408^ ^]^

Because of the non‐invasive nature and excellent penetration depth, and treatment precision of SDT, ultrasound stimulation as a response trigger for drug delivery systems has gained broad recognition. SDT‐induced ICD, improving the immune‐suppressive TME has also been extensively reported.^[^ ^409^, ^410^, ^411^ ^]^ Yang et al. synthesized an NM sensitizer RGD@Ce6@MSA‐2@Liposome (RCM‐Lip), which triggers ICD through SDT and activates cGAS‐STING signaling through MSA‐2. The application of RCM‐Lip significantly increased the proportion of mature DCs and CD8^+^ T cell infiltration in the TME and reduced immunosuppressive Tregs. Through synergistic SDT‐induced antigen release and STING pathway activation, RCM‐Lip reshaped the TME, increasing in vivo tumor inhibition rate to 73.5%, providing a novel strategy for LC precision immunotherapy.^[^ ^412^ ^]^ In SDT combined with ICB therapy, a study reported iron‐based micelle NMs (loaded with IR‐780 dye and surface‐modified with cyclic RGD) that activate ICD through ultrasound and combine with anti‐PD‐L1 checkpoint blockade. Results showed that these NMs increased CD8^+^ T cell infiltration 3.2‐fold and CD80/CD86 expression on DCs 2.5‐fold, improving the response rate of anti‐PD‐L1 monotherapy from 22% to 67%.^[^ ^413^ ^]^

MHT mediated by MNMs has emerged as a promising approach for tumor relief.^[^ ^414^ ^]^ However, common MNMs suffer from low magnetic heating efficiency and insufficient tumor accumulation, limiting their application in ICD induction. MHT is often used as an adjunct in ICD induction.^[^ ^415^, ^416^ ^]^ For example, a HA‐modified CaP‐coated MNM (MSCH), was used in mild MHT and calcium ion regulation strategies to reshape the immune‐suppressive TME in LC, activating various immune cells. Specifically, the acidic TME and mild MHT effectively promote calcium ion release from MSCH, leading to calcium overload in LC cells, which synergistically activate ICD and induce a potent adaptive immune response, revealing excellent therapeutic effects in treating orthotopic LC.^[^ ^417^ ^]^

CDT primarily operates through Fenton reactions or Fenton‐like reactions, catalyzing the production of ROS, especially hydroxyl radicals (·OH), from H2O2 in the TME, thereby inducing tumor cell death.^[^ ^406^ ^]^ NMs as drug delivery systems can target CDT drugs to tumor sites, often in combination with other therapies, such as PTT/PDT, to achieve multi‐modal synergistic treatment. Lai et al. proposed hybrid NMs (UCCu2^+^ NMs) assembled from photosensitizers (PSs), carbon dots (CDs), chemotherapeutic drug UA, and copper ions (Cu2^+^) to achieve synergistic cancer therapy. Upon tumor tissue entry, the PDT and fluorescence functions of UCCu^2+^ NMs are restored under TME stimulation. Moreover, Cu^2+^ reacts with H2O2 and depletes GSH in cancer cells through redox reactions, generating additional CDT capacity, thereby amplifying oxidative stress and enhancing treatment effects because of ROS therapy. This multi‐pronged approach amplifies therapeutic outcomes.^[^ ^418^ ^]^ In another study exploring the combination of CDT and ICB, it was confirmed that CDT‐induced ICD increases CTL infiltration, enhancing immune responses against PD‐L1.^[^ ^419^ ^]^

RT serves as a cornerstone of cancer therapy and induces ICD, which highlights the development of combined immunotherapeutic strategies.^[^ ^420^ ^]^ Clinical practice has shown that although some patients benefit significantly from combined RT treatment, the heterogeneity of the TME leads to radiation resistance in a subset of cases, directly limiting the clinical benefit.^[^ ^421^ ^]^ To address this challenge, NMs have been used in studies to enhance tumor sensitivity to RT. Zhang et al. developed a hafnium‐based metal‐organic framework (MOF) NM (UiO‐66‐Hf(2OH)‐C/B@HA), which concurrently inhibits glutamine metabolism and enhances radiosensitization, yielding synergistic antitumor effects. In a MYC‐amplified LC model, this NM remodeled the TME, markedly promoted DC maturation (CD86⁺ DCs reached 94.63%), increased granzyme B⁺ T cell infiltration by 3.5‐fold, and reduced the RT dose to 6 Gy. Mechanistic studies have shown that the system increased ROS production by 41%, promoted ICD as evidenced by a 2.8‐fold increase in calreticulin expression, and worked in synergy with a PD‐1 inhibitor, leading to a 78% reduction in tumor volume in an orthotopic LC model, significantly outperforming monotherapy treatments.^[^ ^422^ ^]^

### 5.6 Modulating the Microbiota and Its Metabolites

In recent years, smart NMs have shown unique potential in modulating gut microbiota–host interactions to inhibit LC progression and reshape the TME. Studies have revealed that gut dysbiosis can induce chronic liver inflammation, metabolic reprogramming, and immunosuppression through the gut–liver axis, thereby promoting LC development.^[^ ^433^ ^]^ Current NM strategies are primarily focused on regulating gut microbiota homeostasis and their derived metabolites. To reverse liver inflammation and immunosuppressive TME induced by gut microbiota‐derived LPS, Yao et al. proposed a Trojan‐horse strategy using an orally administered dextran‐carbenoxolone (DEX‐CBX) conjugate, which combines prebiotic functionality with a glycyrrhetinic acid analogue. This design enables glycyrrhetinic acid to be specifically delivered to LC tissue through the gut–liver axis, effectively regulating both liver inflammation and gut microbiota composition. In an orthotopic HCC model, DEX‐CBX treatment resulted in a 45–95% reduction in LPS‐associated microbiota, particularly Helicobacter, compared to phosphate‐buffered saline (PBS) controls. Moreover, DEX‐CBX significantly increased the infiltration of natural killer T cells (5.7‐fold) and CD8^+^ T cells (3.9‐fold), while reducing M2 macrophages by 59%, leading to a tumor inhibition rate of 85.4%.^[^ ^434^ ^]^ This study provides a novel approach to counteract LC immunosuppression caused by gut microbiota‐derived metabolites.

Not all microbial metabolites lead to immunosuppression. Interestingly, one study identified a gut‐derived metabolite that promotes the polarization of TAMs into an immune‐stimulatory phenotype, enabling targeted therapy through an optimized delivery system. Han et al. discovered that d‐lactic acid (DL), a microbial metabolite, promotes the reprogramming of M2 phenotype into M1 phenotype. They developed DL‐loaded PLGA NPs (DL@NP), encapsulated in LC cell membranes modified with M2 macrophage‐targeting peptides (DL@NP‐M‐M2pep), to ensure precise delivery. Following intravenous administration, DL@NP‐M‐M2pep successfully targeted M2 macrophages in LC tissue, inducing M1 polarization. When combined with CD47 antibody treatment, the therapeutic effect was significantly enhanced.^[^ ^95^ ^]^ Despite these advances, NM designs targeting gut microbiota and their metabolites remain limited, and further studies are required to validate the safety and immunomodulatory efficacy of this strategy.

## 6 Types of NMs for LC Immunomodulation

Currently, the primary types of NMs used in LC‐targeted drug delivery for immune regulation include inorganic NMs, carbon NMs, lipid NMs, polymeric NMs, hydrogels, and exosomes (Figure 7).^[^ ^435^ ^]^ **Table** **3** lists typical application scenarios and mechanisms of various types of NMs.

**Table 3:** Summary of NMs for immune modulation.

Class	Typical examples	Size and shape	Treatment modalities	Refs.
Metal NMs	bPEI/AuNPs	<90 nm, spherical	Intracellular delivery of anticancer siRNA targeting c‐Myc gene in Huh7 LC cells	^[^ ^436^ ^]^
	CAL@PG	CAL@PG: 270–300 nm, spherical	PTT/CDT/Chemotherapy	^[^ ^437^ ^]^
Metal oxide NMs	FVIO@PDA	72‐109 nm, Hollow ring‐like structure	Len/MHT	^[^ ^438^ ^]^
	ZnO‐CuO (50:50)	18.6nm, Hollow ring‐like structure	Cell cycle intervention/Apoptosis/Necrosis/ Autophagy/Anti‐migration	^[^ ^439^ ^]^
MOFs	Sor@Fe‐MOF	139.9 ± 3.1 nm, spherical	Sor/Ferroptosis induction/ Immune TME Remodeling	^[^ ^440^ ^]^
	MOF‐CpG‐DMXAA	150nm, Uniform cube/ spherical	Reprogramming TAMs/ Promoting DCs maturation/ Vascular disruption/ Immune synergy	^[^ ^351^ ^]^
Silicon NMs	ZnAs@SiO_2_	35 ± 2 nm, spherical	Inhibition of Stemness/ Suppression of EMT/ Regulation of SHP‐1/JAK2/STAT3 Pathway/ Apoptosis	^[^ ^441^ ^]^
	USMNs‐CL	191.6 ± 6.6 nm, spherical mesoporous structure	Synergistic Co‐delivery (Sor/UA)/ Anti‐metastasis/ Apoptosis	^[^ ^442^ ^]^
CNTs	DF‐MWCNT	210.9 ± 2.8 nm, nanotubes	Synergistic Co‐delivery (DGN/FUA)/Apoptosis/ Upregulation of Tumor‐Suppressive miRNAs	^[^ ^443^ ^]^
	Durvalumab/CNT/PEI/aptamer‐siRNA chimeric NMs	Diameter: 20–30 nm, Length: 200–350 nm, nanotubes	Durvalumab/ Targeted Delivery & Gene Silencing (Trem2 siRNA)/ Synergistic Anti‐tumor Effect	^[^ ^444^ ^]^
CDs	Ber‐CDs	11.5 nm, spherical	Selective Cytotoxicity/ Dual‐Modal Imaging Guidance	^[^ ^445^ ^]^
	TC‐WS‐CQDs	1.4 nm, spherical	PDT/ fluorescence imaging‐guided tumor targeting therapy/ ​Activation of p53‐AMPK signaling pathway	^[^ ^446^ ^]^
Graphene	PEG‐GO‐PEI‐MAN@LDN193189	178.23 nm, stereoscopic nanostructure	Dual Targeting Accumulation/ CSC Stemness Suppression/ TME Remodeling/ Synergistic Antitumor Effects	^[^ ^447^ ^]^
	MGO‐TCA‐FA@DOX	13 ± 2 nm, irregular spherical	Chemotherapy/ PTT/ Dual Targeting	^[^ ^448^ ^]^
Liposomes	CAR&Siglec‐GΔITIMs LNP	86.93 nm, spherical	Enhanced Tumor Phagocytosis by CAR‐Ms/ Blockade of CD24 “Don't Eat Me” Signal by Siglec‐GΔITIMs/ NIR In Vivo Imaging Monitoring	^[^ ^449^ ^]^
	LA‐CMGL	160–170 nm, spherical	Targeted Delivery/ Chemotherapy/ MRI Visibility/ miR‐145 suppresses the SENP1/HK2 glycolysis pathway	^[^ ^450^ ^]^
PEG/PLGA	LFC131‐PLGA‐PEG.Sora/Meta	145–147 nm, spherical	Targeted Delivery/ Sor/ metapristone reduced CXCR4 expression	^[^ ^451^ ^]^
	PLGA‐PEG‐AEAA.SCU	135 nm, spherical	SCU induced ICD/ Targeted Delivery/ Immune Activation	[452]
Polysaccharides	Dox/FA‐BSP‐SA	129–145 nm, spherical	Chemotherapy/ Targeted Delivery	[453]
	GACS‐Cur@RBCm	171.6 nm, spherical	Liver Targeting/ Cur induces tumor cell apoptosis/ Downregulates Ki‐67 and VEGF expression/ Immunomodulation	[454]
CS	CC@SR&SF@PP	50.49±5.34 nm, spherical	Sor/ pH‐Triggered Release/ T‐cell immunoglobulin mucin‐3 (Tim‐3) siRNA silences immune checkpoint	[455]
	CMCNP‐GL	100‐205 nm, square flake‐shaped	Active Targeting/ Biphasic Drug Release/ PTX	[456]
Hydrogels	C_16_‐N/T Hydrogels	100‐205 nm, Nanofiber Structure	Sustained Release/ Liver Targeting/ Selective Cellular Uptake	[457]
	PLDX‐PMI	–	Anti‐angiogenesis/ Inhibition of endothelial cell migration and proliferation/ Macrophage repolarization/ Cytokine modulation	[458]
Exosomes	NK‐exos‐Dox	183.5 ± 54.12 nm, spherical	Enhanced cellular uptake/ Upregulates pro‐apoptotic proteins/ Synergistic cytotoxicity/ No significant systemic toxicity	[459]
	ExosomeRNP	50–200 nm, Saucer‐shaped nanovesicles	Liver‐specific targeting/ Efficient cytosolic delivery Genome editing therapy/ Low immunogenicity	[460]
	BMDC_TEX‐N1ND_	100–150 nm, Saucer‐shaped nanovesicles	Enhanced DC immunogenicity/ Long‐lasting T cell immunity/ Reshaped tumor microenvironment	[461]
Nanovaccines	TRZ	200.7±3.8 nm (TZ NPs), 201.9±4.5 nm (RZ NPs), Irregular shape	In situ generation of nanovaccine in hypoxic tumor environment for enhanced cytotoxic T cell infiltration and abscopal effects	[462]
	pDNA‐NPs	100.8 ± 19.2 nm, spherical	Spleen‐targeted delivery of neoantigen DNA vaccines via RBC hitchhiking for LC immunotherapy	[463]
	RNA LNPs	209.68±6.14 nm, spherical	Intracellular delivery of total tumor RNA for DCs activation and CTLs induction in LC immunotherapy	[336]

### 6.1 Inorganic NMs

Typical examples of inorganic NMs include metals, metal oxides, magnetic NMs, and silicon NMs. Although most of these NMs are not biodegradable, their advantages lie in the ease of size control and surface modification, making them promising candidates for drug delivery systems.

#### 6.1.1 Metal‐Containing Inorganic NMs

Metal NMs, with their high surface‐area‐to‐volume ratio, can easily conjugate with therapeutic molecules and/or targeting ligands to cancer sites. Owing to their EPR effect, they can cross physiological barriers and accumulate passively at tumor sites.^[^ ^464^ ^]^ Gold, silver, and palladium NMs have exhibited both homogeneous and heterogeneous catalytic activities. The shape, size, and coating of NMs are critical parameters influencing catalytic performance as they affect the interactions between particles and the changes in their surface area.^[^ ^435^ ^]^ Among these metal nanoparticles, AuNPs are the most attractive option. Coupling AuNPs with drug molecules helps improve drug efficacy by regulating delivery rates and release to target tissues. Numerous studies have shown that AuNPs can deliver a variety of molecules, vaccines, recombinant proteins, or nucleotides to their targets. Furthermore, surface modifications of AuNPs can alter their properties for multiple cancer treatment purposes.^[^ ^465^ ^]^ One study showed that AuNPs modified with branched PEI (bPEI) may be an excellent option for delivering siRNA to LC cells. siRNA targeting the oncogene c‐Myc was designed and conjugated with bPEI/AuNPs. After transfecting HuH7 cells with the siRNA/bPEI/AuNP complexes, c‐Myc expression was quantified using real‐time polymerase chain reaction (PCR). Their results showed that siRNA effectively silenced the c‐Myc gene.^[^ ^436^ ^]^ In addition, some metal NMs possess excellent PTT and PDT properties and benefit from their excellent drug delivery capabilities, which can synergistically enhance LC treatment. Xu et al., to overcome the low bioavailability and limited efficacy of Len, uniformly encapsulated ultrasmall copper sulfide nanocrystals (Cu_2‐x_S NCs) and ultrasmall AuNPs into mannose‐amino conjugated PLGA through a nanoprecipitation method, creating drug delivery NMs (CAL@PG). CAL@PG exhibits excellent stability under physiological conditions and rapidly releases Len under the unique TME and high temperatures, owing to the NIR‐II PTT effect of CAL@PG. Furthermore, the elevated temperature, regenerated H_2_O_2_, and the acidic pH of the TME significantly enhance the copper‐based Fenton‐like chemical reaction, enriching the multimodal synergistic LC therapy with enhanced efficacy.^[^ ^437^ ^]^

Metal oxide NMs have been widely used in various applications such as sensing and catalysis, and they hold great potential in LC therapy owing to their unique properties. These NMs can serve as therapeutic agents themselves, carry other therapeutic agents as ligands, and some even possess magnetic properties that enable both treatment and imaging monitoring.^[^ ^466^ ^]^ Iron oxide NMs are a favored metal oxide NM, as they exhibit drug delivery capabilities in addition to excellent magnetic properties, granting them potential for imaging and MHT. These applications, combined with the typical carrier capabilities of iron oxide NMs, make them an attractive material for in vivo LC therapy. Maeng et al. revealed the dual potential of iron oxide NMs in a nanosystem incorporating DOX as the therapeutic agent and folate as a targeting ligand. The NMs were able to reduce tumor size in rat livers more significantly than DOX alone. In addition, the position of the drug delivery system could be tracked using MRI.^[^ ^467^ ^]^ Low immunogenicity of LC and high toxicity of Len often compromise the efficacy of immunotherapy. To address these clinical challenges, Ye et al. designed a PDA‐coated ferromagnetic vortex domain iron oxide nanoring (FVIO@PDA) that maximized the drug carrier and magnetic heat conversion capabilities of iron oxide NMs. Under the heat generated by AMF, Len was released in a controlled and synergistic manner. In subcutaneous Hepa1‐6 LC models, FVIO@PDA‐loaded Len significantly increased CTL levels by ≈3.86‐fold compared to those of the control group. This combination of Len and MHT also reduced Treg levels to 1.4%, triggering a potent antitumor immune response. The dual advantages of iron oxide NMs address clinical issues in LC immunotherapy.^[^ ^438^ ^]^ However, iron oxide NMs still have unresolved limitations, primarily related to biocompatibility and in vivo clearance. In addition to iron oxide NMs, other types of metal oxide NMs, such as zinc oxide (ZnO), alumina (Al_2_O_3_), and copper oxide (CuO) NMs, are also being explored for LC therapy. Allam et al. synthesized ZnO and CuO nanocomposites in different ratios and evaluated their cytotoxic effects on various LC cell lines. The results showed that nanocomposites with equal amounts of ZnO and CuO exhibited the highest cytotoxic activity against the HuH7 cell line, with no adverse effects on normal rat liver epithelial cells. This study provides valuable insights for future LC therapies using dual‐metal NMs.^[^ ^439^ ^]^ Metal oxide NMs, with their carrier, therapeutic, and imaging functionalities, present promising prospects. The issue of in vivo clearance could potentially be addressed by optimizing the size and charge of the NMs, a crucial consideration for enhancing biocompatibility.^[^ ^468^ ^]^

MOFs are rapidly developing coordination polymers with a three‐dimensional (3D) porous structure, representing a class of important porous materials. These materials possess tunable pore sizes and high specific surface areas, making them highly suitable for drug delivery systems owing to their strong drug‐loading capacity and ease of modification.^[^ ^469^ ^]^ Because of their excellent drug‐loading capability, Yan et al. developed an Fe(III)‐based MOF NM (Fe‐MOF) with ferroptosis and immune activation functionalities for encapsulating Sor (Sor@Fe‐MOF), thereby effectively enhancing ferroptosis and remodeling the TME.^[^ ^440^ ^]^ In reversing immune‐suppressive TME in LC, MOFs provide a promising solution through their precise delivery. Chen et al. reported a NM (MOF‐CpG‐DMXAA) formed by the self‐assembly of CpG ODN and the vascular‐disrupting agent 5,6‐dimethylxanthenone‐4‐acetic acid (DMXAA) STING agonist with MOF‐801. MOF‐CpG‐DMXAA effectively delivers CpG ODNs and DMXAA to cells, reprogramming TAMs, promoting DC maturation, and disrupting tumor vasculature, thereby synergistically improving the TME. In a mouse model of LC, MOF‐CpG‐DMXAA significantly enhanced the innate immune response by synergistically activating the cGAS‐STING‐NF‐κB signaling pathway, achieving an 80% tumor clearance rate and completely inhibiting tumor recurrence, providing a novel strategy for solid tumor immunotherapy.^[^ ^351^ ^]^

#### 6.1.2 Silicon NMs

By using sol‐gel chemistry and selecting different structural templates, various structural morphologies of Silicon NMs (SiNMs) can be synthesized. This approach enables the creation of MSNs with high surface area, excellent thermal stability, and mechanical strength, as well as hollow silica NMs characterized by low density, high surface area, and superior adsorption capacity. In addition, dendritic fibrous silica NMs are generated with efficient diffusion channels and accessible internal surfaces.^[^ ^294^ ^]^ Silicon dioxide (SiO_2_) NMs are commonly used as drug delivery carriers because of their biocompatibility. They gradually degrade in vivo to form silicates, which are excreted through the kidneys, minimizing long‐term toxicity risks. Huang et al. developed ZnAs@SiO_2_ NMs by encapsulating arsenic trioxide within SiO_2_ nanoparticles. The SiO_2_ shell encapsulates the zinc‐arsenic complex, preventing premature release of arsenic in the bloodstream and reducing toxicity to normal tissues. Notably, the SiO_2_ shell controls the slow release of arsenic, enabling sustained action within tumor cells, achieving a carrier‐protective layer‐drug release switch functionality.^[^ ^441^ ^]^ The use of NMs for co‐delivery of multiple drugs has emerged as a promising strategy to enhance cancer treatment efficacy. Zhao et al. developed pH‐sensitive MSN‐based NMs for co‐delivering multi‐targeted tyrosine kinase inhibitors, Sor and UA. These NMs are decorated with pH‐sensitive CS and lactobionic acid (USMNs‐CL), targeting cells overexpressing ASGPR. During LC therapy, USMNs‐CL significantly inhibited the proliferation, migration, and adhesion of SMMC‐7721 LC cells. Furthermore, through synergistic effects, it induced apoptosis (with a cell death rate of 70.49%) and downregulated EGFR/VEGFR2 protein expressions. The application of USMNs‐CL remodels the proliferative and angiogenic signaling pathways within the TME, reducing tumor volume to 32.86% of the control group in the H22 tumor‐bearing mouse model and effectively inhibiting LC lung metastasis. This strategy achieves both chemical prevention and immune regulation in LC therapy.^[^ ^442^ ^]^

### 6.2 Carbon NMs

Carbon NMs, owing to their exceptional physicochemical properties, unique structural characteristics, and biocompatibility, have emerged as highly promising drug delivery carriers for LC, providing various strategies for the design of NMs targeting LC. Common types of carbon‐based NMs include carbon nanotubes (CNTs), CDs, and graphene.

CNTs can be functionalized with targeting ligands and loaded with chemotherapy drugs or therapeutic agents, enabling direct drug delivery to cancer cells and reducing side effects. Extensive studies have shown that CNTs can precisely target LC cells.^[^ ^470^ ^]^ Ferulic acid (FUA) and diosgenin (DGN) were loaded into multi‐walled CNTs (DF‐MWCNTs) and coated with CS‐stearic acid (SA) to enhance drug loading and release kinetics. In vitro studies of DF‐MWCNTs showed effective drug release and inhibition of HepG2 LC cells, highlighting their potential as a natural drug delivery system.^[^ ^443^ ^]^ Qiang et al. developed durvalumab/CNT/PEI/aptamer‐siRNA chimeric NMs for LC immunotherapy. Both in vitro and in vivo studies showed that these NMs specifically bind to LC cells, downregulate Trem2 expression, and do not damage healthy liver and lung tissues.^[^ ^444^ ^]^ In addition, CNT‐Sor functionalized with Sor exhibited superior cytotoxicity against HepG2 cells compared to free sorafenib. In vivo studies indicated that CNT‐Sor outperformed free Sor in all evaluated endpoints, indicating greater potential for cancer suppression.

**Figure 13:**
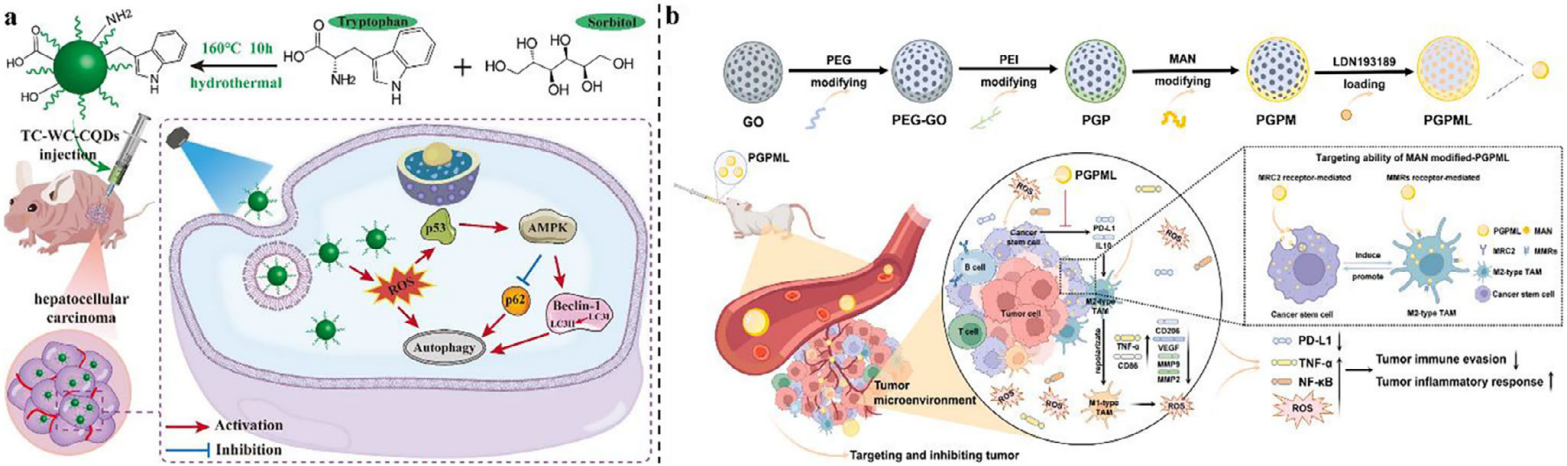
a) Schematic of the preparation of TC‐WS‐QCD and its mechanism for treating LC via the p53‐AMPK pathway. Reproduced (Adapted) with permission.^[^ ^446^ ^]^ Copyright 2022, Springer Nature. b) Synthesis pathway of the PGPML NMs and their tumor‐targeting and TME remodeling capabilities in LC. Reproduced (Adapted) with permission.^[^ ^447^ ^]^ Copyright 2025, Elsevier.

CDs, a relatively novel class of NMs, show significant potential in biological imaging, sensing, and drug delivery. This is because of their excellent optical properties, biocompatibility, and ease of surface modification. CDs, small carbon‐based NMs typically ranging 1–10 nm in size, are able to localize primarily in tumors, liver, and kidneys in mice, with the highest fluorescence intensity observed in LC.^[^ ^471^ ^]^ In one study, berberine, an anticancer molecule, was directly pyrolyzed to form CDs. These CDs were predominantly localized in the tumors and liver of mice and exhibited higher efficacy in vitro and in vivo than treatment with berberine alone, without significant side effects. This strategy is promising, as it allows the therapeutic agent to be targeted to the liver based on the size characteristics of the CDs, though further optimization is required for selective cancer cell targeting.^[^ ^445^ ^]^ Even though carbon‐based NMs are not typically considered highly active, they can be integrated with various organic or inorganic components to create useful drug delivery systems. The multifunctionality of CDs is further enhanced when incorporated into other nanocarrier systems. Wang et al. synthesized carbon quantum dots (CQDs) from naturally biocompatible tryptophan using a one‐pot hydrothermal method, producing trichrome‐tryptophan‐sorbitol CQDs (TC‐WS‐CQDs). Strong green fluorescence was detected in LC cells compared to normal liver cells, indicating the targeting ability of TC‐WS‐CQDs for LC cells. Furthermore, TC‐WS‐CQDs produced substantial ROS, inducing autophagy in LC cells. In both in vitro and in vivo studies, the green‐fluorescent TC‐WS‐CQDs induced autophagy through the p53‐AMPK pathway, revealing significant tumor suppression with minimal systemic toxicity. This highlights the potential of CDs in diagnostics, targeting, and therapy (**Figure** **13**a).^[^ ^446^ ^]^

Graphene possesses adhesive properties, and graphene oxide (GO) is renowned for its high drug‐loading capacity owing to its large surface area, which results from its shape and surface‐active functional groups. These functional groups also facilitate chemical modification, enabling multifunctional design. Qi et al. developed an NM (PGPML) by combining mannose‐modified GO with the LDN193189 inhibitor. During LC progression, PGPML was able to target CSCs through the MRC2 receptor and M2 phenotype TAMs through the MMR receptor, significantly downregulating stem cell markers CD133 and PD‐L1, promoting macrophage re‐polarization from M2 to M1, and enhancing TNF‐α levels. The use of PGPML remodeled the immunosuppressive TME by dual‐targeting CSCs and activating antitumor immunity. In this process, GO, as a multifunctional NM, integrated drug loading, targeted delivery, microenvironment modulation, and enhanced biocompatibility, ultimately significantly inhibiting LC progression through synergistic effects (Figure 13b).^[^ ^447^ ^]^

### 6.3 Lipid NMs

LNPs are versatile drug delivery systems composed of ionizable lipid‐based spherical vesicles. LNPs exhibit efficient endocytosis and endosomal escape capabilities, enabling them to accumulate in areas with high vascular permeability. They can carry both hydrophilic and hydrophobic compounds and possess minimal toxicity.^[^ ^49^ ^]^ LNPs can also prolong therapeutic effects, control drug release, and undergo surface modifications to enhance solubility or evade immune detection.^[^ ^472^ ^]^ NMs consist of four lipid components, with the ionizable lipid responsible for electrostatic binding with nucleic acids. The other three components each serve specific functions: cholesterol stabilizes the NM structure and enhances cellular uptake through low‐density lipoprotein receptor‐mediated endocytosis; PEGylated lipids improve the overall stability of LNPs and extend their circulation time in the bloodstream; and auxiliary lipids accelerate the structural transformation of NMs within cells and facilitate drug release.^[^ ^329^, ^472^, ^473^ ^]^ Currently, LNPs are primarily used to protect nucleic acid cargo from degradation and deliver it to the cytoplasm of cells. An LNP developed by Yang et al. (CAR&Siglec‐GΔITIMs LNP) enables the co‐delivery of two mRNAs. This LNP delivers two mRNAs to liver macrophages: one encoding a chimeric antigen receptor (CAR) targeting GPC3, which enhances macrophage phagocytosis of LC cells, and the other encoding a Siglec‐G CAR lacking ITIMs, which overcomes the “do not eat me” signal. The dual mRNA‐LNP system significantly improved the phagocytic activity of liver macrophages towards LC cells (with a tenfold increase in in vitro phagocytosis), stimulated antigen presentation, remodeled the TME, inhibited the growth of in situ LC, and prolonged survival (with a 40% survival rate by day 60).^[^ ^449^ ^]^

LNPs can serve as drug carriers, improving biosafety, and their modification with functional units allows for controlled drug release. Camptothecin (CPT), a commonly used chemotherapeutic agent for LC treatment, is limited in clinical application because of severe toxicity and acquired resistance. Combined chemotherapy‐gene therapy has been reported as an effective strategy to combat resistance and enhance cancer cell sensitivity to cytotoxic drugs. Rong et al. developed a targeted nano‐system (LA‐CMGL) that co‐delivers CPT and miR‐145 using LA‐modified LNPs. In a drug‐induced LC model, LA‐CMGL treatment significantly extended the survival of mice to 121 d, compared to <90 d in the control group, while inhibiting tumor proliferation and migration. Biosafety testing confirmed that LA‐CMGL caused almost no systemic side effects.^[^ ^450^ ^]^

### 6.4 Polymeric NMs

Polymeric NMs represent a promising approach for enhancing cancer therapy by improving drug delivery systems. Composed of natural or synthetic polymers, polymeric NMs can be formulated into nanocapsules or nanospheres and encapsulate drugs through conjugation, adsorption, or entrapment methods.^[^ ^474^ ^]^ In addition, polymeric NMs with core‐shell structures are effective in delivering hydrophobic drugs, while reducing the use of toxic solvents. These NMs can be developed through various manufacturing techniques and reveal their potential in LC therapy by enabling targeted drug delivery and enhancing bioavailability.^[^ ^475^ ^]^

PEG is a commonly used polymer, as it is frequently used in the medical field as a laxative, excipient, and for various chemical applications, including as a hydrophilic coating, a polar stationary phase, and protein linker. PEG shows practicality in drug delivery systems because of its ability to coat the surface of NMs and extend their circulation time in the bloodstream.^[^ ^476^ ^]^ In one study, long‐circulating PEG‐modified liposomes loaded with Sor (anti‐VEGFR‐LC‐PEG‐SOR‐NMs) were constructed to target LC. The NMs exhibited a uniform spherical structure with an average diameter of 119.8 ± 4.2 nm. Both in vitro and in vivo experiments showed effective tumor targeting and growth inhibition with minimal side effects, highlighting the potential of these NMs as tumor‐targeted NMs for LC treatment.^[^ ^477^ ^]^

Owing to their biocompatibility and biodegradability, PLGA has been approved by the FDA for use in various medical applications. Lactic acid and glycolic acid units can be randomly distributed in the polymer or prepared as copolymers. This modification capability allows PLGA to achieve versatility for different applications.^[^ ^478^ ^]^ Song et al. combined an inorganic therapeutic drug, arsenic trioxide (As2O3), with PLGA NMs, incorporating a degree of targeting through lactobionic acid conjugation. However, compared to arsenic trioxide monotherapy, the increase in antitumor activity and reduction in side effects were not significant.^[^ ^479^ ^]^ Modifying PLGA NMs with targeting ligands is crucial for improving delivery efficiency. A novel FA‐modified alginate‐PLGA NM system has been developed for targeted delivery of DOX to LC cells (HepG2 and Huh7). After 24 h of treatment, the cell viability of HepG2 and Huh7 cells significantly decreased to ≈12% and 10%, respectively, with an IC50 of 100 nM.^[^ ^480^ ^]^ Furthermore, numerous PEG‐PLGA copolymers are frequently used in LC therapy. Aside from their FDA approval, PEG‐PLGA copolymers show significant potential in controlled drug release. Zheng et al. developed a dual‐drug delivery system containing Sor and metapristone to overcome and prevent resistance. This system showed an initial burst release of 40–50% of the drug within a few hours, followed by a slow, sustained release, with 30% of the drug released over 10 days.^[^ ^451^ ^]^ PEG‐PLGA copolymers provide a promising model for future systems aimed at sustained release.

Polysaccharides are widely used as functional carriers for constructing nano‐drug delivery systems owing to their excellent bioactivity, biocompatibility, biodegradability, inherent targeting abilities, natural abundance, low immunogenicity, and large chemical modification space.^[^ ^481^, ^482^ ^]^ In recent years, polysaccharides have been used as functional carriers to construct NMs loaded with chemotherapy drugs in some instances, showing strong antitumor activity against LC. Compared to free DOX, DOX/FA‐mediated and SA‐modified Bletilla striata polysaccharide (BSP) nanomicelles (DOX/FA‐BSP‐SA) showed stronger inhibitory effects on HepG2 cells. DOX/FA‐BSP‐SA also prolonged the in vivo retention time of DOX and enhanced its bioavailability.^[^ ^453^ ^]^ The use of polysaccharides as functional carriers for natural compounds in NMs to improve the bioavailability and anticancer efficacy of these compounds has also been studied. Guo et al. developed a biomimetic NM (GACS‐Cur@RBCm) by combining curcumin (Cur) with functionalized angelica polysaccharide (APS). In LC treatment, this NM enhanced drug accumulation in tumor tissue through a synergistic effect between APS targeting the mannose/galactose receptor and glycyrrhetic acid (GA) targeting GA receptors. Notably, the RBCm coating extended the in vivo circulation time. The application of GACS‐Cur@RBCm activated antitumor immune responses, increasing CD8^+^ T‐cell infiltration by 1.9‐fold and significantly elevating serum levels of IL‐12, TNF‐α, and IFN‐γ. This drug releases Cur and its prodrug in response to TME GSH, synergistically regulating the immune microenvironment to inhibit LC progression and reduce systemic toxicity.^[^ ^454^ ^]^

CS is a positively charged heteropolysaccharide and a natural polymer. Upon contact with water, the amino groups of CS become protonated, allowing it to dissolve in acidic aqueous solutions. A major limitation of CS is its poor solubility. Therefore, methods to modify CS, including quaternization and the use of grafting agents, have been explored. CS‐based biomaterials have become a suitable choice for LC, owing to their unique properties such as biodegradability, low toxicity, and high biocompatibility. In basic drug delivery, studies have shown that CS NMs loaded with Sor exhibit higher cytotoxicity against HepG2 cells compared to Sor alone, while exhibiting low toxicity toward normal cells, thus indicating high biocompatibility.^[^ ^483^ ^]^ Moreover, the drug delivery efficiency, biosafety, and functional complexity of NMs can be further improved by modifying CS particles. Tim‐3 is a novel immune checkpoint biomolecule and a potential target for LC therapy. A pH‐responsive drug‐eluting NM based on carboxymethyl CS (CC@SR&SF@PP) has been developed for the co‐delivery of Tim‐3 siRNA and Sor to LC, using the charge inversion properties of CMCS for targeted LC treatment. Sor is first loaded into positively charged mPEG5KPAE10K (PP) NMs triggered by pH, then conjugated with negatively charged Tim‐3 siRNA. Finally, the NM surface is adsorbed with CMCS. This NM enhances the simultaneous release of siRNA and Sor. The enhanced Tim‐3 siRNA can inhibit tumor cell growth by inducing immune responses and recruiting CTLs, whereas the SF@PP NM releases Sor at lower pH, blocking tumor proliferation and angiogenesis. This design leverages CMCS to protect gene drugs in physiological conditions, triggers synergistic drug release in the acidic microenvironment of the tumor, and significantly enhances the delivery efficiency of Tim‐3 siRNA and the anti‐angiogenesis effect of Sor to inhibit in situ LC progression.^[^ ^455^ ^]^

### 6.5 Hydrogels

**Figure 14:**
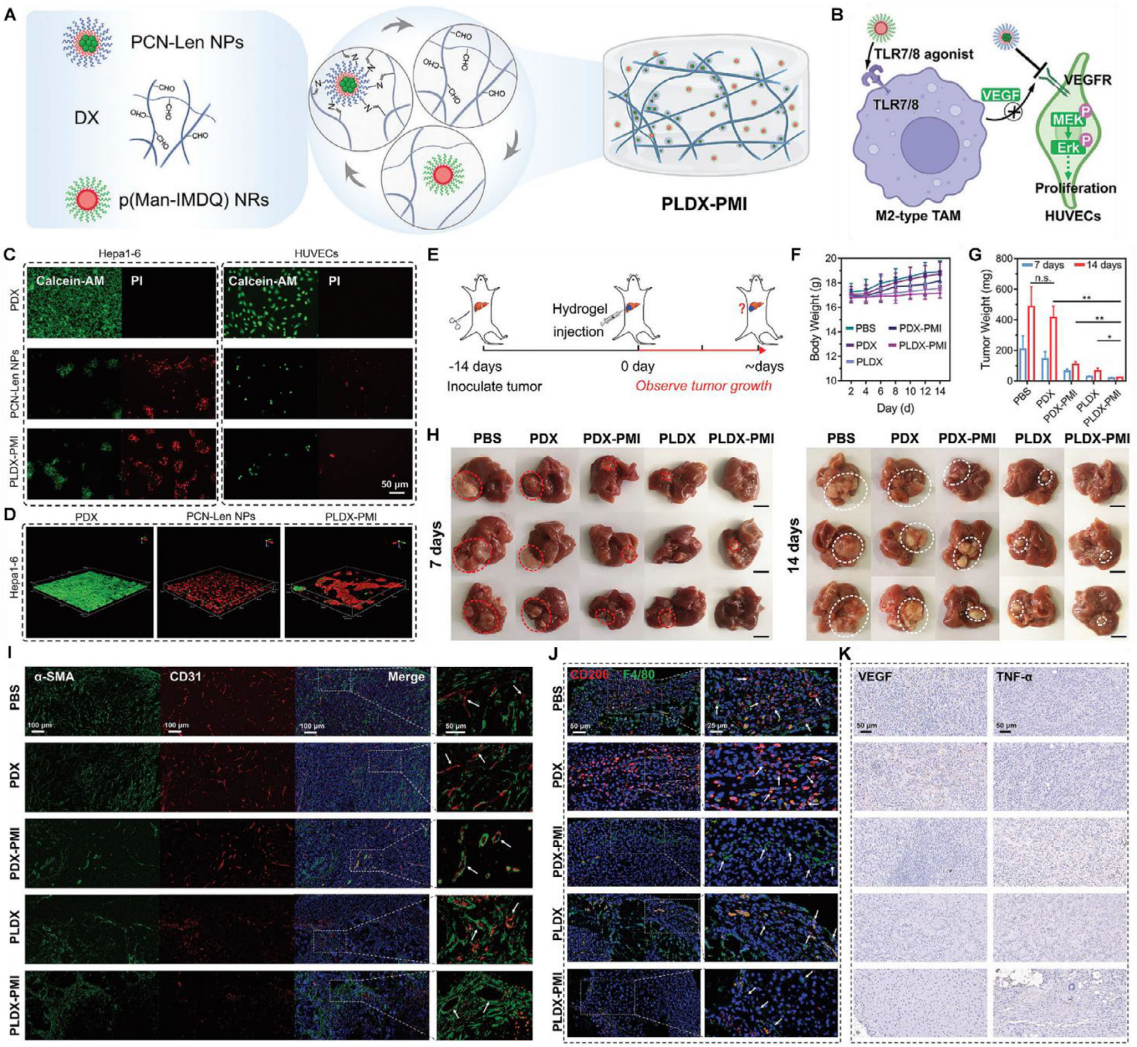
A) Supramolecular hydrogel PLDX‐PMI composed of drug‐loaded PCN‐Len NMs, hydrogel backbone DX, and TAM‐targeted p(Man‐IMDQ) NRs. B) Schematic of the mechanism by which PCN‐Len NMs and p(Man‐IMDQ) NRs inhibit endothelial cell proliferation. C) Representative CLSM images of live/dead staining in Hepa1‐6 cells/HUVECs treated with PDX, PCN‐Len NMs, and PLDX‐PMI hydrogel. D) 3D reconstruction of CLSM images of Hepa1‐6 cells treated with PDX, PCN‐Len NMs, and PLDX‐PMI hydrogel. E) In situ Hepa1‐6 LC treatment protocol. F) Mouse body weight curve. Data are presented as mean ± SD (*n* = 5). G) Tumor weight on days 7 and 14 after treatment. Data are presented as mean ± SD (*n* = 3). H) Photographs of excised tumor‐bearing livers on days 7 and 14 post‐treatment. I) PCN‐Len NMs and p(Man‐IMDQ) NRs effectively reduce vascular density in LC tumor tissue. Representative images of CD31 (red) and α‐SMA (green) staining of in situ Hepa1‐6 LC tissue vasculature, with DAPI nuclear staining. J,K) PCN‐Len NMs and p(Man‐IMDQ) NRs induce M2‐type TAM reprogramming and promote anti‐angiogenesis. J) Representative immunofluorescence images of CD206 (red)/F4/80 (green) staining in tumor tissue. K) Representative immunohistochemical images showing VEGF and TNF‐α expression in tumor sections from mice treated with PBS, PDX, PDX‐PMI, PLDX, and PLDX‐PMI. Reproduced (Adapted) with permission.^[^ ^458^ ^]^ Copyright 2023, John Wiley and Sons.

Hydrogels are 3D polymeric networks capable of absorbing and retaining large quantities of water within their matrix. Owing to their excellent biocompatibility, biodegradability, flexibility, and multifunctionality, hydrogels are considered ideal platforms for biomedical applications.^[^ ^484^ ^]^ They can serve as stabilizers or scaffolds for NMs, enhancing their stability and preventing aggregation and oxidation. The 3D hydrogel matrix can be functionalized with specific chemical groups to load a wide range of biomolecules and therapeutic agents.^[^ ^485^ ^]^ However, the incorporation of NMs may influence the mechanical strength and swelling behavior of the hydrogel. Nano‐hydrogel composites integrate the advantages of NMs into hydrogel systems, enabling the construction of hierarchical structures that allow for the controlled, multi‐phase release of therapeutic agents and enhance the synergistic effects of combination therapies.^[^ ^486^, ^487^ ^]^ For example, Zhao et al. developed a supramolecular peptide hydrogel as a local sustained‐release carrier for triptolide to treat orthotopic LC through intraperitoneal administration. The hydrogel, based on C16GNNQQNYKD‐OH, exhibited stronger cytotoxicity toward LC cells than normal hepatocytes and significantly inhibited tumor growth. It remained in the peritoneal cavity for ≈2 weeks post‐injection, thereby prolonging the therapeutic effect.^[^ ^457^ ^]^ Recently, a supramolecular hydrogel system was developed to deliver Len‐loaded NMs (PLDX‐PMI) with synergistic and sustained‐release properties. This system improved the efficacy of anti‐angiogenic therapy in orthotopic LC by reprogramming TAMs. By targeting endothelial tyrosine kinases, inhibiting tumor angiogenesis, and reducing microvascular density, it effectively suppressed the progression of Hepa1‐6 tumors. Furthermore, when hydrophilic polymannose (a macrophage‐targeting ligand) was combined with self‐assembling NMs, pro‐angiogenic M2 phenotype TAMs were reprogrammed into anti‐angiogenic M1phenotype TAMs (**Figure** **14**).^[^ ^458^ ^]^

To maximize their efficacy as drug delivery systems, hydrogel nanocomposites must exhibit several essential characteristics. As summarized by Palocci et al., these include high mechanical strength and injectability to improve in vivo drug stability and retention at the administration site. Moreover, they should possess structural tunability and strong drug–matrix interactions to allow controlled and drug‐specific release profiles. Ideally, the hydrogel matrix should also support the co‐delivery of multiple therapeutic agents to address the complexity of the TME and achieve synergistic treatment effects.^[^ ^488^ ^]^

### 6.6 Exosomes

**Figure 15:**
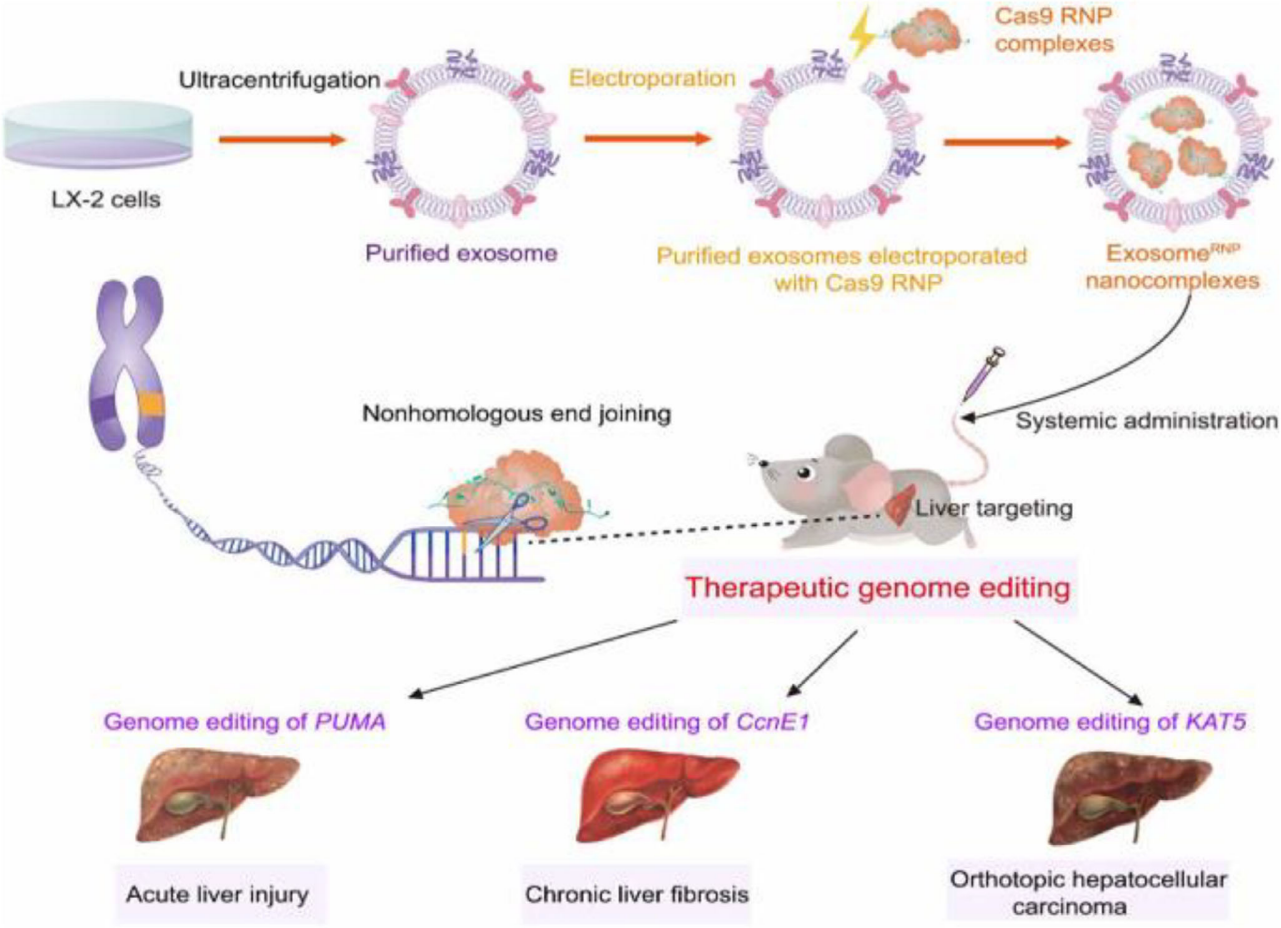
Schematic of exosome‐mediated in vivo delivery of Cas9 RNP for the treatment of liver diseases. Reproduced (Adapted) with permission.^[^ ^460^ ^]^ Copyright 2015, American Association for the Advancement of Science.

Extracellular vesicles, particularly exosomes, are single‐membrane vesicles with diameters typically ranging 30–200 nm. They play a crucial role in intercellular communication and material transport. One key function of exosomes is to deliver bioactive molecules, such as nucleic acids and proteins, into the cytoplasm of recipient cells, facilitating critical biological exchanges.^[^ ^489^ ^]^ Owing to their high biocompatibility, low immunogenicity, and efficient delivery properties, exosomes have garnered increasing interest. exosomes are secreted under both physiological and pathological conditions, indicating their broad cellular origins. This widespread production underlies their potential for clinical applications. Recent advances in NM research have highlighted exosome‐based NMs as promising delivery vehicles. These systems have shown the ability to carry and modify various therapeutic cargos, including small molecules, nucleic acids, and peptides, providing novel opportunities in LC therapy.^[^ ^490^ ^]^ For example, Dox was encapsulated into NK cell‐derived exosomes (NK‐exos‐Dox). Owing to the inherent cytotoxic proteins present in NK‐exos, these NMs enhanced LC‐targeting efficiency. NK‐exos‐Dox synergistically activated granzyme B/perforin‐mediated cytotoxic pathways and the mitochondrial apoptosis pathway (Bax↑/Bcl‐2↓), while releasing Dox, resulting in a 2.1‐fold increase in apoptotic rate compared to free Dox.^[^ ^459^ ^]^ In another study, exosomes provided a solution for delivering large therapeutic payloads such as Cas9/sgRNA ribonucleoprotein (RNP) complexes, which exceed the loading capacity of conventional delivery systems. Wan et al. used electroporation to load RNP into exosomes isolated from HSCs, generating liver‐specific therapeutic exosomes (ExosomeRNP) with gene‐editing capabilities. In vivo experiments showed that ExosomeRNPs selectively accumulated in mouse livers and effectively treated LC and other hepatic diseases (**Figure** **15**).^[^ ^460^ ^]^

In addition to serving as drug delivery vehicles, exosomes derived from specific cell types, such as tumor cells, exhibit potent immunostimulatory functions. Zou et al. isolated tumor‐derived exosomes carrying tumor‐associated antigens, which improved DC recognition and enhanced the immunogenic potential of LC vaccines. To further increase their immunogenicity, the exosome surface has been functionalized with the potent adjuvant high mobility histone nucleosome binding protein 1. This strategic modification enhanced DC‐mediated T cell activation, thereby improving vaccine efficacy and augmenting the therapeutic effect of LC immunotherapy.^[^ ^461^ ^]^ Although the roles of exosomes in tumor progression, therapy, and biomarker development have been extensively investigated, several challenges remain. These include the need for standardized isolation and characterization methods, scalable production, improved drug loading and targeting efficiency, and ensuring safety for systemic administration.^[^ ^491^ ^]^

### 6.7 Nanovaccines

Tumor vaccines introduce TAAs into patients in the form of genes encoding tumor antigens, tumor cells, or tumor‐associated proteins and peptides. These vaccines are designed to overcome tumor‐induced immunosuppression, activate the patient's immune system, and induce both cellular and humoral immune responses, thereby enhancing immunogenicity to achieve tumor control or eradication.^[^ ^492^ ^]^ According to the classification of the Society for Tumor Immunotherapy, current therapeutic tumor vaccines can be categorized into four major types: autologous tumor cell vaccines, peptide vaccines, dendritic cell or antigen‐presenting cell vaccines, and vector‐based vaccines.^[^ ^493^ ^]^ The immunomodulatory effects of the above tumor vaccines depend on their successful delivery and therapeutic efficacy. The emergence of nanotechnology has provided significant advantages in the development of tumor vaccines for LC. Shi et al. developed a functional nanovaccine (TRZ) by co‐loading the hypoxia‐activated prodrug tirapazamine (TPZ) and the immune adjuvant resiquimod (R848) into ZIF‐8 NPs. The formulation was further encapsulated in a porous microsphere to produce an embolic agent (TRZM). In a rabbit VX2 orthotopic liver tumor model, intra‐arterial administration of TRZM achieved synergistic chemoembolization–immunotherapy. The system triggered the specific release of TPZ and R848 within the hypoxic tumor microenvironment, enhanced tumor cell killing, increased the apoptosis rate to 75.3%, and induced ICD. Application of TRZM remodeled the LC immune microenvironment by markedly increasing CTL infiltration, elevating the proportion of CD8⁺ T cells by 2.1‐fold, and promoting M2‐to‐M1 macrophage polarization, thereby effectively suppressing primary LC growth.^[^ ^462^ ^]^

Nucleic acid‐based nanovaccines represent a paradigm shift in modern vaccinology. Their fundamental advantage lies in transforming human cells into in situ “bioreactors” for antigen production, thereby bypassing the laborious and costly in vitro manufacturing and purification steps required for traditional vaccines. The core appeal of nucleic acid nanovaccines stems from their flexible design and potent immunostimulatory potential.^[^ ^492^, ^494^ ^]^ With precisely engineered NMs, these vaccines not only protect and efficiently deliver fragile nucleic acid molecules but also enhance immune responses through their inherent adjuvant effects. This platform offers unprecedented opportunities for combating rapidly mutating pathogens and developing personalized cancer vaccines.^[^ ^495^, ^496^ ^]^ Currently, the most common forms of nucleic acid nanovaccines are DNA and mRNA vaccines. Wu and colleagues developed a spleen‐targeted nanovaccine (RBC‐Nanovaccine) by encapsulating neoantigen DNA within polymer–lipid NPs (pDNA‐NPs) and utilizing a RBC hitchhiking strategy. In LC immunotherapy, administration of this nanovaccine enabled spleen‐specific accumulation, increasing the spleen‐to‐lung accumulation ratio by 5.1‐fold. It enhanced neoantigen expression, achieving a transfection efficiency 2.2 times higher than that of the control group, and promoted cytotoxic T‐cell activation, with CD8⁺ T‐cell infiltration levels increasing by 2.1‐fold. Additionally, it induced ICD and long‐term immune memory. The application of RBC‐Nanovaccines remodeled the tumor immune microenvironment by reducing the proportion of Tregs and promoting M1 macrophage polarization, thereby suppressing LC progression. Tumor volume was reduced by 95%, lung metastases decreased by 80%, and in combination with anti–PD‐1 antibodies, complete tumor regression was achieved with a 75% complete remission rate.^[^ ^463^ ^]^

mRNA vaccines have garnered considerable attention following their success in COVID‐19 prevention and hold significant potential for cancer immunotherapy, including LC prevention and treatment. mRNA‐based vaccines direct host cells to synthesize tumor‐associated proteins or antigens, thereby eliciting immune responses against cancer cells.^[^ ^497^ ^]^ Zhang et al. developed a tumor RNA vaccine (RNA‐LNPs) by encapsulating total RNA extracted from LC cells into LNPs. In LC immunotherapy, RNA‐LNPs promoted DCs maturation, which in turn activated tumor antigen‐specific CD8⁺ and CD4⁺ T cells. Mature DCs provided costimulatory signals through endogenous antigen processing and presentation pathways and activated CD8⁺ T cells via cross‐presentation, effectively suppressing tumor growth. In both prophylactic and therapeutic mouse models, tumor volume was reduced by ≈50%.^[^ ^336^ ^]^

Given the complexity of tumor initiation and progression, developing effective cancer vaccines remains a formidable challenge. Because cancer cells closely resemble normal host cells rather than foreign pathogens, they often evade immune surveillance. Furthermore, the unique antigenic profiles of individual tumors require more sophisticated vaccine design strategies. It is therefore essential to explore optimal ways to apply nanotechnology in the development of cancer vaccines. In addition, combining tumor vaccines with other therapeutic approaches, such as ICIs, may provide a more effective treatment paradigm.^[^ ^498^ ^]^ Vaccines can enhance the efficacy of checkpoint blockade therapy by promoting immune recognition of tumor antigens and strengthening antitumor immune responses.

## 7 Progress in Clinical Trials of NMs for the Treatment of LC

Compared with other solid tumors, such as brain and pancreatic cancers, LC offers distinct advantages for localized drug administration. These advantages include the feasibility of intra‐arterial delivery through interventional transcatheter techniques and the possibility of achieving active targeting via ligand–receptor interactions.^[^ ^499^ ^]^ As a result, NM‐based drug delivery shows great promise for the treatment of LC, and several platforms, including the GalNAc–siRNA conjugate system, have already demonstrated clinical success.^[^ ^500^, ^501^ ^]^ Despite encouraging preclinical results indicating the therapeutic potential of NMs in LC, their translation into clinical practice remains a significant challenge. This section provides an overview of currently approved and investigational NMs under clinical trials (**Table** **4**) and examines the existing gap between preclinical research and clinical application.

**Table 4:** Clinical Trials of NMs for the Treatment of LC.

Title	NMs	Active agent	Clinical outcome measure	Phase	NCT	Refs.
Study of Pegylated Liposomal Doxorubicin and Temsirolimus in Patients With Advanced Hepatocellular Cancer	Doxil^®^	liposomal DOX	i) Progression‐free survival ii) Toxicity and tolerability iii) Overall survival	II	NCT01281943	[518]
HAI Abraxane With Gemcitabine and Bevacizumab	Abraxane^®^	Nab‐paclitaxel	i) Maximum Tolerated Dose of Escalating Doses of Hepatic Arterial Infusions of Abraxane in Combination with Gemcitabine and Bevacizumab	I	NCT01057264	[519]
NBTXR3 Crystalline Nanoparticles and Stereotactic Body Radiation Therapy in the Treatment of Liver Cancers.	NBTXR3	HfO2 NPs	i) Treatment‐related adverse events ii) Response Rate iii) Local Progression Free Survival	II	NCT02721056	[508]
PD‐1 mRNA LNP Vaccine for Advanced Primary Hepatocellular Carcinoma.	PD‐1 mRNA LNP	PD‐1 mRNA	i) Disease Control Rate ii) progression‐free survival iii) Overall Survival	II	NCT07053072	[509]
Mts105 for Advanced Hepatocellular Carcinoma (MTS105 for HCC)	Mts105 (mRNA LNP)	therapeutic protein mRNA	i) Peak Plasma Concentration ii) Objective Response Rate iii) Overall Survival	I	NCT06689540	[520]
WGc‐0201 Plus Tislelizumab in HCC With High Risk of Recurrence and Metastasis After Radical Therapy	WGc‐0201 (HBV mRNA LNP)	HBV mRNA	i) Recurrence‐Free Survival ii) Survival rate iii) Treatment‐related adverse events	I	NCT07077369	[521]
Phase Ib/​2, Multicenter, Dose Escalation Study of DCR‐MYC in Patients With Hepatocellular Carcinoma	DCR‐MYC	MYC SiRNA	i) DCR‐MYC Biological Activities ii) DCR‐MYC Levels in Blood	I /II	NCT02314052	[262]
A Phase I Trial of IMA970A Plus Montanide in Combination With Durvalumab (Anti‐PD‐L1)	IMA970A	Peptide Vaccine	i) Disease‐free survival ii) Overall survival iii) immunological parameters in blood	I	NCT06218511	[522]
GNOS‐PV02 Personalized Neoantigen Vaccine, INO‐9012 and Pembrolizumab in Subjects with Advanced HCC	GNOS‐PV02	DNA Vaccine	i) Objective Response Rate ii) Duration of Response iii) Disease Control Rate iv) Overall Survival	I/II	NCT04251117	[510]

At present, only a few NMs have been specifically approved for LC, although several nanotechnology‐based formulations have received regulatory approval from the U.S. FDA or the European Medicines Agency (EMA) for improving pharmacokinetics and tumor targeting. Liposomal DOX (Doxil) is one of the most established NMs. Through the EPR effect, Doxil accumulates within tumors and has been incorporated into LC chemotherapy regimens. When combined with TACE, Doxil enhances local drug concentration while reducing systemic toxicity.^[^ ^502^, ^503^ ^]^ Doxil has also been widely used in other malignancies, including ovarian cancer.^[^ ^504^ ^]^ Nab‐paclitaxel, although primarily approved for pancreatic and breast cancers, has shown moderate efficacy in clinical trials for LC by improving paclitaxel solubility and tumor targeting, particularly in combination with immunotherapy or chemotherapy.^[^ ^505^ ^]^ The GalNAc–siRNA conjugate platform, initially developed for genetic liver disorders such as transthyretin amyloidosis, enables hepatocyte‐specific targeting through ASGPR‐mediated uptake.^[^ ^501^ ^]^ Givosiran (Givlaari) utilizes GalNAc modifications to achieve efficient hepatic delivery, providing a foundation for RNA interference‐based therapies against LC‐related targets.^[^ ^506^ ^]^ Although these drugs have not been directly approved for LC, their underlying platform technologies are being extended to LC clinical trials.

A growing number of clinical studies are evaluating the safety and efficacy of NMs in LC, particularly in immunomodulation, targeted delivery, and combination therapy. Hafnium oxide NPs (NBTXR3) represent a novel radioenhancer that has entered several clinical trials for soft‐tissue sarcoma. A phase II study investigated the intratumoral or superselective intra‐arterial administration of NBTXR3 combined with stereotactic body radiation therapy in LC patients, demonstrating promising safety and therapeutic potential.^[^ ^507^, ^508^ ^]^ Most ongoing trials, however, focus on nanovaccines based on mRNA or siRNA for expressing tumor neoantigens or immune modulators. For instance, the NCT07053072 trial evaluated an mRNA vaccine encoding the PD‐1 protein (PD‐1 mRNA LNP) for primary LC, assessing its safety, tolerability, immunogenicity, and preliminary efficacy.^[^ ^509^ ^]^ DNA‐based vaccines are also being investigated. The NCT04251117 trial evaluated a personalized neoantigen DNA vaccine (GNOS‐PV02) in combination with a PD‐1 inhibitor for advanced LC. Among 36 enrolled patients, the ORR reached 30.6% (11 of 36), including 8.3% (3 of 36) complete responses, indicating clinical promise but requiring further optimization.^[^ ^510^, ^511^ ^]^

Despite the notable antitumor efficacy observed in several clinical studies, including tumor regression, remodeling of the immune microenvironment, and the induction of durable immune memory, the clinical translation of NMs remains limited and is expected to require an additional five to ten years for widespread application.^[^ ^511^, ^512^ ^]^ The primary obstacles involve toxicity and biosafety concerns, as the small particle size and large surface area of NMs may elicit unpredictable immunogenicity, induce organ accumulation leading to oxidative stress or fibrosis, and cause off‐target effects that result in nonspecific tissue distribution.^[^ ^513^, ^514^ ^]^ Even when safety issues are adequately addressed, large‐scale manufacturing continues to present challenges such as maintaining batch‐to‐batch consistency, optimizing encapsulation efficiency, and ensuring the stability of LNPs. In addition, RNA‐based NMs typically require cold‐chain storage, which restricts their use in regions with limited resources.^[^ ^515^ ^]^ Regulatory and clinical trial design issues further hinder translation, as extensive data are necessary to prove superiority over existing therapeutic options. Most current clinical trials evaluate NMs as adjuvant treatments, and improvements in patient selection criteria and clinical endpoints will likely depend on expensive and time‐consuming phase III studies. Moreover, the pronounced heterogeneity of LC and its immunosuppressive TME make therapeutic outcomes difficult to predict. Commonly used preclinical models, including immunodeficient mice, do not accurately reproduce the human immune system, which may explain why robust T‐cell responses observed in animal experiments often diminish in humans due to T‐cell exhaustion or infiltration of Tregs.^[^ ^516^, ^517^ ^]^ Overall, these factors represent the principal bottlenecks that continue to impede the successful clinical translation of NMs for LC.

## 8 Analysis of the Challenges of NMs

NMs show significant potential in LC treatment, particularly in targeted drug delivery and immunoregulation. By using the unique properties of NMs, drugs can be delivered more precisely to tumor sites, thereby reducing adverse effects on healthy tissues. However, despite their promising therapeutic potential, the toxicity and side effects of NMs remain key challenges that need to be addressed. The toxicity of NMs is closely related to their physicochemical properties and interactions with biological systems. Their small size and high surface area allow them to interact directly with cells and biomolecules after entering the body, leading to a range of side effects.^[^ ^523^ ^]^ For example, AgNPs, when decomposed in the body, release silver ions that can induce oxidative stress, resulting in cell membrane damage and DNA damage. This oxidative stress damages cellular structures and interferes with the functions of essential biomolecules, thus causing cellular dysfunction.^[^ ^524^, ^525^ ^]^ In addition, the surface characteristics of NMs, such as charge, hydrophilicity, or hydrophobicity, can influence their interaction with the immune system.^[^ ^526^, ^527^ ^]^ Some surface‐modified NMs may activate the immune system, triggering unnecessary immune responses, which could lead to immunosuppression or immune evasion, thus affecting LC treatment outcomes.

The distribution and accumulation of NMs within the body also significantly influence their toxicity. Because of differences in metabolic rates and clearance, some NMs may accumulate in specific organs, causing toxic reactions in those organs.^[^ ^528^ ^]^ Particularly for NMs with electronic, optical, or magnetic properties, their breakdown may induce unique and unpredictable toxic effects. Accumulation in the liver is notably significant, as the liver is a primary organ for NM deposition. Long‐term accumulation may lead to hepatotoxicity, and in severe cases, liver damage or failure.^[^ ^529^ ^]^ Similarly, the kidneys, as the major excretory organs, may experience functional burden if NMs are not effectively eliminated, potentially resulting in renal damage. Therefore, optimizing the in vivo distribution of NMs, reducing accumulation in non‐target organs, and enhancing renal clearance are critical strategies for minimizing toxicity.^[^ ^530^, ^531^ ^]^ Moreover, the toxicity of NMs extends beyond their accumulation. Because of their small size and high stability, some NMs can penetrate cellular membranes, reaching organelles such as mitochondria, the endoplasmic reticulum, lysosomes, and nuclei. Once inside these organelles, they may catalyze chemical reactions with biomolecules, altering the normal 3D structure of biomolecules and biological membranes.^[^ ^532^ ^]^ This can lead to the inactivation of certain hormones and important enzymatic systems, causing severe biological damage inside the cell.^[^ ^533^ ^]^ More specifically, NMs may trigger a series of biological effects such as inflammatory responses, apoptosis, cell cycle alterations, and abnormal gene expression, which can damage the lungs, cardiovascular system, and other vital organs.^[^ ^534^ ^]^ This damage influences the efficacy of cancer treatment and may lead to other serious clinical complications.

The potential off‐target effects of NMs are another critical concern. Although NMs have shown the potential to reduce off‐target toxicity in targeted therapy, their targeting efficiency is not perfect, which may result in accumulation at non‐target sites. For instance, certain NMs can reach areas that larger particles cannot because of their smaller size, which enables drug delivery to tumor sites through targeted therapy. However, this also increases their accumulation in non‐target tissues.^[^ ^535^ ^]^ NMs may induce toxicity in normal tissues and even activate the immune system because of their small size and biocompatibility, thereby triggering autoimmune responses. These autoimmune reactions may lead to inflammation, necrosis, or other adverse effects in local tissues, further compromising treatment outcomes.^[^ ^536^ ^]^ In summary, despite the advantages of NMs in targeted delivery, their off‐target effects remain a significant challenge for clinical application.

The impact of NMs on the immune microenvironment is another critical aspect of the toxicity. By modulating the TME, NMs can enhance anti‐tumor immune responses, aiding in tumor immune clearance.^[^ ^537^ ^]^ However, this process may also induce excessive immune reactions, potentially leading to immune evasion and reducing therapeutic efficacy. Some NMs may induce inflammation in the TME. Although this inflammation may improve drug delivery efficiency in the short term, chronic inflammation over the long term may promote tumor growth and metastasis.^[^ ^538^ ^]^ Thus, balancing enhanced therapeutic efficacy, while avoiding immune toxicity and chronic inflammation is a major challenge in current research.

After intravenous administration, NMs rapidly interact with blood components, among which the unintended activation of the complement system represents a frequently overlooked yet critical safety concern.^[^ ^539^ ^]^ Complement activation can trigger the release of anaphylatoxins such as C3a and C5a, leading to infusion‐related reactions, including flushing, dyspnea, and even anaphylactic shock, thereby severely limiting clinical application. The surface properties of NMs, such as hydrophobicity and positive charge, are key drivers of complement activation. For instance, although PEGylation is commonly employed to prolong circulation time, anti‐PEG antibodies present in certain individuals may induce the accelerated blood clearance effect and activate the complement system. In addition, ionizable lipids used in some LNPs may also exhibit adjuvant‐like immunostimulatory activity.^[^ ^540^ ^]^ Therefore, immunological safety should be considered a central design parameter in the development of NMs. Comprehensive in vitro complement activation assays and in vivo immunotoxicity evaluations should be conducted, along with active exploration of more biocompatible surface chemistries, such as zwitterionic coatings, to prevent or mitigate complement activation.^[^ ^541^ ^]^

NMs represent complex “systems” whose efficacy, safety, and pharmacokinetic properties depend not only on the active pharmaceutical ingredient itself but also on the physicochemical characteristics of the nanocarrier.^[^ ^542^ ^]^ Currently, there is no mandatory and standardized characterization framework in either academia or industry for the comprehensive evaluation of NMs, making it difficult to directly compare data generated by different laboratories or companies. For example, particle size can be measured using dynamic light scattering (DLS), transmission or scanning electron microscopy, or nanoparticle tracking analysis; however, due to the different principles underlying these techniques, the resulting measurements may vary substantially. More importantly, the assessment of in vitro drug release kinetics often lacks standardized media and conditions that are physiologically relevant, limiting the predictive power of in vitro data for in vivo release behavior. For complex biomimetic NMs, accurately characterizing the density, orientation, and activity of surface ligands remains a major analytical challenge.^[^ ^543^ ^]^ Therefore, establishing a “minimum information standard” for NMs characterization and promoting the development of advanced in vitro models that better simulate the in vivo environment, including protein adsorption and hemodynamic shear stress, is crucial for accurately predicting NMs in vivo performance.

The unique physicochemical properties and complex mechanisms of action of NMs pose new challenges to the existing drug regulatory approval framework. Global regulatory agencies, such as the US FDA and the EMA, are actively developing specialized guidelines for NMs, with key regulatory challenges spanning several areas.^[^ ^544^ ^]^ The primary challenge lies in the evaluation of bioequivalence. For generic NMs, relying solely on the chemical equivalence of the active pharmaceutical ingredient cannot ensure the consistency of in vivo distribution, efficacy, and safety compared to the reference product, thus presenting challenges to the applicability of traditional bioequivalence evaluation methods.^[^ ^545^ ^]^ Furthermore, toxicological evaluation is more complex, requiring a comprehensive assessment of the long‐term toxicity, tissue distribution, and elimination pathways of both the nanocarrier and its degradation products, which often necessitate longer toxicological studies than those required for traditional drugs.^[^ ^546^ ^]^ Additionally, the concept of quality by design has gained increasing attention, with regulatory agencies strongly recommending the identification of critical quality attributes early in the NMs development process, and the establishment of associated critical process parameters to ensure consistent product quality. Moreover, pharmacokinetic and pharmacodynamic evaluations face new challenges, as they require the establishment of new models capable of accurately characterizing the in vivo behavior of NMs, since their tissue distribution and clearance properties are not only dependent on the active ingredient but are also significantly influenced by the nanocarrier.^[^ ^547^ ^]^ Addressing these challenges requires close collaboration among academia, industry, and regulatory agencies to develop scientifically sound evaluation standards and new drug review pathways.

Although NMs show great potential in the treatment of LC, their application is still constrained by various challenges that need to be systematically addressed. These challenges include reducing nanotoxicity related to physicochemical properties, such as oxidative stress induced by ion release and organelle damage caused by deep cellular penetration. Additionally, optimizing surface modifications to enhance targeting specificity, reduce off‐target accumulation, and minimize unintended immune activation mediated by the complement system is crucial. Furthermore, improving metabolic pathways and clearance mechanisms is necessary to control the distribution and accumulation of NMs in the liver and kidneys, thereby preventing long‐term organ damage. The complex interactions between NMs and the immune microenvironment require precise regulation to maintain antitumor efficacy while avoiding chronic inflammation or immune escape. Advancing standardized characterization methods and addressing regulatory challenges are essential for ensuring reproducibility and clinical feasibility. Future breakthroughs rely on collaborative innovation across multidisciplinary fields, including nanotechnology, pharmacology, immunology, and regulatory science, to establish safe and effective NMs therapies and ultimately achieve their widespread use in LC treatment.

## 9 Summary and Future Perspective

The development of smart‐responsive NMs has provided a powerful tool for the treatment of LC. With growing insight into the immunosuppressive TME of LC, the complexity of liver tumor TME can be attributed to the interplay between abnormal physiological conditions, LC cells, and various immune‐related cells. This process also involves metabolic reprogramming and the participation of microbiota and their metabolites. As a result, the application of NMs has gradually evolved from simple drug delivery systems to multifunctional platforms capable of modulating immune responses.

Smart‐responsive NMs integrate passive targeting through the EPR effect, active targeting through surface ligands specific to receptors on LC or immune cells within the TME, and stimuli‐responsive release triggered by internal TME signals or externally applied stimuli. These targeting strategies overcome delivery barriers posed by the immunosuppressive TME, enhance local drug accumulation in the liver, improve therapeutic outcomes, and reduce off‐target toxicity. In combination with chemotherapy, immunotherapy, and RT, such NMs enable multimodal synergistic treatment strategies to address multiple therapeutic challenges. Another major advantage of smart NMs lies in their immunomodulatory capabilities. They can reverse immune tolerance in LC by reprogramming immune cell functions, reshaping abnormal microenvironments, suppressing ECM deposition, regulating metabolic reprogramming, inducing ICD, and modulating the gut microbiota and its metabolites. Moreover, these systems can be further enhanced with externally administered immunomodulators such as cytokines and ICB. These innovative strategies provide promising avenues for the precision treatment of LC. Among the various types of smart NMs, biomimetic nanomaterials show particularly high potential for developing personalized cancer vaccines. By coating nanovaccines with cell membranes derived from tumor or immune cells, these biomimetic NMs present diverse tumor antigens on their surfaces, thereby overcoming the limited antigenic stimulation of conventional vaccines and inducing durable, multivalent antitumor immunity. In addition, these NMs can target specific cell types and directly fuse with cell membranes, facilitating co‐delivery of immune adjuvants.^[^ ^548^ ^]^ Despite challenges such as limited predictive accuracy, high complexity, and elevated cost, biomimetic NMs provide a foundation for generating personalized vaccines based on the tumor antigens of individual patients.

The therapeutic application of stimuli‐responsive NMs for LC requires continuous innovation and optimization in nanotechnology as well as a deep understanding of the TME. The ultimate goal of NMs is to tailor treatment based on the complexity and heterogeneity of individual tumors, thereby maximizing patient benefits and achieving precision therapies. NMs designed to treat LC should be specifically engineered to address the distinct features of the TME, enabling personalized and targeted strategies, an emerging trend in LC management. With ongoing advancements in nanotechnology, material selection, and fabrication techniques, the biocompatibility and biosafety of NMs will likely continue to improve. High‐performance, multi‐modal, and stimuli‐responsive NMs are expected to evolve iteratively, thereby providing increasingly personalized and effective therapeutic options for patients with LC.

## 10 Conclusion

In conclusion, based on a comprehensive understanding of the immunosuppressive TME in LC, we highlight that NMs offer a transformative therapeutic paradigm to overcome this central clinical challenge. Their fundamental value lies in shifting from the conventional “monotherapy” approach to an integrated strategy that couples targeted drug delivery with immune microenvironment remodeling. Recent advances have focused on the rational design of intelligent NMs capable of responding to endogenous signals or exogenous stimuli within the TME. These systems not only achieve spatiotemporally controlled drug release with markedly reduced systemic toxicity but also act as potent immunomodulators that can actively reverse immunosuppression within the TME, converting “cold” tumors into “hot” ones. However, translating these laboratory innovations into clinical practice remains challenging. Future progress will rely heavily on deep interdisciplinary integration to address critical issues related to biosafety, large‐scale manufacturing, and regulatory approval. We anticipate that next‐generation NMs will be increasingly personalized and intelligent. With the aid of artificial intelligence‐assisted design, NMs are expected to advance toward individualized precision therapies, marking a transition from generalized NMs to personalized NMs. Ultimately, NMs represent not merely a new technology but a paradigm shift in therapeutic philosophy. Through continuous technological iteration and cross‐disciplinary collaboration, they are poised to usher in a new era of LC treatment, bringing unprecedented hope to patients.

## Author Contributions

J.L., H.L., and J.X. contributed equally to this work. M.N., J.S., and Y.R.D. contributed to the conception, design, and final approval of the manuscript. J.M.L. wrote the main manuscript and H.L. prepared Figures 1, 2, 3, 4, 5, 6, 7, 8, 9, 10, 11. J.X. prepared Tables 1, 2, 3, 4. All authors have read and approved the final version of the manuscript.

## Conflict of Interest

The authors declare no conflict of interest.
